# Supplementary material for: Glutamine sensing licenses cholesterol synthesis
Source: EMBO J. 2024 Oct 21;43(23):5837–56. doi: 10.1038/s44318-024-00269-0 (PMC11612431; doi:10.1038/s44318-024-00269-0)
Supplement: Supplementary file 1 — Appendix [file 44318_2024_269_MOESM1_ESM.pdf]

Appendix for

Glutamine sensing licenses cholesterol synthesis

Table of Contents:

Appendix Figure S1 (page 1)
Appendix Figure S2 (page 2)
Appendix Figure S3 (page 3)
Appendix Figure S4 (page 4)
Appendix Figure S5 (page 5)
Appendix Figure S6 (page 6)

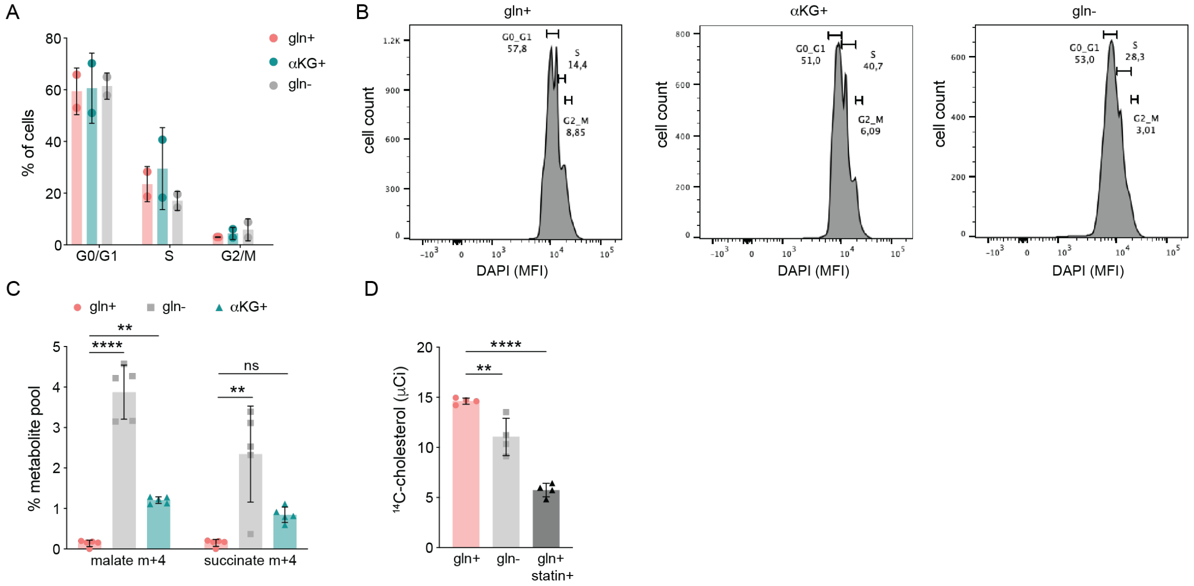

Appendix Figure S1: Effects of glutamine deprivation on glucose oxidation and acetate incorporation into cholesterol. (**A**) U2OS cells were cultured for 8h as indicated, fixed, and stained with DAPI for cell cycle analysis by flow cytometry. Data are mean ± s.d. of n=2 independent cultures; 100.000 events were analyzed per group. (**B**) Representative histograms of (**A**). (**C**) m+4 mass isotopologues of malate and succinate in U2OS cells cultured for 24h with 25 mM $^{13}C_6$-glucose in the presence of 2 mM glutamine (gln+) or its absence ± 1 mM αKG (gln-; αKG+ respectively). Data are mean ± s.d. of n=5 independent cultures, ns not-significant; **p<0.01; ****p<0.0001 by two-way ANOVA. (**D**) U2OS cells were cultured for 24h with 0.1 μCi/ml 14C-acetate in the presence of 2 mM glutamine ± 5 μM simvastatin (gln+, gln+ statin+; respectively) or its absence (gln-). Data are mean ± s.d. of n=4 independent cultures, ns not-significant; **p<0.01; ****p<0.0001 by one-way ANOVA.

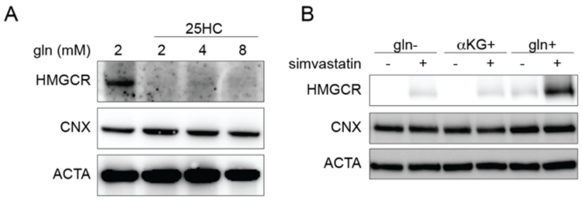

Appendix Figure S2: Effects of 25-hydroxycholesterol and simvastatin on HMGCR during glutamine excess and starvation, respectively. (**A**) U2OS cells were cultured in the presence of 2, 4, or 8mM of glutamine as indicated and 10 μM of 25-hydroxycholesterol (25-HC) for 24h. (**B**) U2OS cells were cultured w/ 2 mM of glutamine (gln+) or w/o gln ± 1 mM αKG (gln-; αKG+ respectively), and 5 μM simvastatin as indicated for 24h. (**A-B**) Samples were analyzed by immunoblotting for HMGCR, calnexin (CNX), and actin (ACTA).

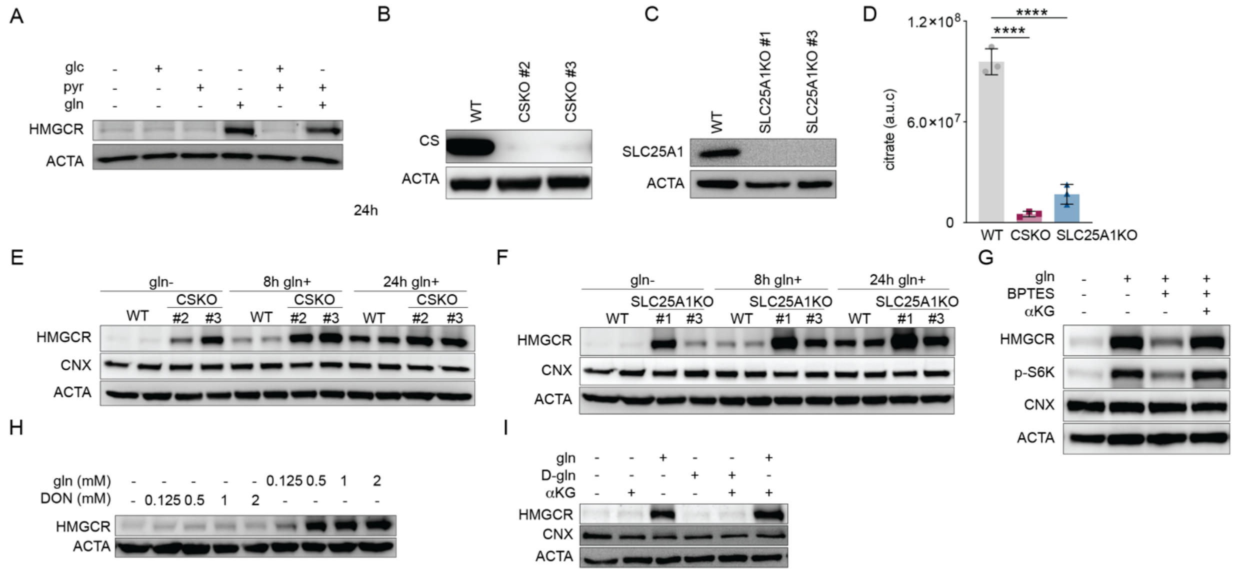

Appendix Figure S3: Glutamine, but not its derivatives nor analogues, increases HMGCR levels. (**A**) U2OS cells were cultured w/o glutamine for 24h and treated as indicated for 24h. Concentrations used: 1 mM sodium pyruvate (pyr), 2 mM glutamine (gln), and 25 mM glucose (glc). Samples were analyzed by immunoblotting for HMGCR and actin (ACTA). (**B-C**) U2OS cells of the indicated genotype were cultured with glutamine and analyzed by immunoblotting for citrate synthase (CS), solute carrier family 25 (SLC25A1), and actin (ACTA). (**D**) The abundance of total citrate in indicated U2OS genotypes. Data are mean ± s.d. of n=3 independent cultures, ****$p<0.0001$ by one-way ANOVA. (**E-F**) U2OSs with indicated genotypes were cultured in gln-free media for 24h, refed gln, and harvested at indicated times for analysis by immunoblotting for HMGCR, calnexin (CNX), and ACTA. For all experiments, glutamine was used at 2 mM and αKG at 1 mM. (**G**) U2OS cells were cultured as indicated for 8h. Concentrations used: 30 μM BPTES, 2 mM gln, 1 mM αKG. Samples were analyzed by immunoblotting for HMGCR, CNX, ACTA, and phospho-p70 S6 kinase (pS6K). (**H**) U2OS cells were cultured w/o glutamine for 24h and treated as indicated for 24h with gln or 6-diazo-5-oxo-L-norleucine (DON) with the indicated concentrations. Samples were analyzed by immunoblotting for HMGCR and ACTA. (**I**) U2OS cells were cultured w/o glutamine for 24h and treated as indicated for 24h with 2 mM gln, 2 mM D-gln or 1 mM αKG. Samples were analyzed by immunoblotting for HMGCR, ACTA, and CNX.

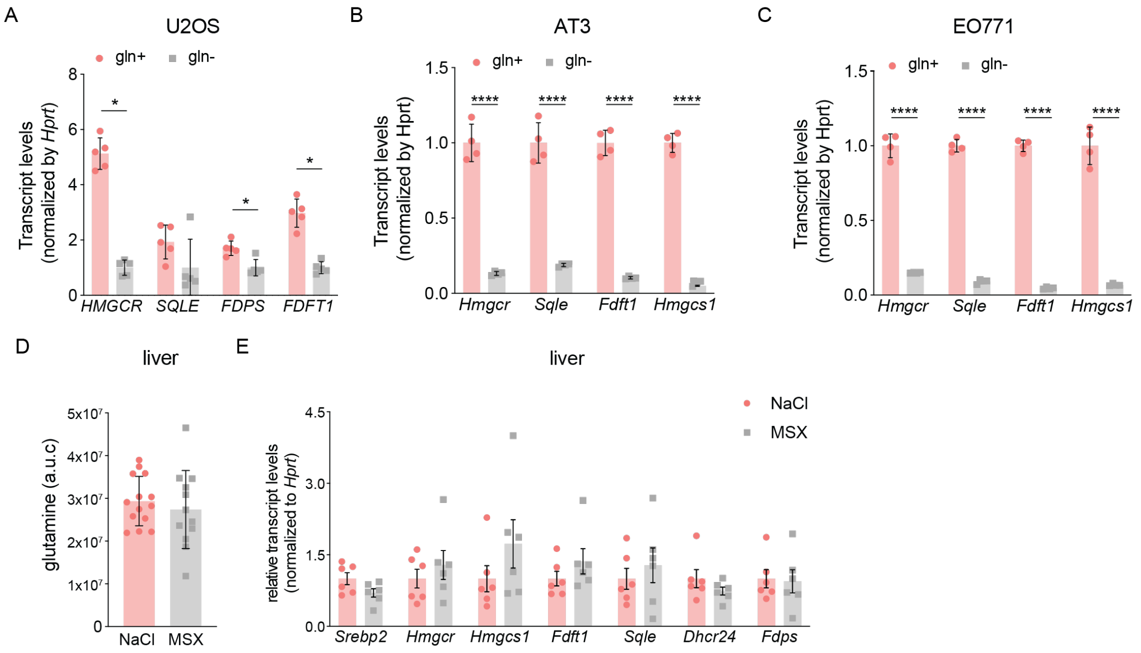

Appendix Figure S4: Glutamine starvation and MSX-treatment does not affect hepatic glutamine levels nor SREBP2 target transcripts. (**A**) U2OS cells were cultured with or w/o glutamine for 24h and analyzed by qPCR for the SREBP2 target genes. Transcript levels were normalized to *Hprt1* levels and are relative to gln-. Data are mean ± s.d. of n=5 independent cultures, *p<0.05; **p<0.01; ***p<0.001; ****p<0.0001 by multiple unpaired t-tests. (**B**) AT3 and (**C**) EO771 cells were starved to glutamine (gln) for 24h and then treated with or w/o gln for 24h. Transcript levels were normalized to *Hprt1* levels and are relative to gln-. Data are mean ± s.d. of n=4 independent cultures, ****p<0.0001 by multiple unpaired t-tests. (**D**-**E**) 10-week old C67Bl/6J mice were fed a 1.8% glutamine-containing or glutamine-free diet, and intraperitoneally injected with 0.9% saline or 20mg/kg MSX in a 48 hour interval, respectively. Mice were sacrificed after 7 days. (**D**) Liver tissue samples were analyzed for total glutamine levels. Outliers were removed using Rout and Grubbs's test. Data are mean ± s.d.; no sig. differences were observed by multiple unpaired t-tests. n=14 for NaCl, 12 for MSX. (**E**) Analysis of SREBP2 target gene expression by qPCR from liver tissue samples. Transcript levels were normalized to *Hprt1* levels and are relative to the NaCl control group. n=6; data are mean ± s.d. No significant differences were observed by multiple unpaired t-tests.

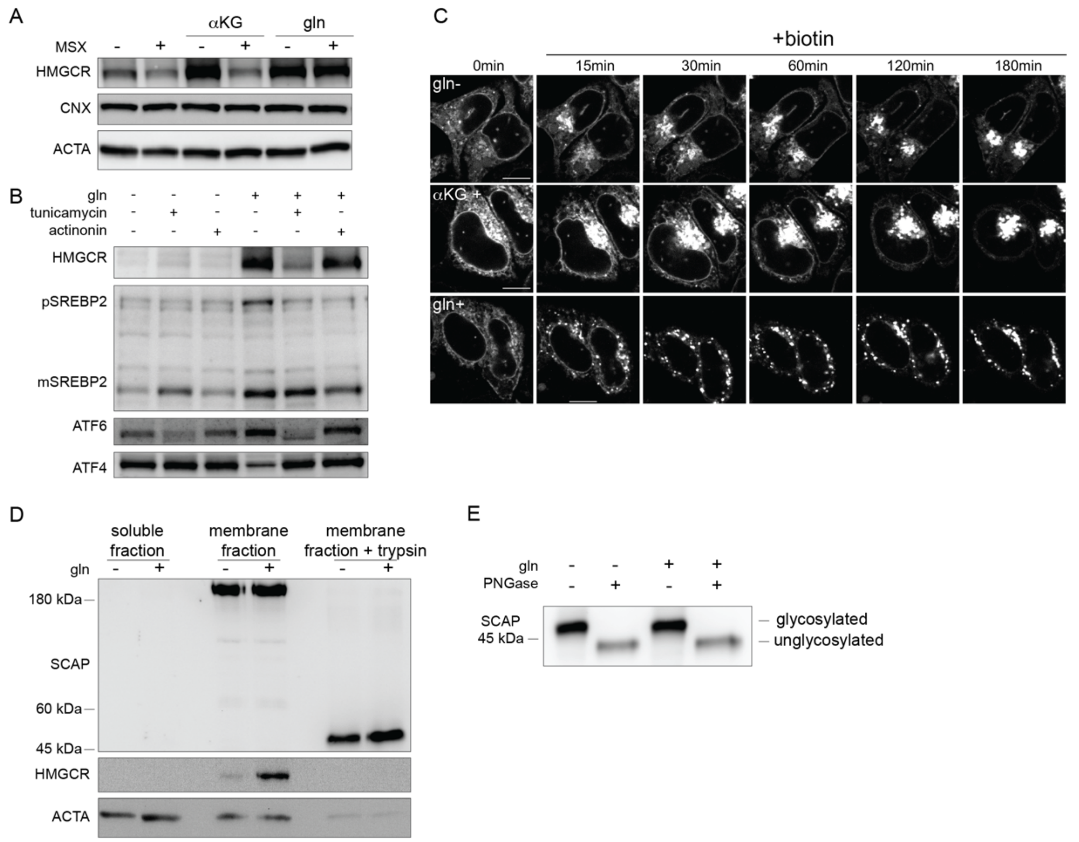

Appendix Figure S5: Glutamine starvation does not impair S1P- and S2P-mediated proteolysis, global ER-to-Golgi trafficking, or SCAP glycosylation (**A**) eGFP-SCAP-expressing CHO cells were starved of gln for 24h, then treated w/gln or w/o gln ± 1 mM αKG ± 500 μM methionine sulfoximine (MSX) for 8h and analyzed by immunoblotting for HMGCR, calnexin (CNX) and actin (ACTA). For all experiments, gln was used at 2 mM. (**B**) U2OS cells were cultured w/o glutamine for 24h and treated as indicated for 8 hours. Concentrations used: 2 mM glutamine (gln), 15 μg/ml tunicamycin, 100μM actinonin. Samples were analyzed by immunoblotting for HMGCR, precursor and mature SREBP2 (pSREBP2, mSREBP2), ATF6, and ATF4. (**C**) HeLas stably expressing mannosidase II-mCherry (MANII) fused to a streptavidin-binding peptide, and streptavidin fused KDEL which retains it in the ER, were cultured without glutamine for 24h. Following 8h of treatment w/ glutamine (gln+) or w/o glutamine ± 1 mM αKG (gln-, αKG+, respectively). Representative live-cell images of mCherry-MANII at indicated time points following 40 μM biotin addition. Scale bar 10 μm. (**D**) Membrane fractionation of eGFP-SCAP-expressing CHO cells cultured in the presence of 500 μM MSX ± 2 mM gln for 24h. Membrane fractions were treated with or w/o 1 μg of trypsin as indicated and analyzed by immunoblotting for SCAP, HMGCR, and ACTA. (**E**) Trypsonized membrane fractions from (D) were treated ± 1U PNGaseF and analyzed by immunoblotting for SCAP.

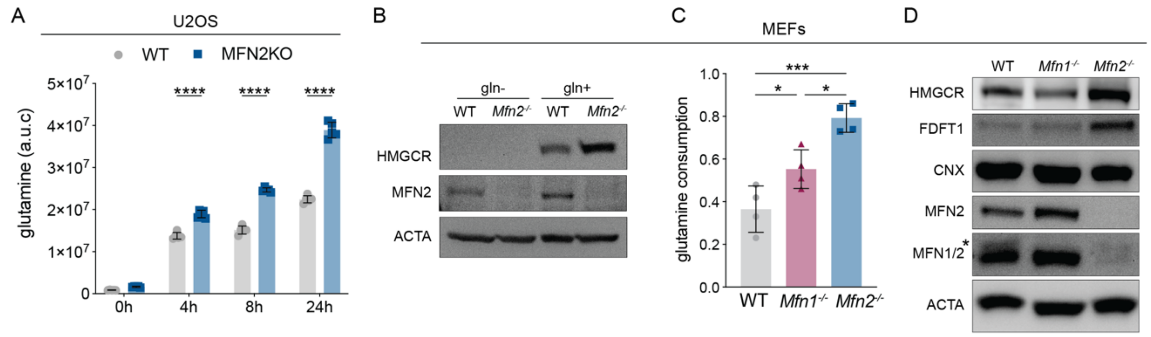

Appendix Figure S6: MFN2 loss drives an increase in HMGCR independently of mitochondrial fusion. (**A**) WT MEFs were cultured without glutamine (gln) for 8h and analyzed by immunoblotting for HMGCR, calnexin (CNX), and actin (ACTA). (**B**) The difference in gln concentration between DMEM and 24h-conditioned media from WT, *Mfn1*$^{-/-}$, *Mfn2*$^{-/-}$ MEFs cultures (gln consumption). Data are mean ± s.d. of n=4 independent cultures; *p<0.05; ***p<0.001 by one-way ANOVA. (**C**) WT, *Mfn1*$^{-/-}$ and *Mfn2*$^{-/-}$ MEFs cultured without lipids for 24h were analyzed by immunoblotting for HMGCR, FDFT1, calnexin (CNX), MFN1 (*denotes MFN1, bottom band MFN2), MFN2, and ACTA. (**D**) WT and MFN2KO U2OS cells were starved of gln for 24h and refed gln and harvested at the indicated time points. Intracellular gln concentration was measured by LS-MS. Data are mean ± s.d. of n=5 independent cultures; ****p<0.0001 by two-way ANOVA.
