# Supplementary material for: Glutamine sensing licenses cholesterol synthesis
Source: EMBO J. 2024 Oct 21;43(23):5837–56. doi: 10.1038/s44318-024-00269-0 (PMC11612431; doi:10.1038/s44318-024-00269-0)
Supplement: Supplementary file 2 — Table EV1 [file 44318_2024_269_MOESM2_ESM.docx]

**Table EV1. Composition of the purified Glutamine adjusted diets**

Dietary composition of 1.8% glutamine diet (control) and iso-nitrogenous to control diet without glutamine (glutamine-free) from ssniff

| **Ingredients** | U | **w/o Glutamine** | **1.8 % Glutamine** |
| --- | --- | --- | --- |
|  |  | **Iso-N** | **Control** |
| *Product No.* |  | *S9159-E760* | *S9159-E764* |
| Corn starch | % | 20.000 | 20.000 |
| Maltodextrin | % | 16.000 | 16.000 |
| Sucrose | % | 27.850 | 28.000 |
| Cellulose powder | % | 5.000 | 5.000 |
| Chicory inulin | % | 1.000 | 1.000 |
| L-Lysine HCl | % | 1.800 | 1.800 |
| DL-Methionine | % | 0.710 | 0.710 |
| L-Cystine | % | 0.350 | 0.350 |
| L-Threonine | % | 0.850 | 0.850 |
| L-Tryptophan | % | 0.180 | 0.180 |
| L-Arginine, free base | % | 1.000 | 1.000 |
| L-Histidine | % | 0.450 | 0.450 |
| L-Valine | % | 0.850 | 0.850 |
| L-Isoleucine | % | 0.820 | 0.820 |
| L-Leucine | % | 1.150 | 1.150 |
| L-Phenylalanine | % | 0.750 | 0.750 |
| L-Tyrosine | % | 0.500 | 0.500 |
| **Glycine** | **%** | **3.650** | **2.350** |
| **L-Glutamic acid** | **%** | **2.200** | **2.200** |
| **L-Glutamine** | **%** | **——** | **1.810** |
| L-Aspartic acid | % | 0.350 | 0.350 |
| L-Asparagine | % | 0.600 | 0.600 |
| L-Proline | % | 0.350 | 0.350 |
| L-Serine | % | 0.350 | 0.350 |
| **L- Alanine** | **%** | **1.010** | **0.350** |
| Vitamin premix | % | 1.000 | 1.000 |
| Minerals/Trace minerals | % | 4.000 | 4.000 |
| Choline Cl (50 %) | % | 0.200 | 0.200 |
| Dye [Yellow / Green] | % | 0.030 | 0.030 |
| Soybean oil | % | 7.000 | 7.000 |

**Table 1. *continued***

| **Nutrients** | U | **w/o Glutamine** | **1.8 % Glutamine** |
| --- | --- | --- | --- |
|  |  | **Iso-N** | **Control** |
| *Product No.* |  | *S9159-E760* | *S9159-E764* |
| ***Proximate contents*** |  |  |  |
| Crude protein ^1)^ | % | 16.5 | 16.5 |
| Crude fat | % | 7.0 | 7.0 |
| Crude fibre | % | 5.9 | 5.9 |
| Crude ash | % | 3.6 | 3.6 |
| Starch | % | 19.3 | 19.3 |
| Dextrin | % | 15.8 | 15.8 |
| Sugar | % | 28.8 | 28.9 |
| Lysine | % | 1.40 | 1.40 |
| Methionine | % | 0.70 | 0.70 |
| Cystine | % | 0.35 | 0.35 |
| Threonine | % | 0.80 | 0.80 |
| Tryptophan | % | 0.18 | 0.18 |
| **Glycine** | **%** | **3.61** | **2.33** |
| **Glutamic acid** | **%** | **2.18** | **2.18** |
| **Glutamine** | **%** | **——** | **1.80** |
| **Alanine** | **%** | **1.00** | **0.35** |
| Energy (Atwater) ^2)^ | MJ/kg | 16.5 | 16.5 |
| Protein | kJ% | 17 | 17 |
| Fat | kJ% | 16 | 16 |
| Carbohydrates | kJ% | 67 | 67 |

*^1)^ Calculated from the amino acids (N x 6.25)*

*^2)^ = Physiological fuel value*
