# Supplementary figures and images for: Glutamine sensing licenses cholesterol synthesis
Source: EMBO J. 2024 Oct 21;43(23):5837–56. doi: 10.1038/s44318-024-00269-0 (PMC11612431; doi:10.1038/s44318-024-00269-0)

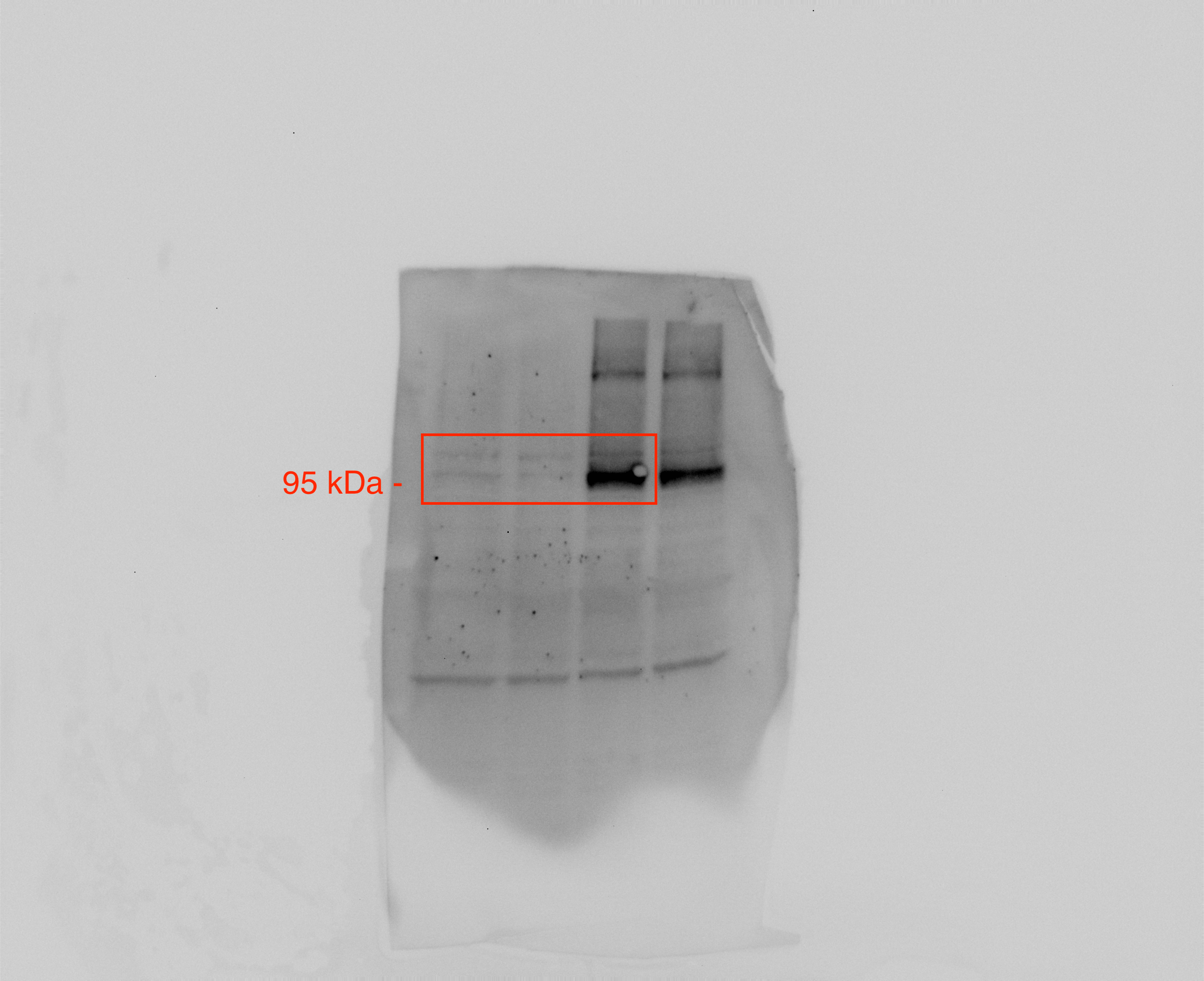

Supplement: Supplementary file 6 — Source data Fig. 2 [file 44318_2024_269_MOESM6_ESM.zip › Figure 2/2I/HMGCR.tif]

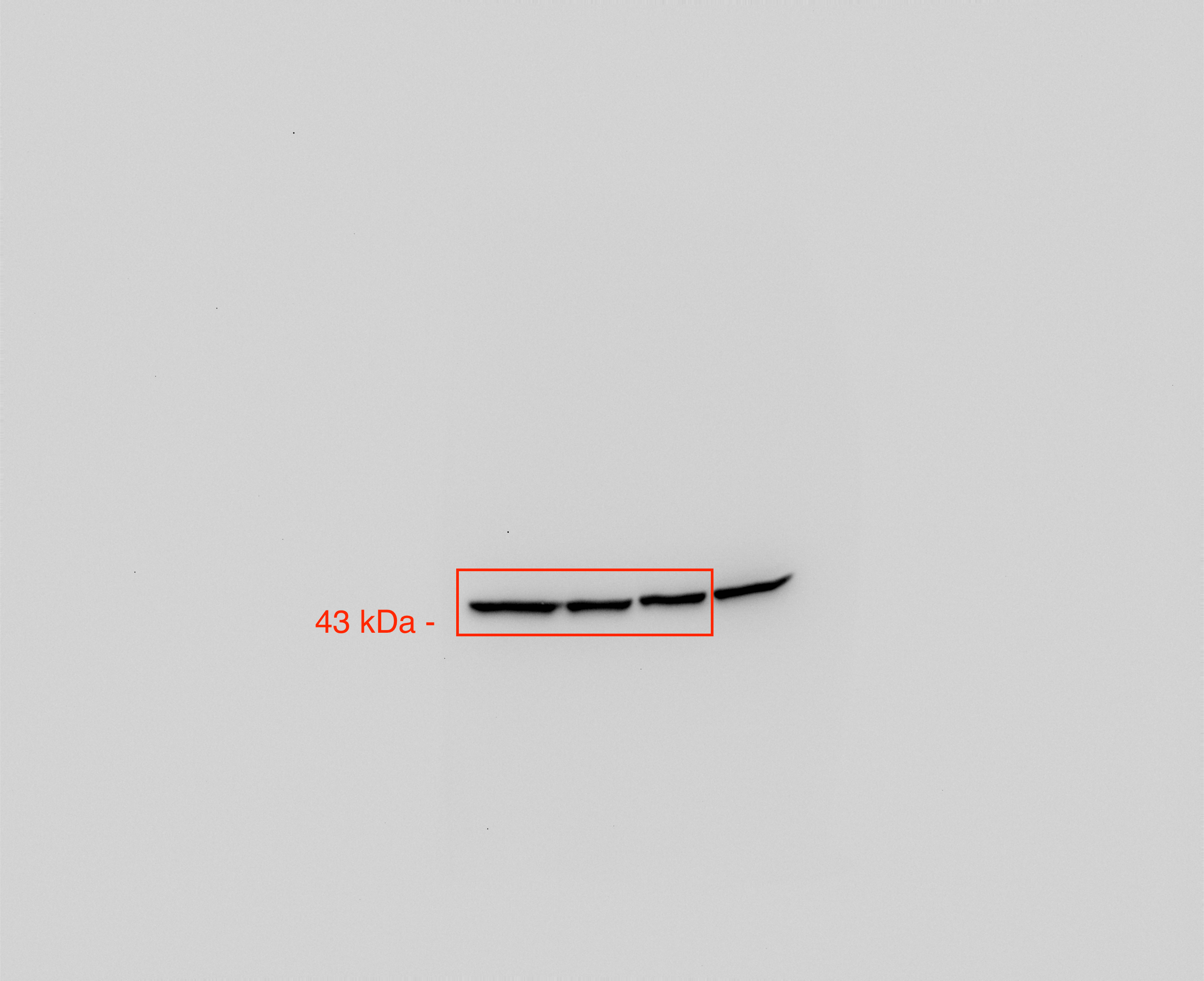

Supplement: Supplementary file 6 — Source data Fig. 2 [file 44318_2024_269_MOESM6_ESM.zip › Figure 2/2I/ACTIN.tif]

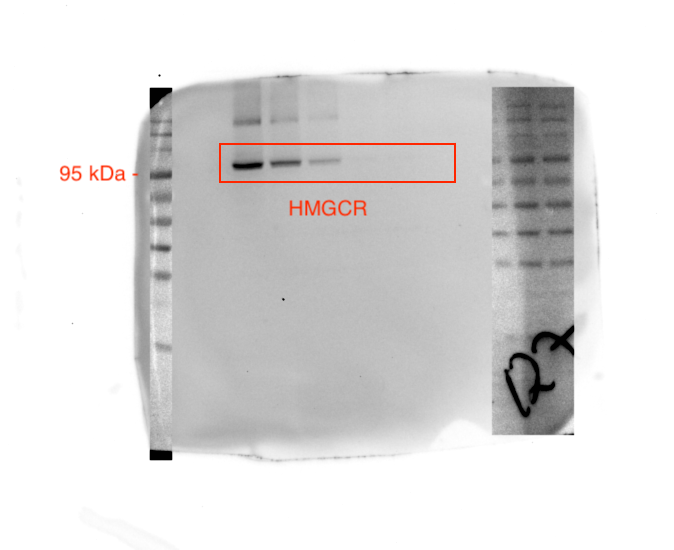

Supplement: Supplementary file 6 — Source data Fig. 2 [file 44318_2024_269_MOESM6_ESM.zip › Figure 2/2A/HMGCR.png]

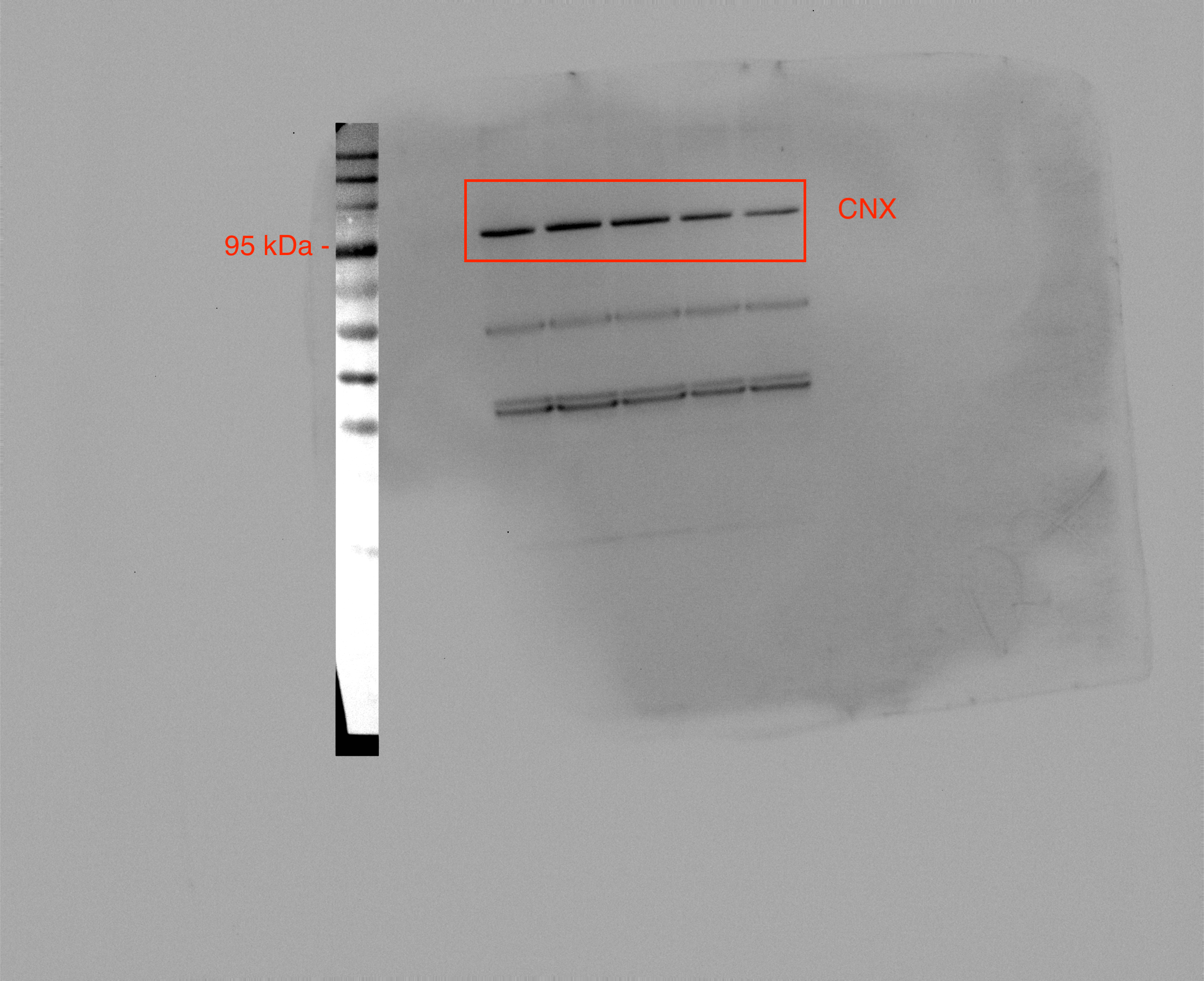

Supplement: Supplementary file 6 — Source data Fig. 2 [file 44318_2024_269_MOESM6_ESM.zip › Figure 2/2A/CNX.png]

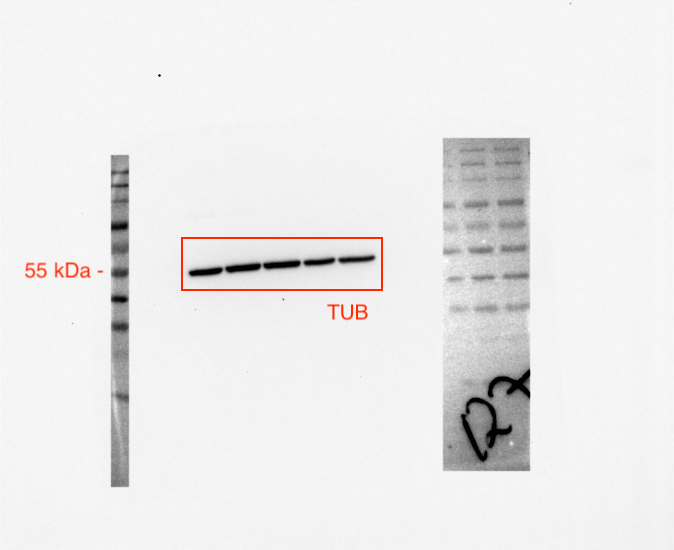

Supplement: Supplementary file 6 — Source data Fig. 2 [file 44318_2024_269_MOESM6_ESM.zip › Figure 2/2A/TUBA.png]

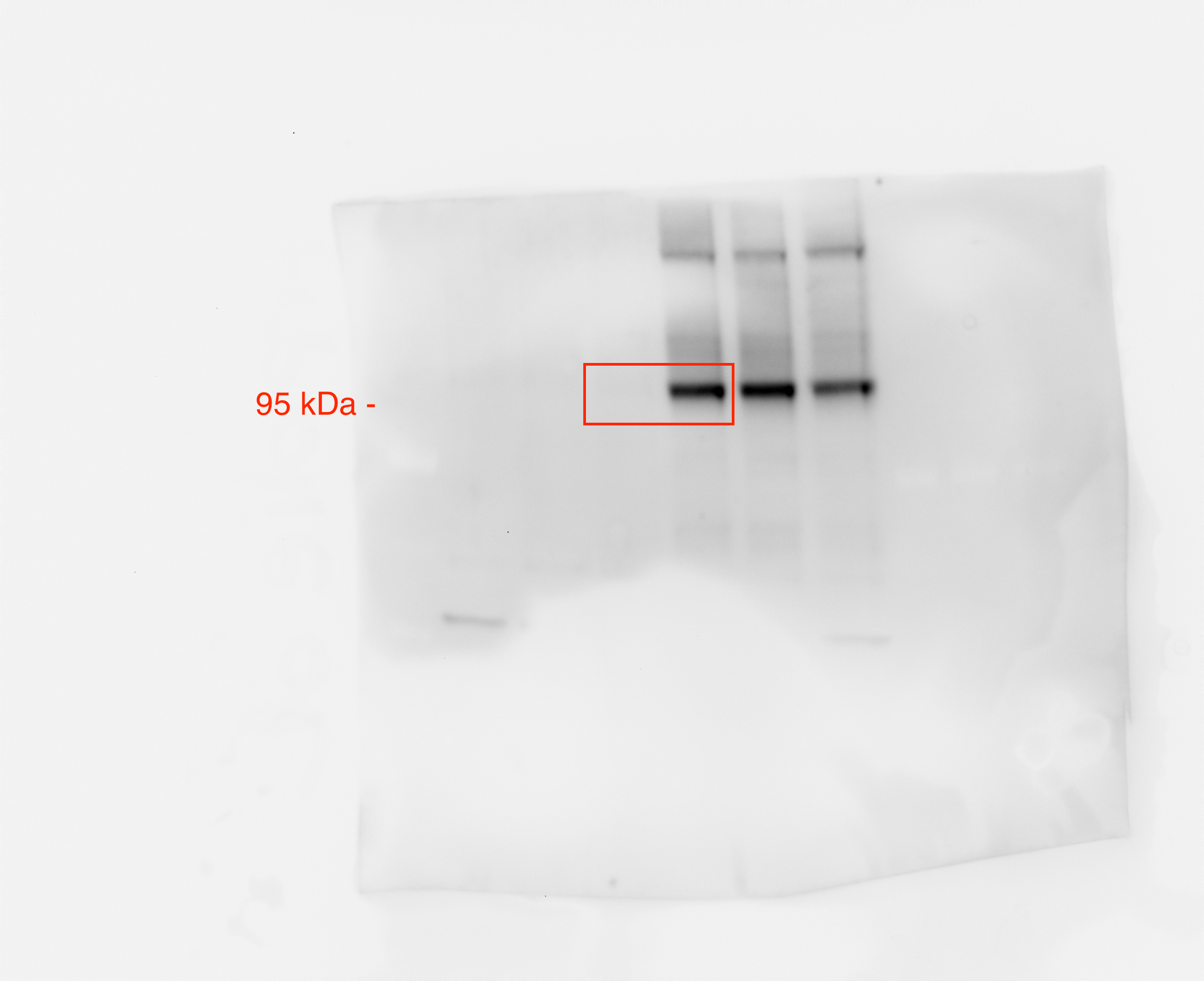

Supplement: Supplementary file 6 — Source data Fig. 2 [file 44318_2024_269_MOESM6_ESM.zip › Figure 2/2C/HMGCR.tiff]

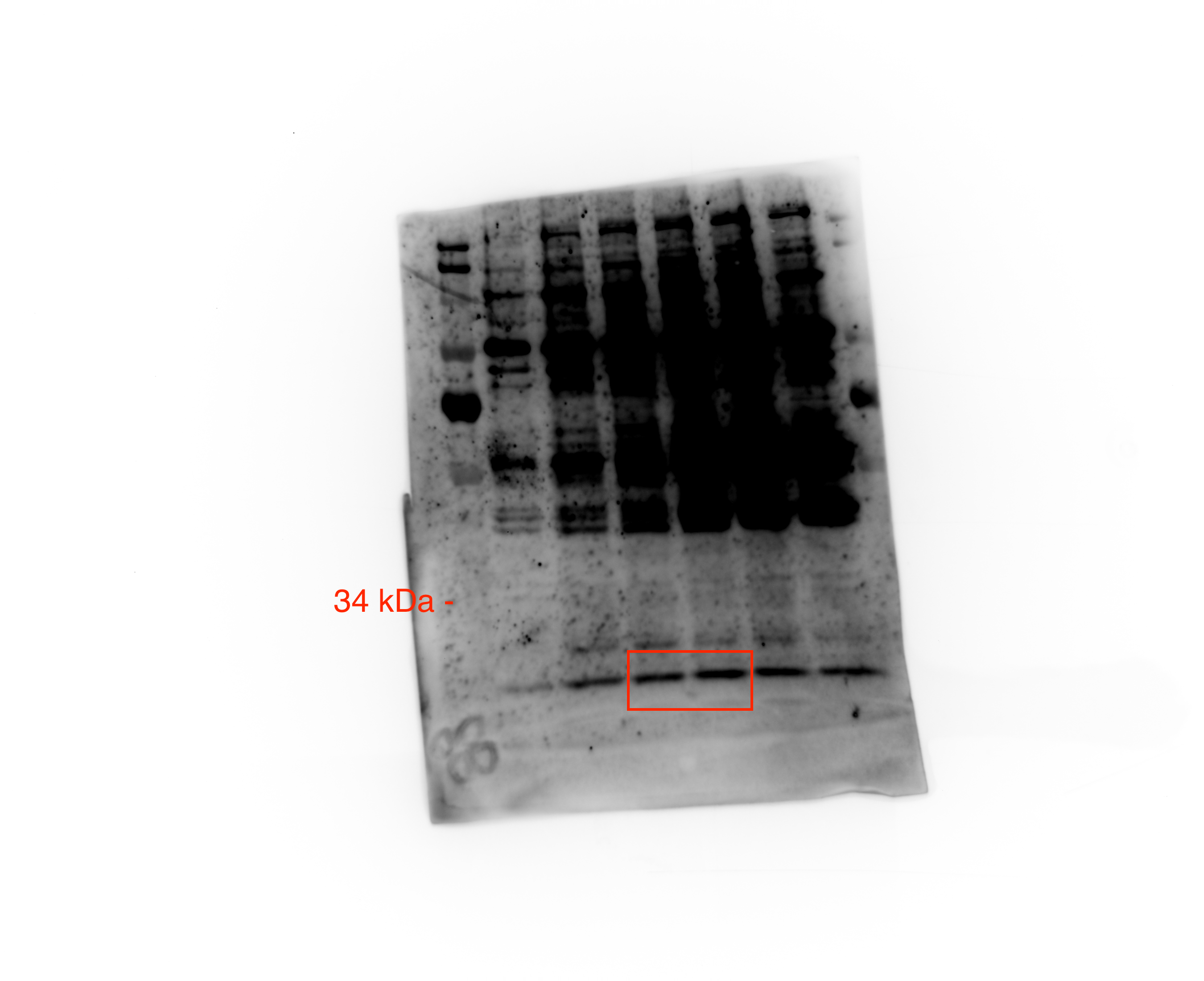

Supplement: Supplementary file 6 — Source data Fig. 2 [file 44318_2024_269_MOESM6_ESM.zip › Figure 2/2C/HPRT1.tiff]

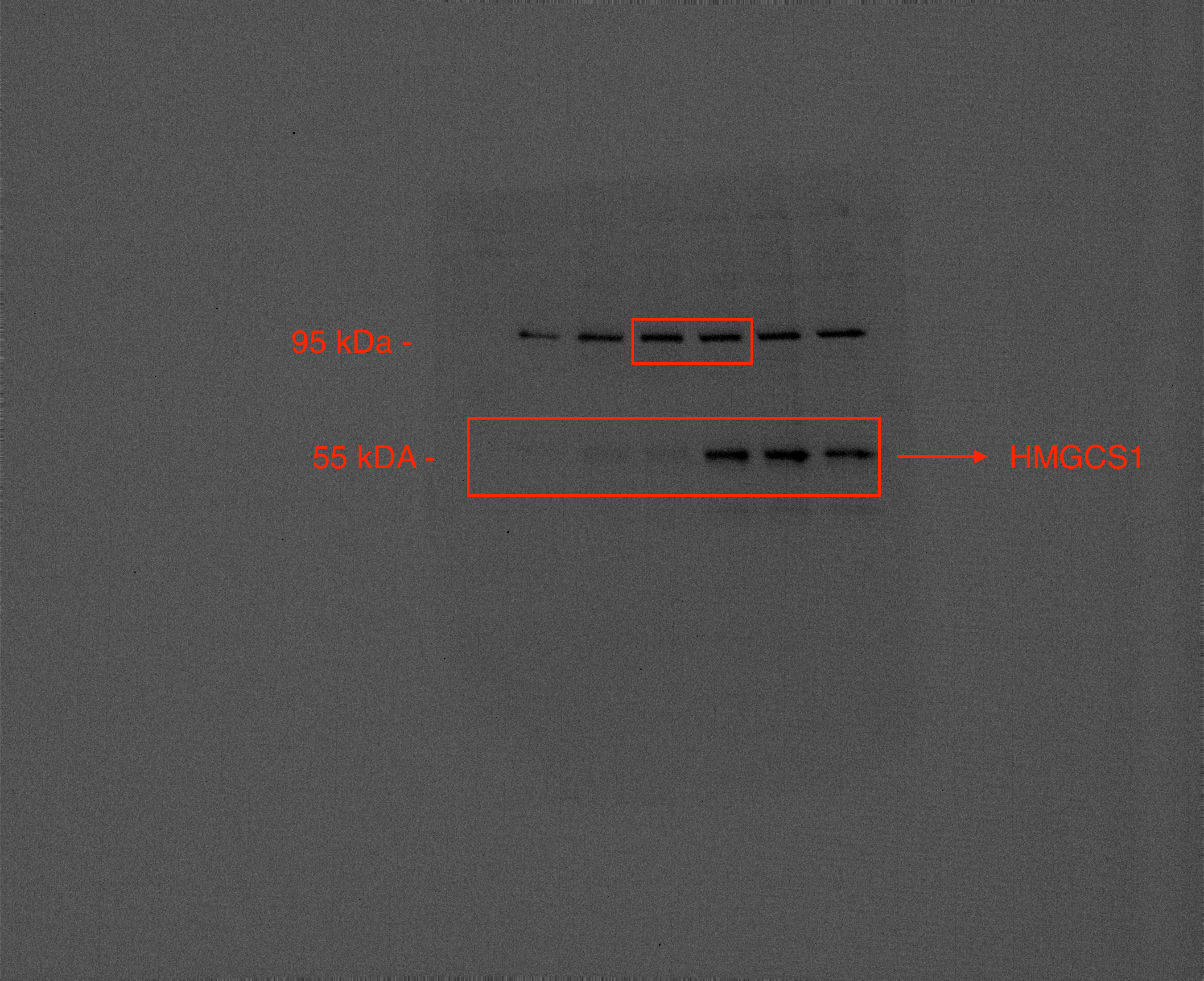

Supplement: Supplementary file 6 — Source data Fig. 2 [file 44318_2024_269_MOESM6_ESM.zip › Figure 2/2C/CNX.tiff]

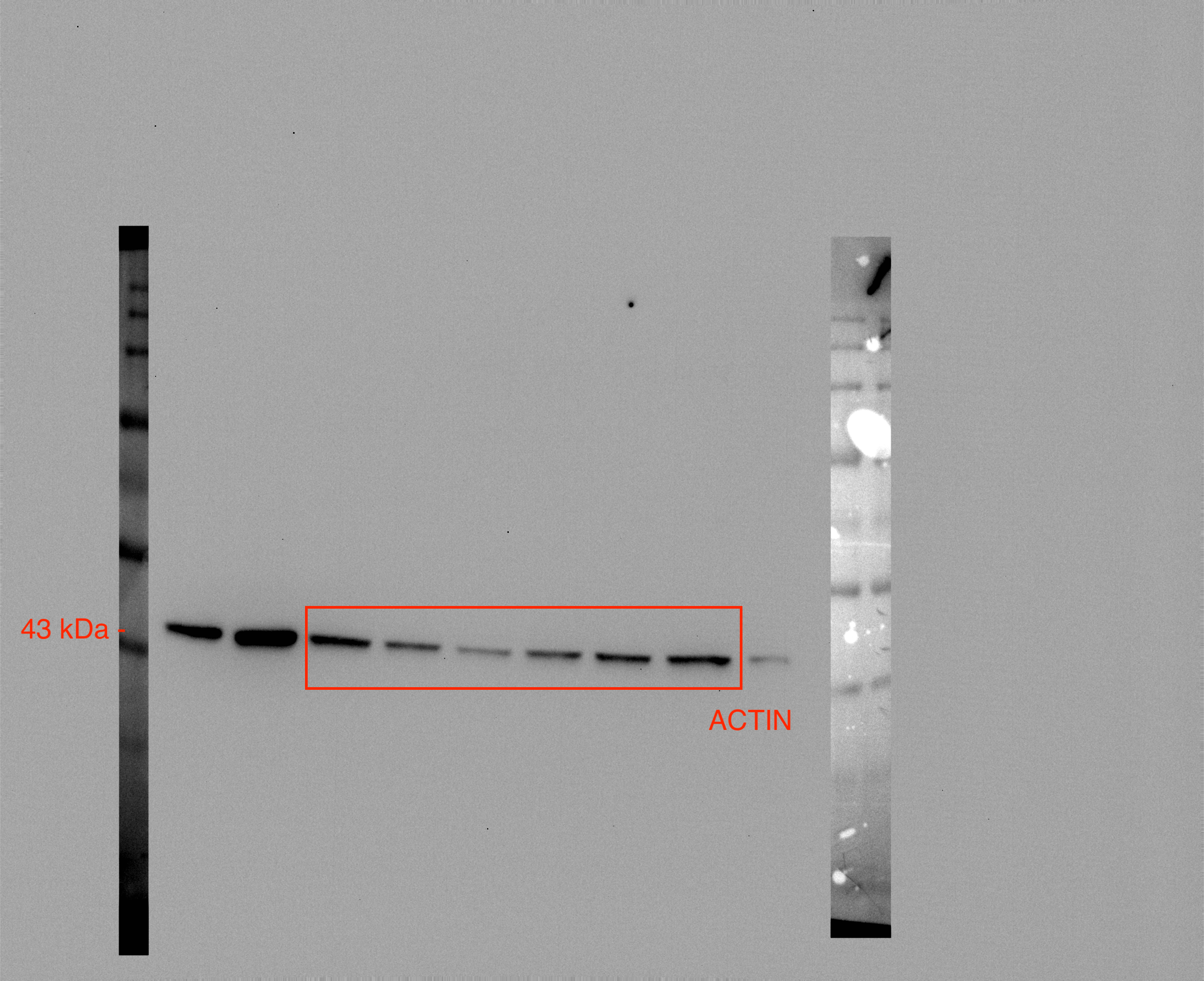

Supplement: Supplementary file 6 — Source data Fig. 2 [file 44318_2024_269_MOESM6_ESM.zip › Figure 2/2M/ACTIN.png]

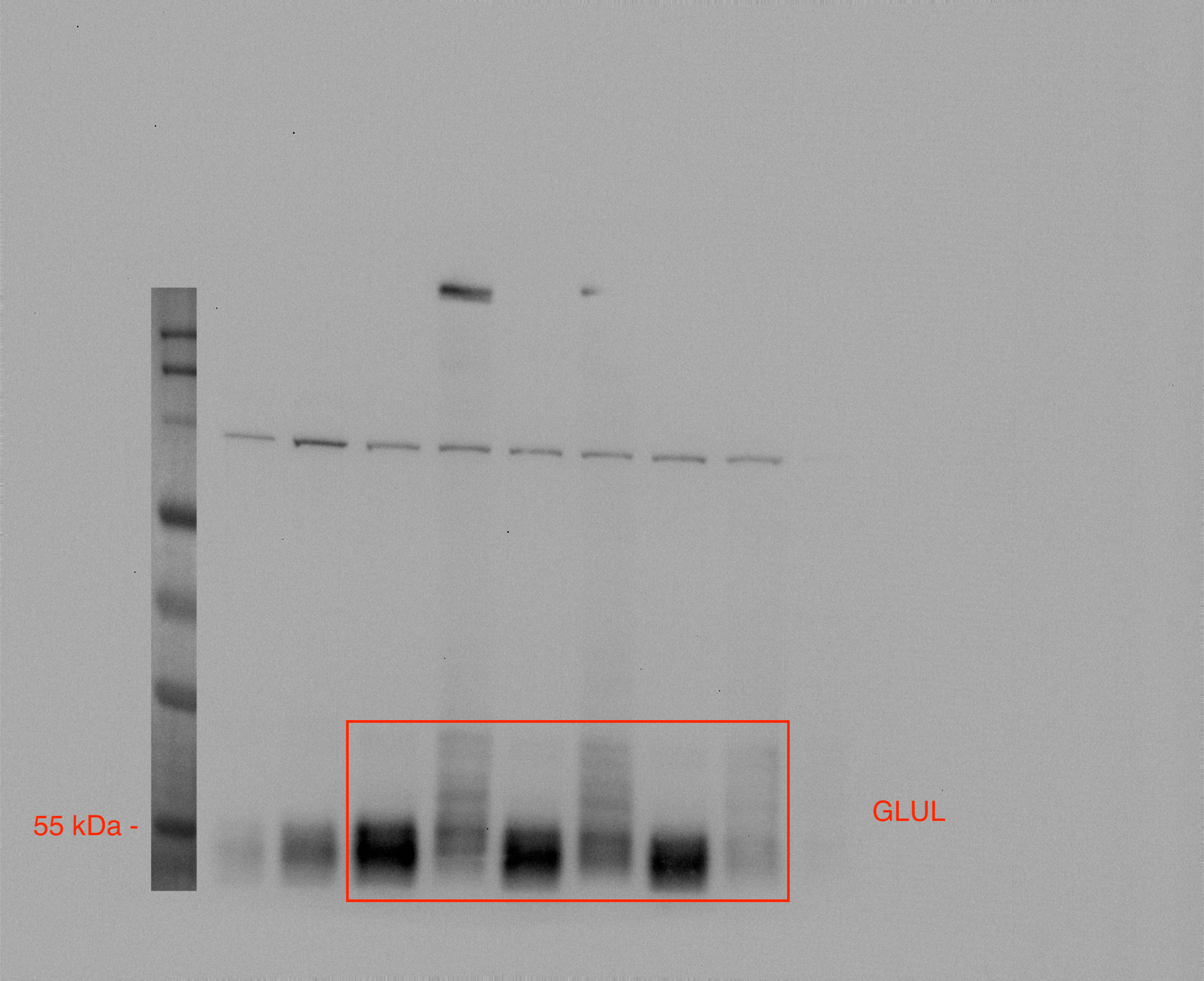

Supplement: Supplementary file 6 — Source data Fig. 2 [file 44318_2024_269_MOESM6_ESM.zip › Figure 2/2M/GLUL.png]

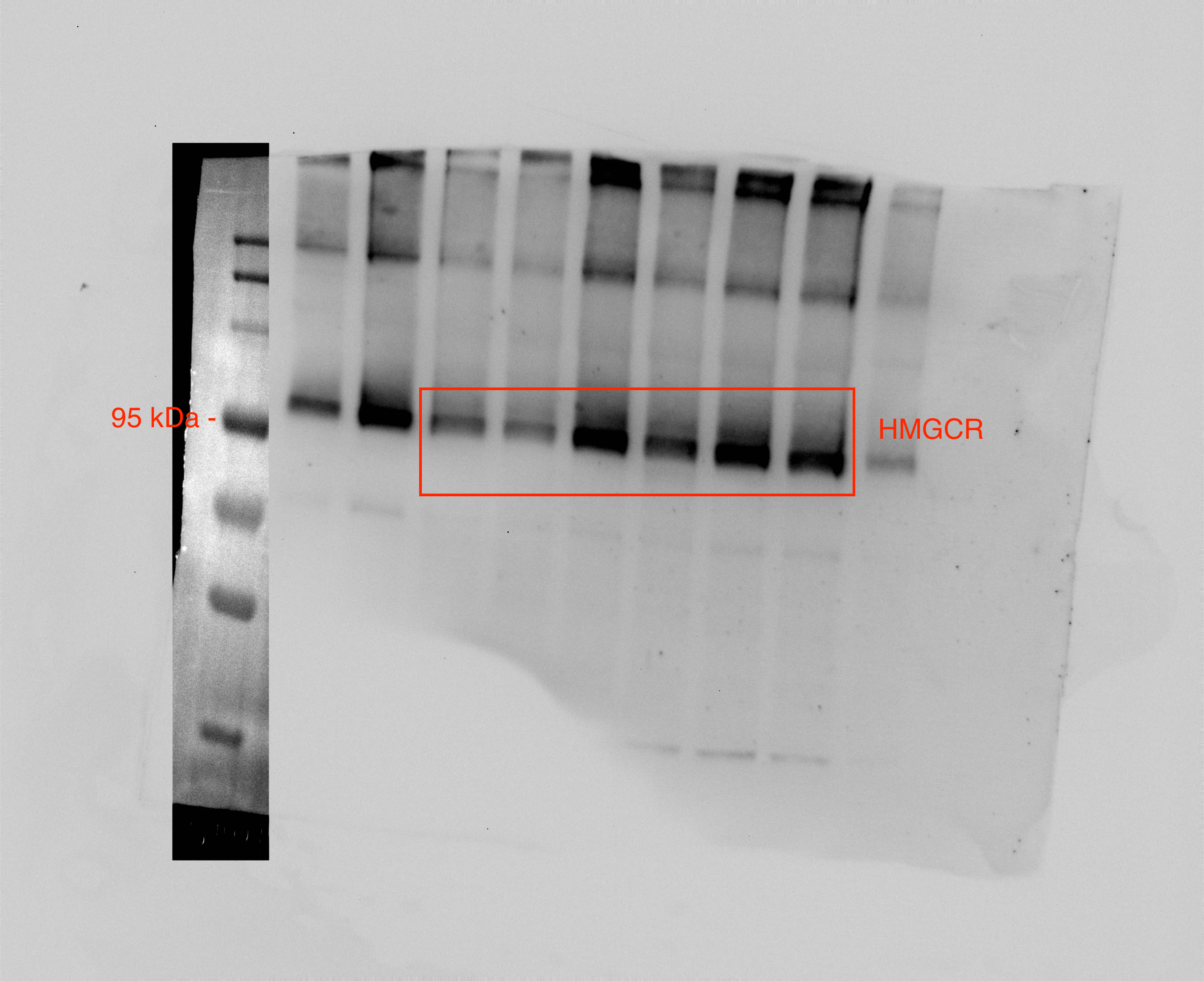

Supplement: Supplementary file 6 — Source data Fig. 2 [file 44318_2024_269_MOESM6_ESM.zip › Figure 2/2M/HMGCR.png]

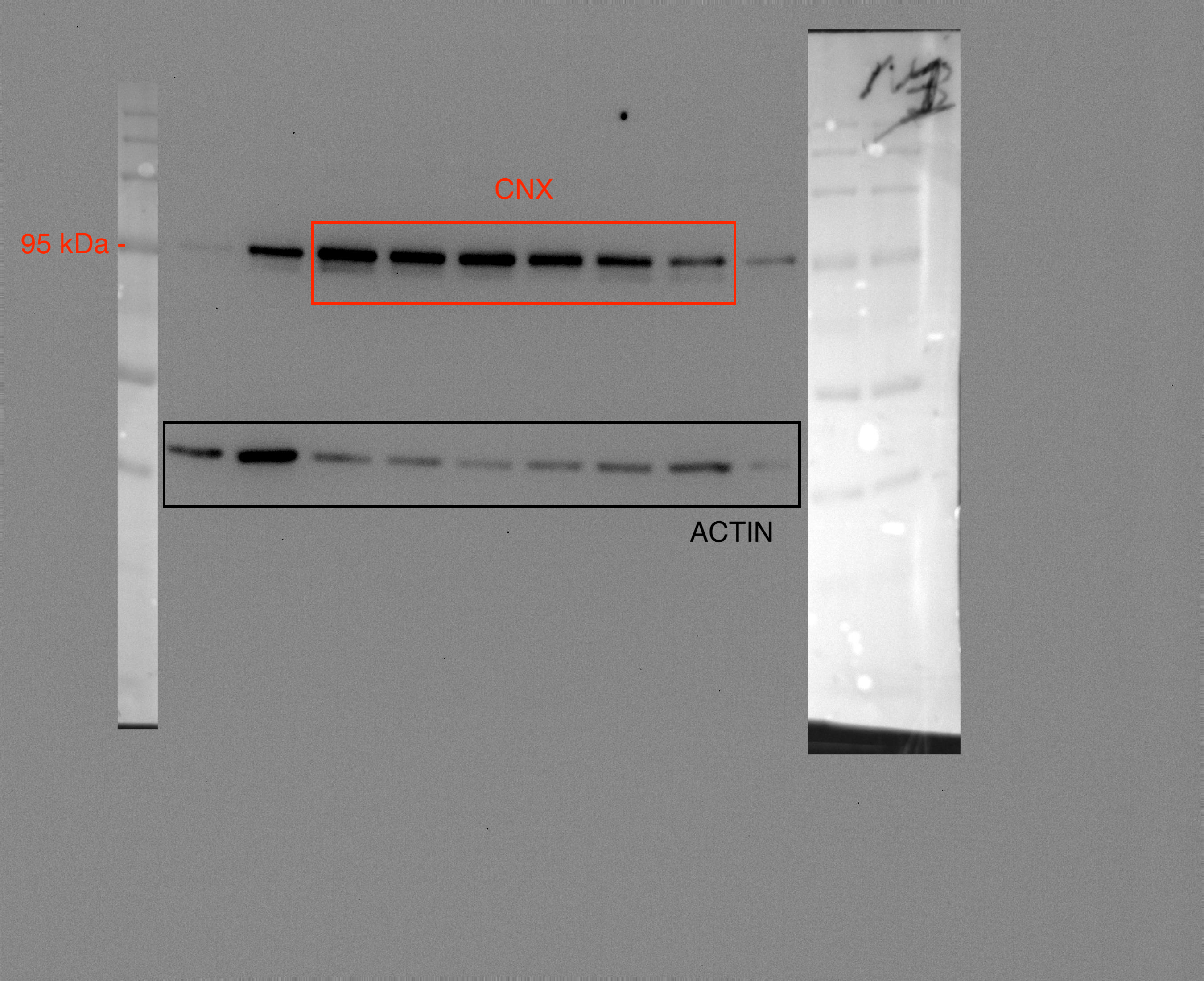

Supplement: Supplementary file 6 — Source data Fig. 2 [file 44318_2024_269_MOESM6_ESM.zip › Figure 2/2M/CNX.png]

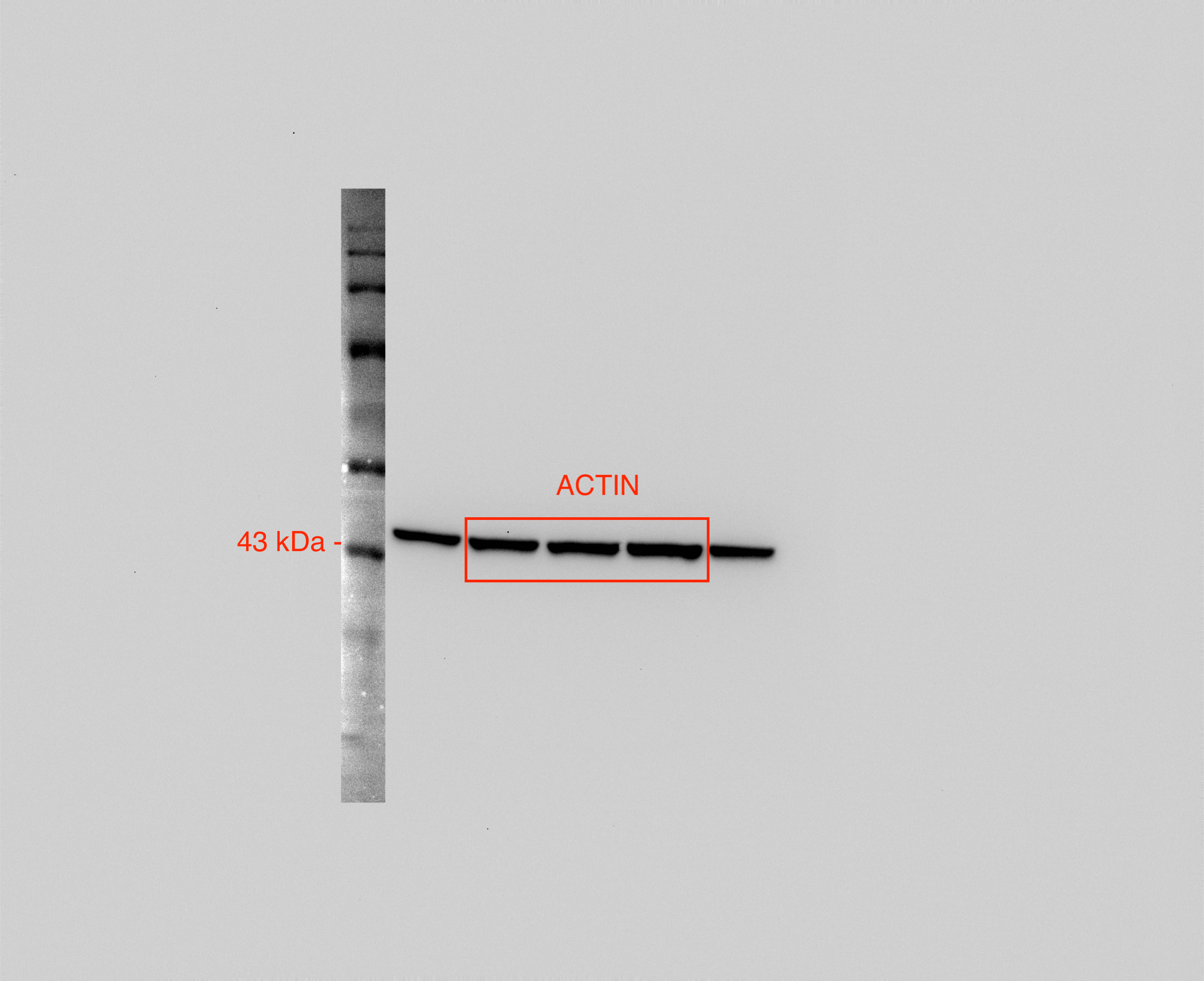

Supplement: Supplementary file 6 — Source data Fig. 2 [file 44318_2024_269_MOESM6_ESM.zip › Figure 2/2J/ACTIN.png]

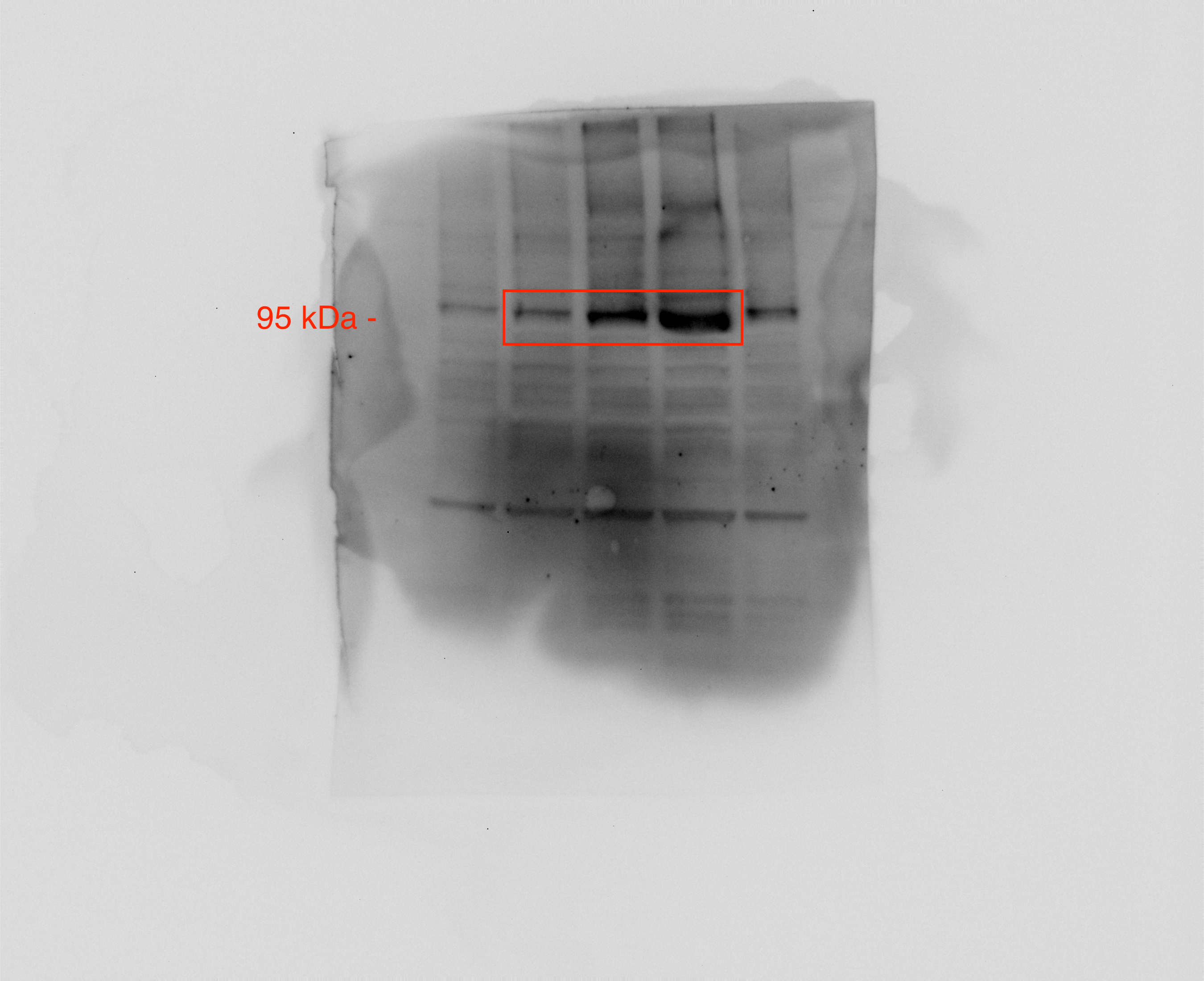

Supplement: Supplementary file 6 — Source data Fig. 2 [file 44318_2024_269_MOESM6_ESM.zip › Figure 2/2J/HMGCR.tif]

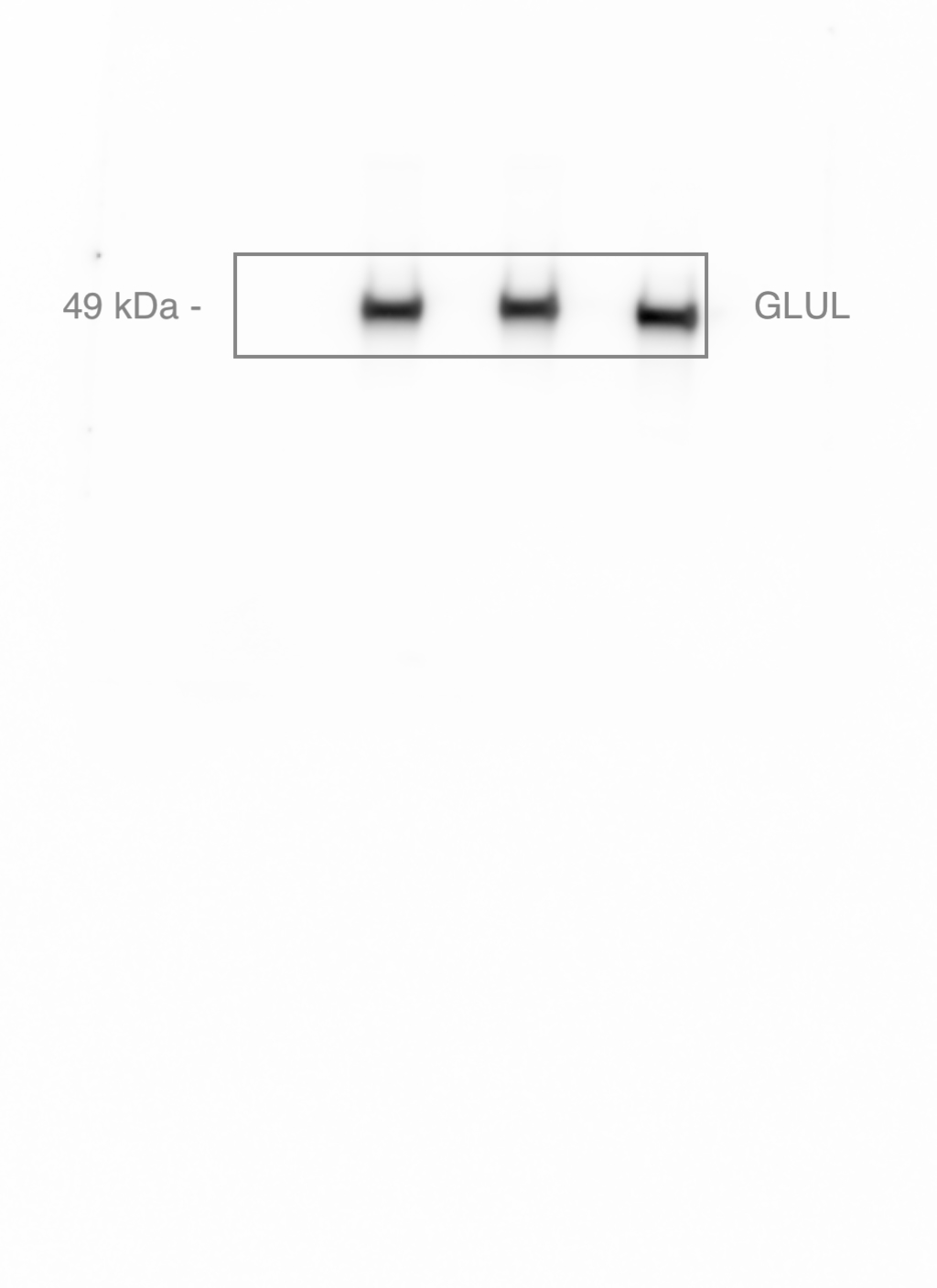

Supplement: Supplementary file 6 — Source data Fig. 2 [file 44318_2024_269_MOESM6_ESM.zip › Figure 2/2K/GLUL.jpg]

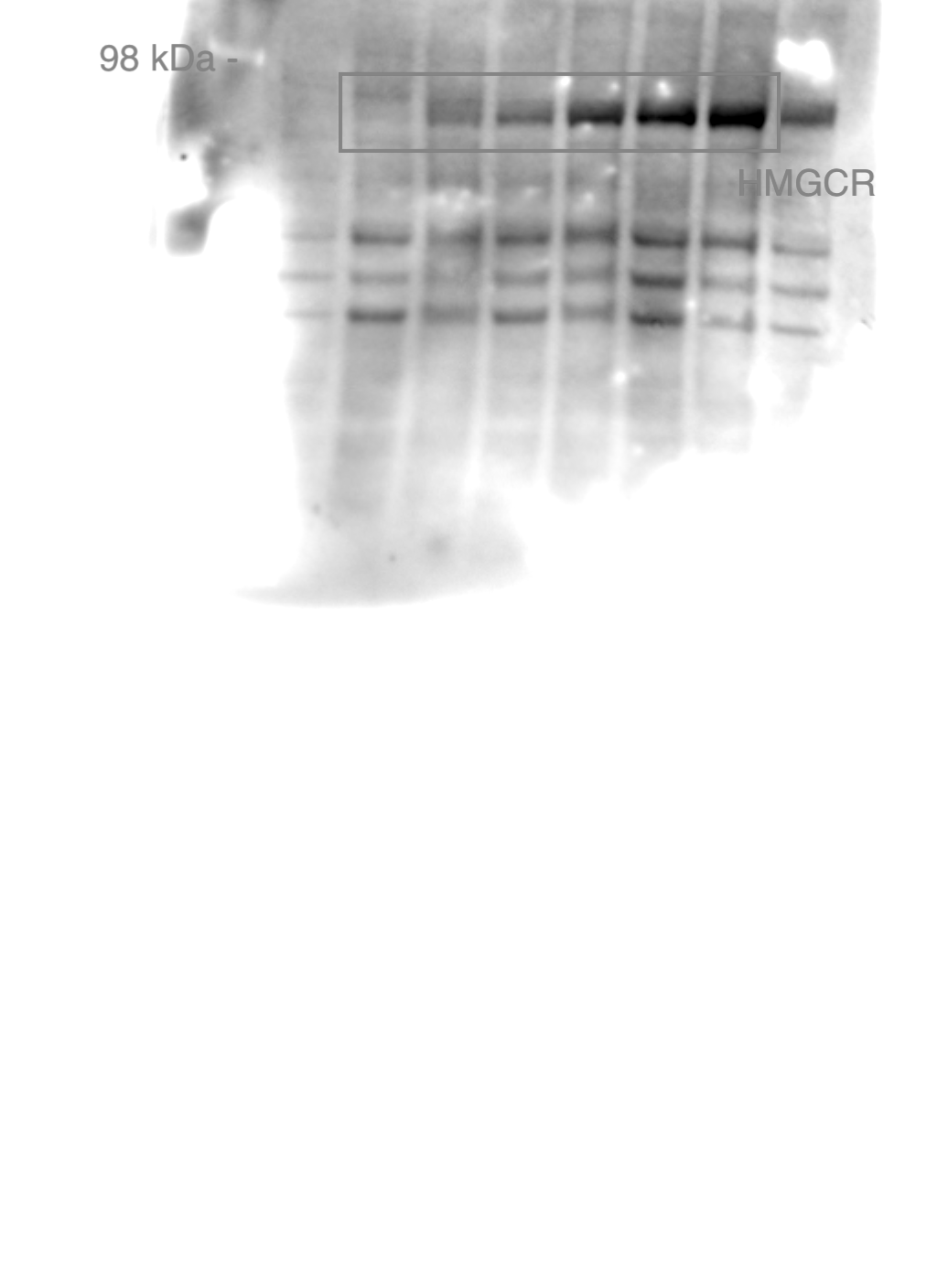

Supplement: Supplementary file 6 — Source data Fig. 2 [file 44318_2024_269_MOESM6_ESM.zip › Figure 2/2K/HMGCR.png]

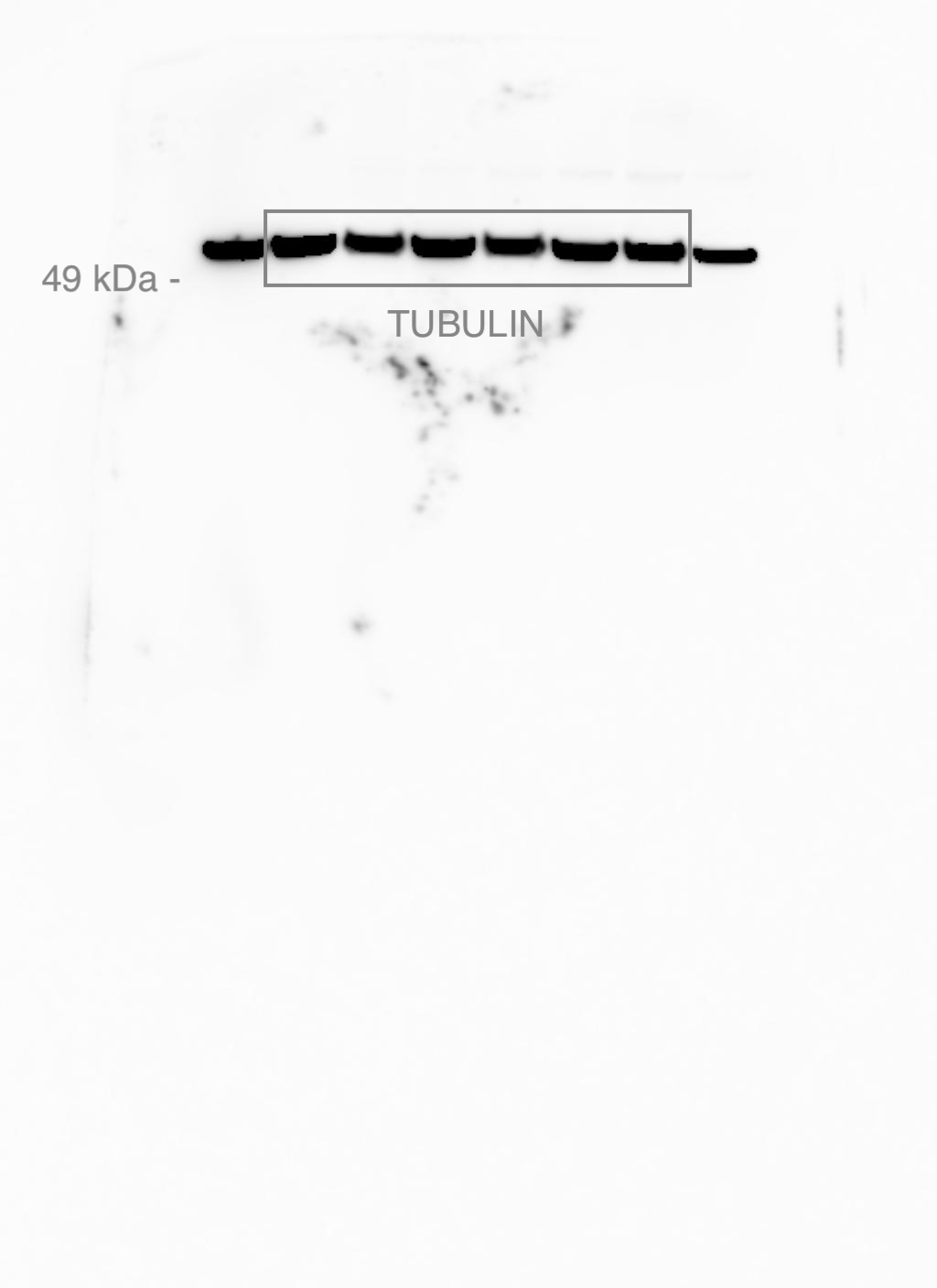

Supplement: Supplementary file 6 — Source data Fig. 2 [file 44318_2024_269_MOESM6_ESM.zip › Figure 2/2K/TUBA.jpg]

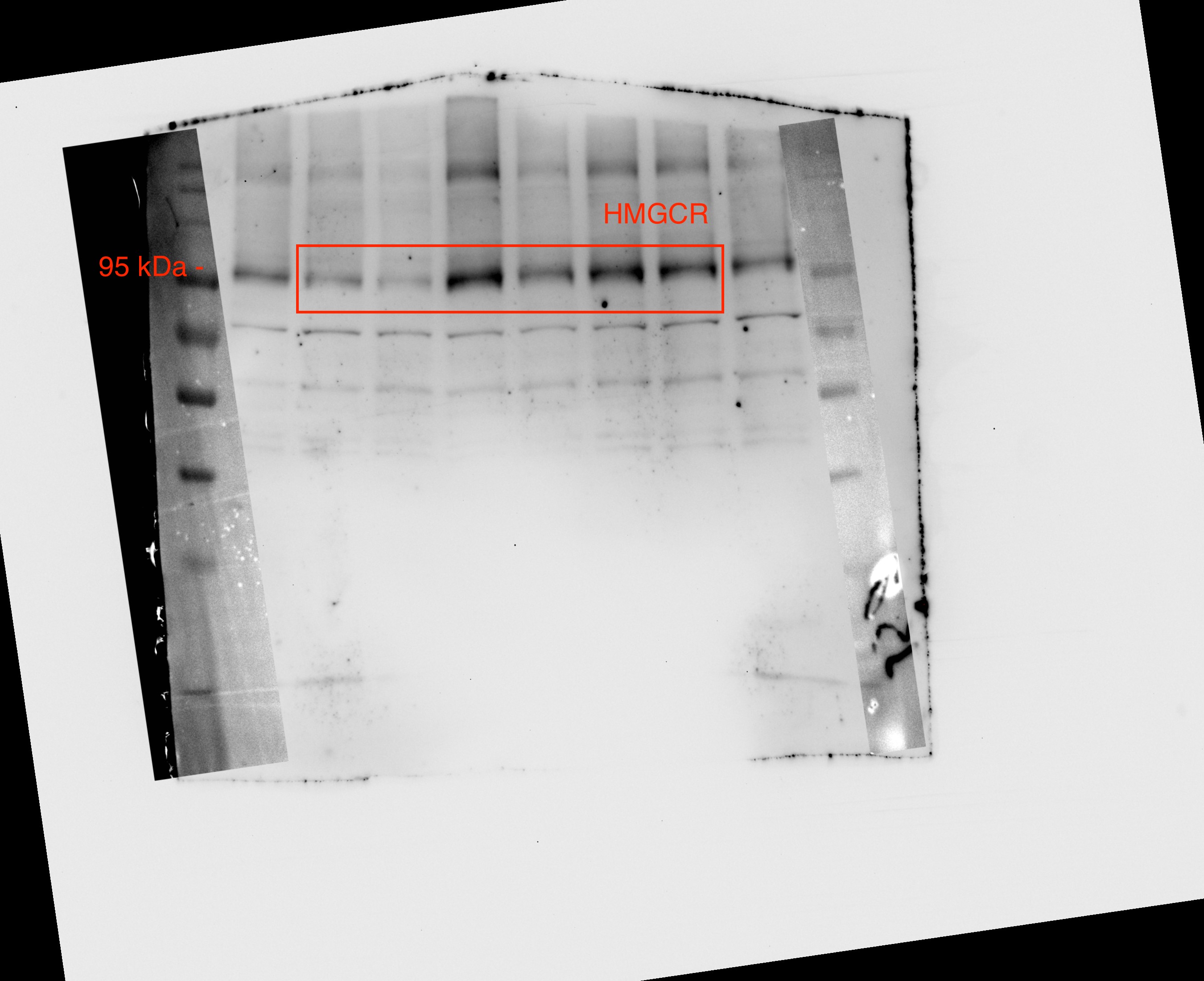

Supplement: Supplementary file 6 — Source data Fig. 2 [file 44318_2024_269_MOESM6_ESM.zip › Figure 2/2L/hmgcr.tif]

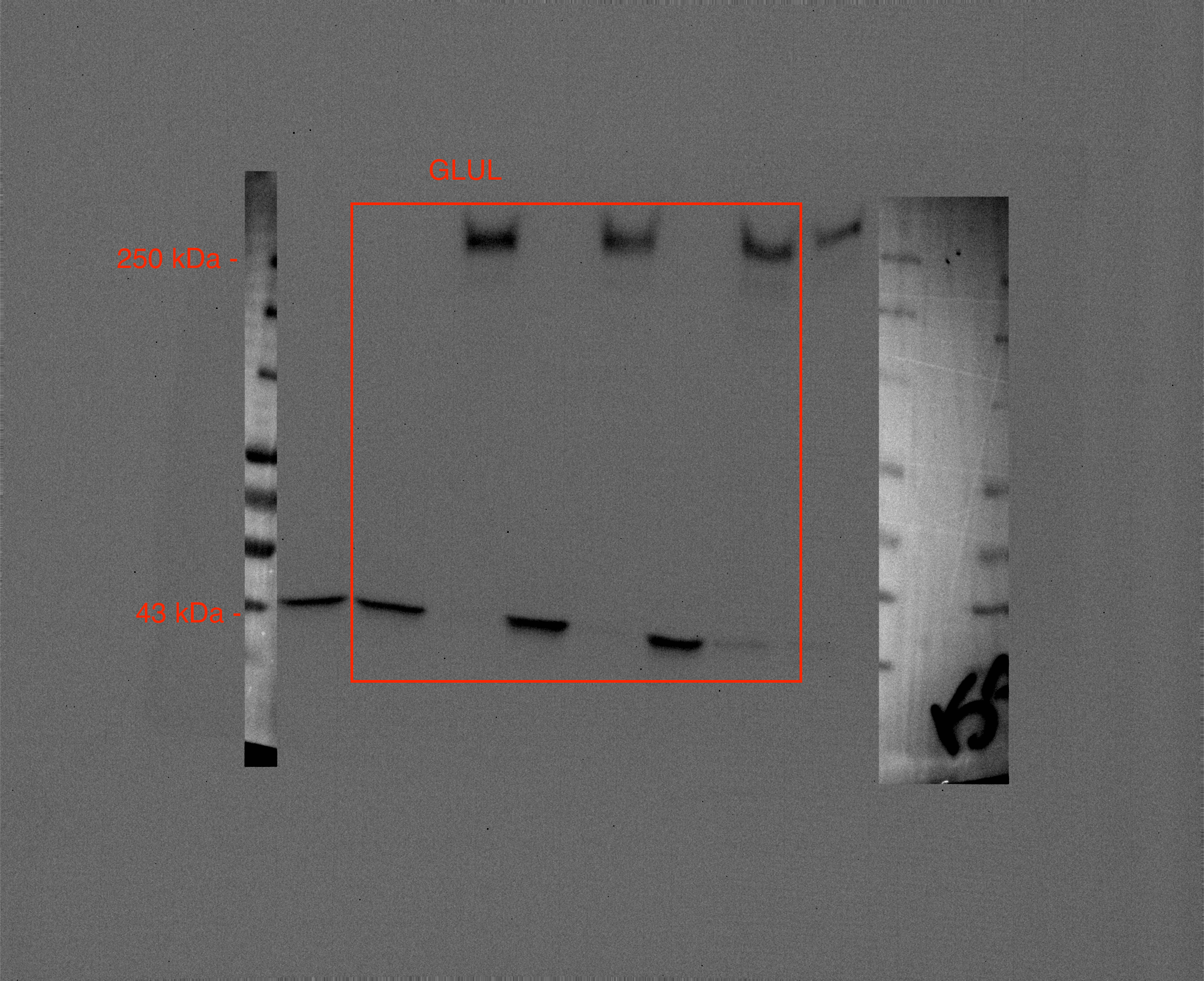

Supplement: Supplementary file 6 — Source data Fig. 2 [file 44318_2024_269_MOESM6_ESM.zip › Figure 2/2L/GLUL.png]

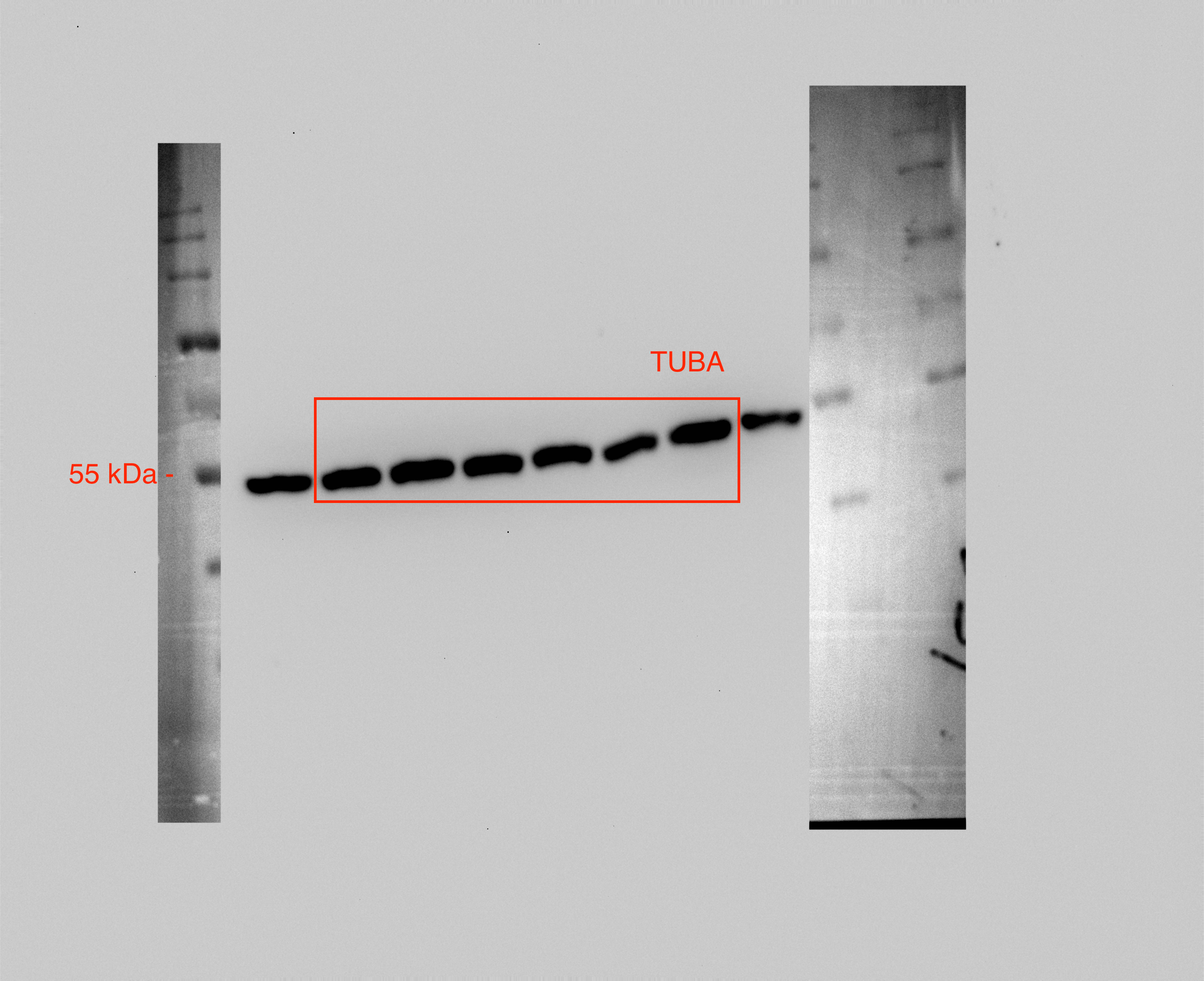

Supplement: Supplementary file 6 — Source data Fig. 2 [file 44318_2024_269_MOESM6_ESM.zip › Figure 2/2L/TUBA.png]

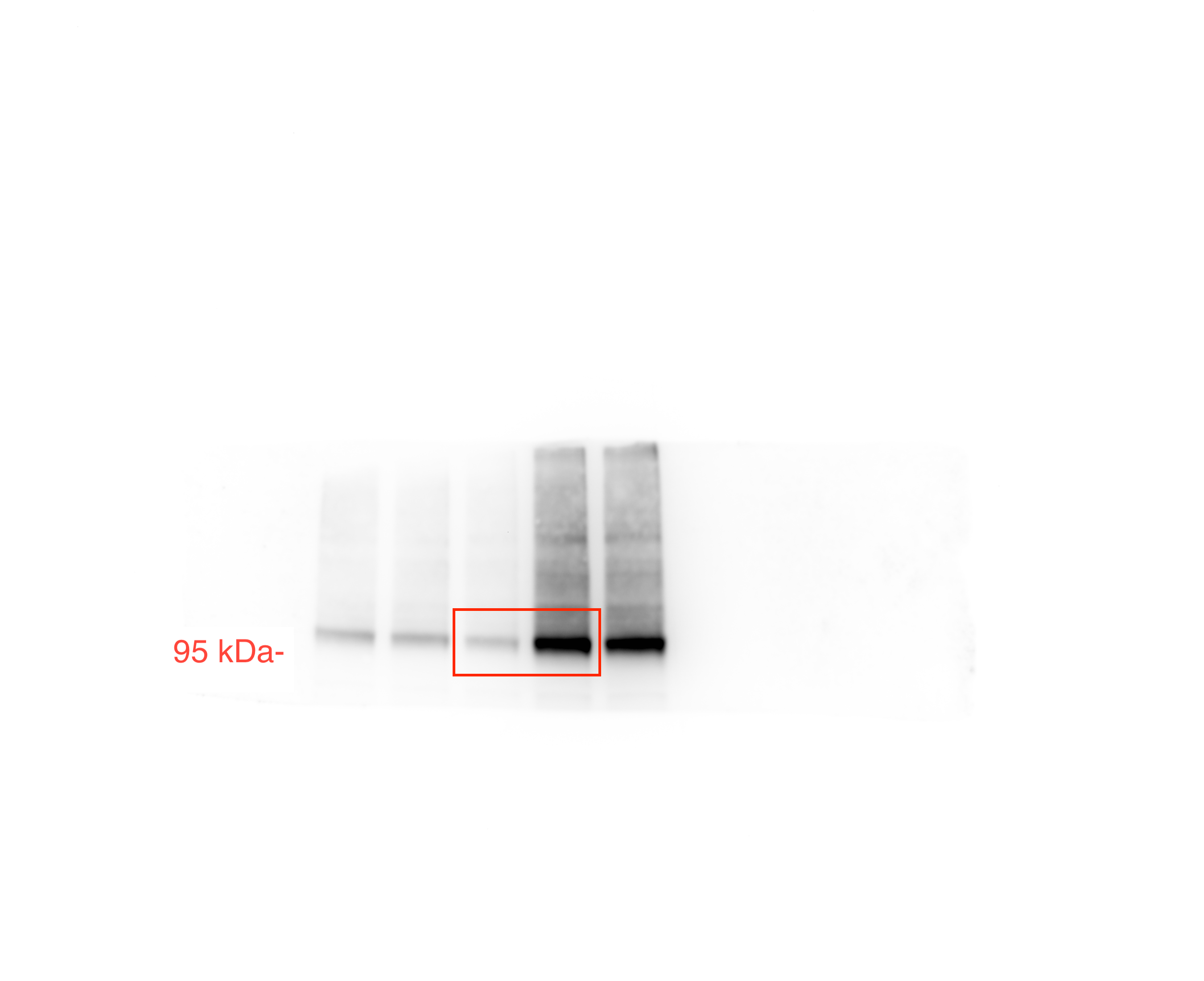

Supplement: Supplementary file 6 — Source data Fig. 2 [file 44318_2024_269_MOESM6_ESM.zip › Figure 2/2B/HMGCR.tif]

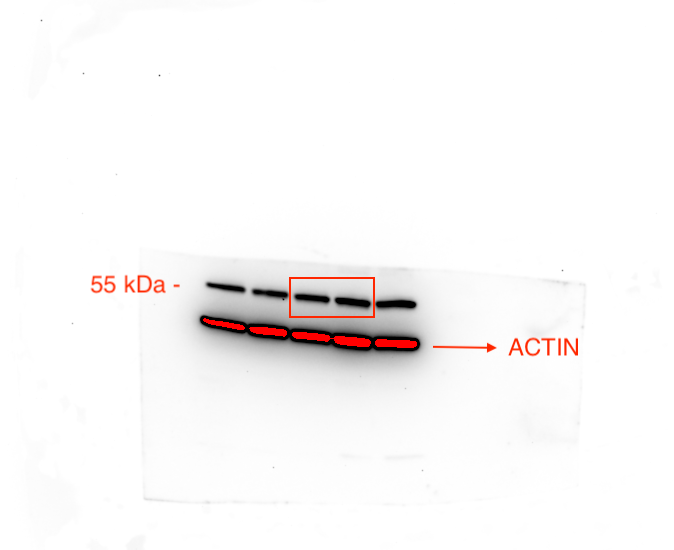

Supplement: Supplementary file 6 — Source data Fig. 2 [file 44318_2024_269_MOESM6_ESM.zip › Figure 2/2B/CALR.tif]

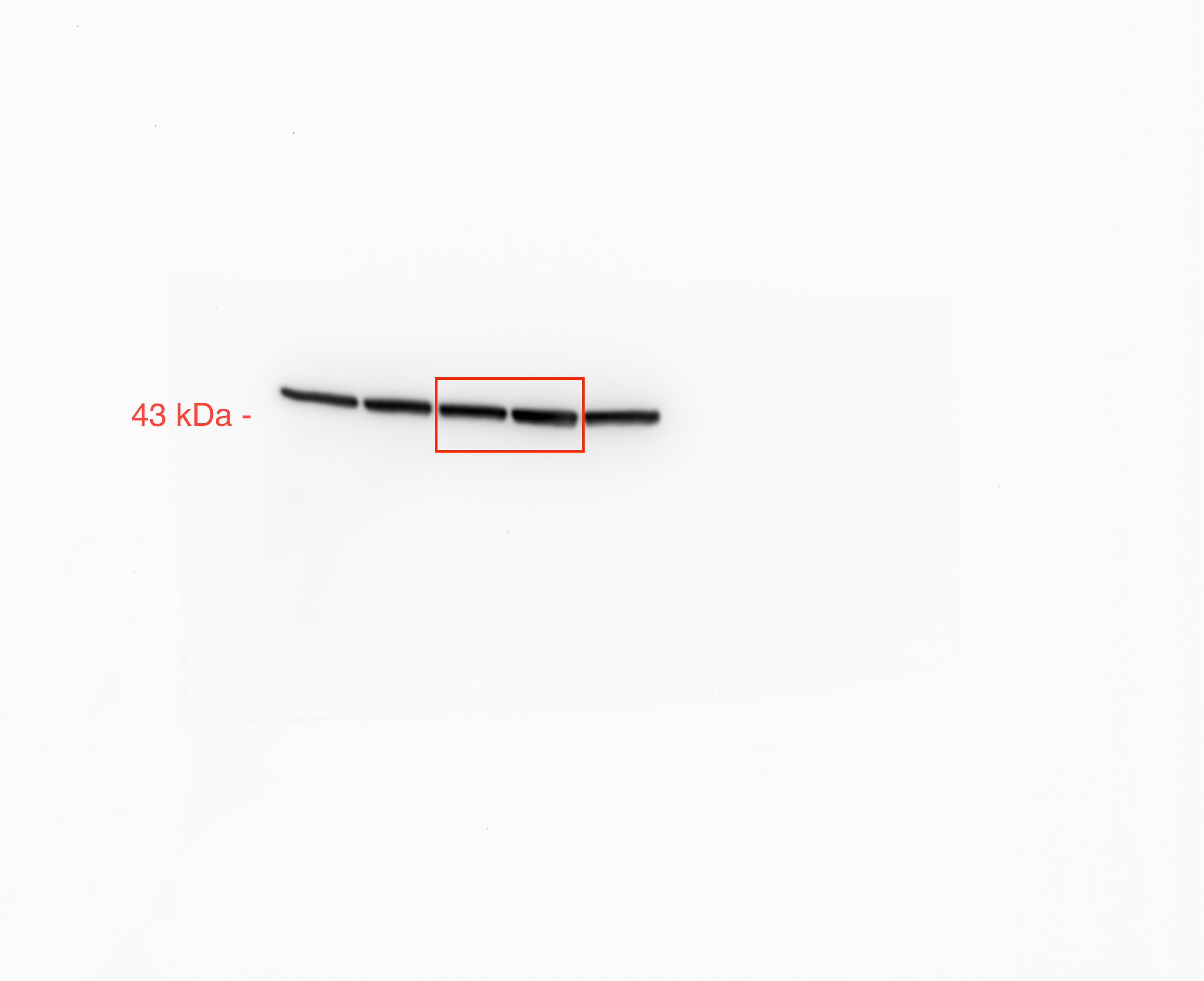

Supplement: Supplementary file 6 — Source data Fig. 2 [file 44318_2024_269_MOESM6_ESM.zip › Figure 2/2B/ACTIN.tif]

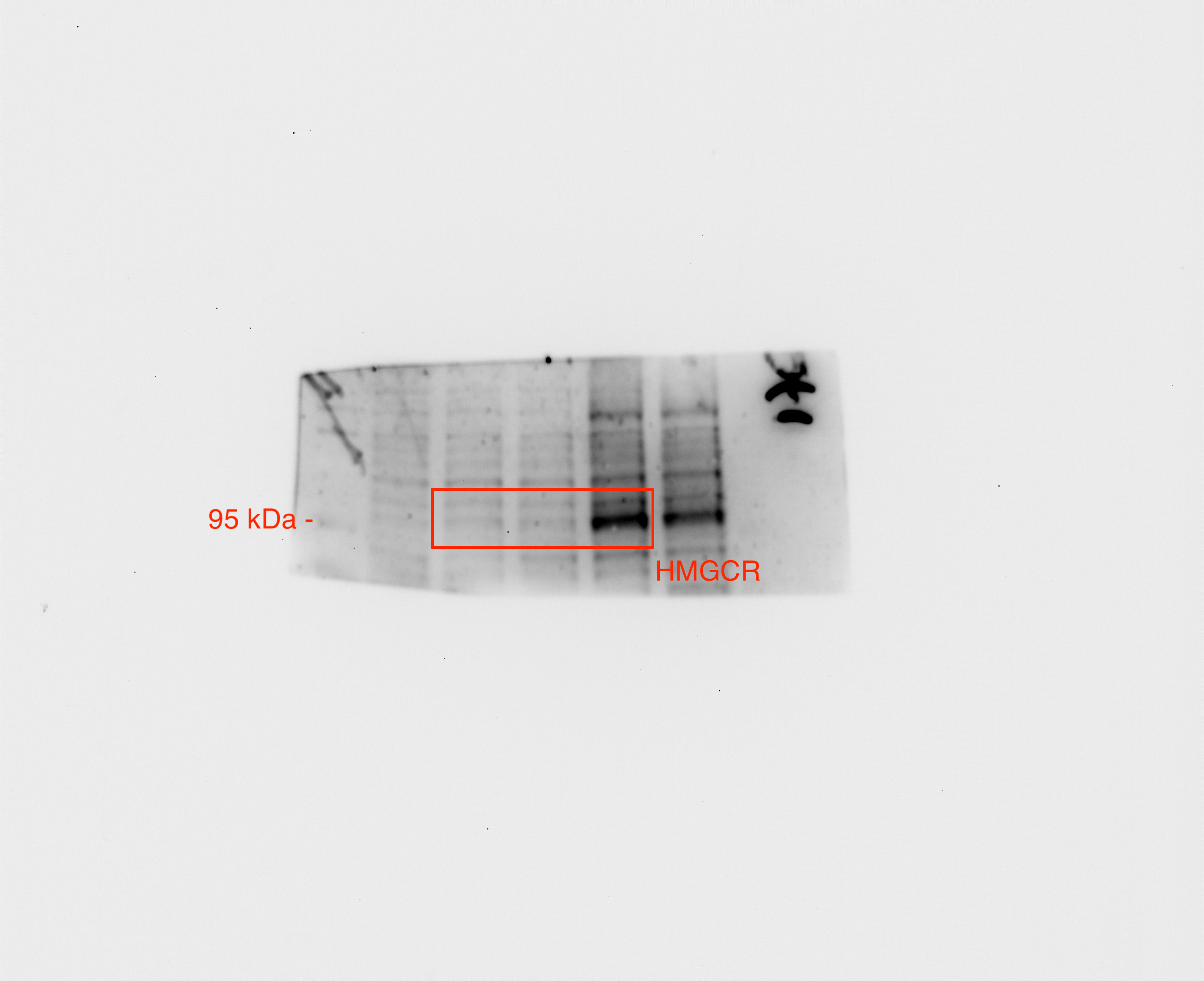

Supplement: Supplementary file 7 — Source data Fig. 3 [file 44318_2024_269_MOESM7_ESM.zip › Figure 3/3B/HMGCR.tiff]

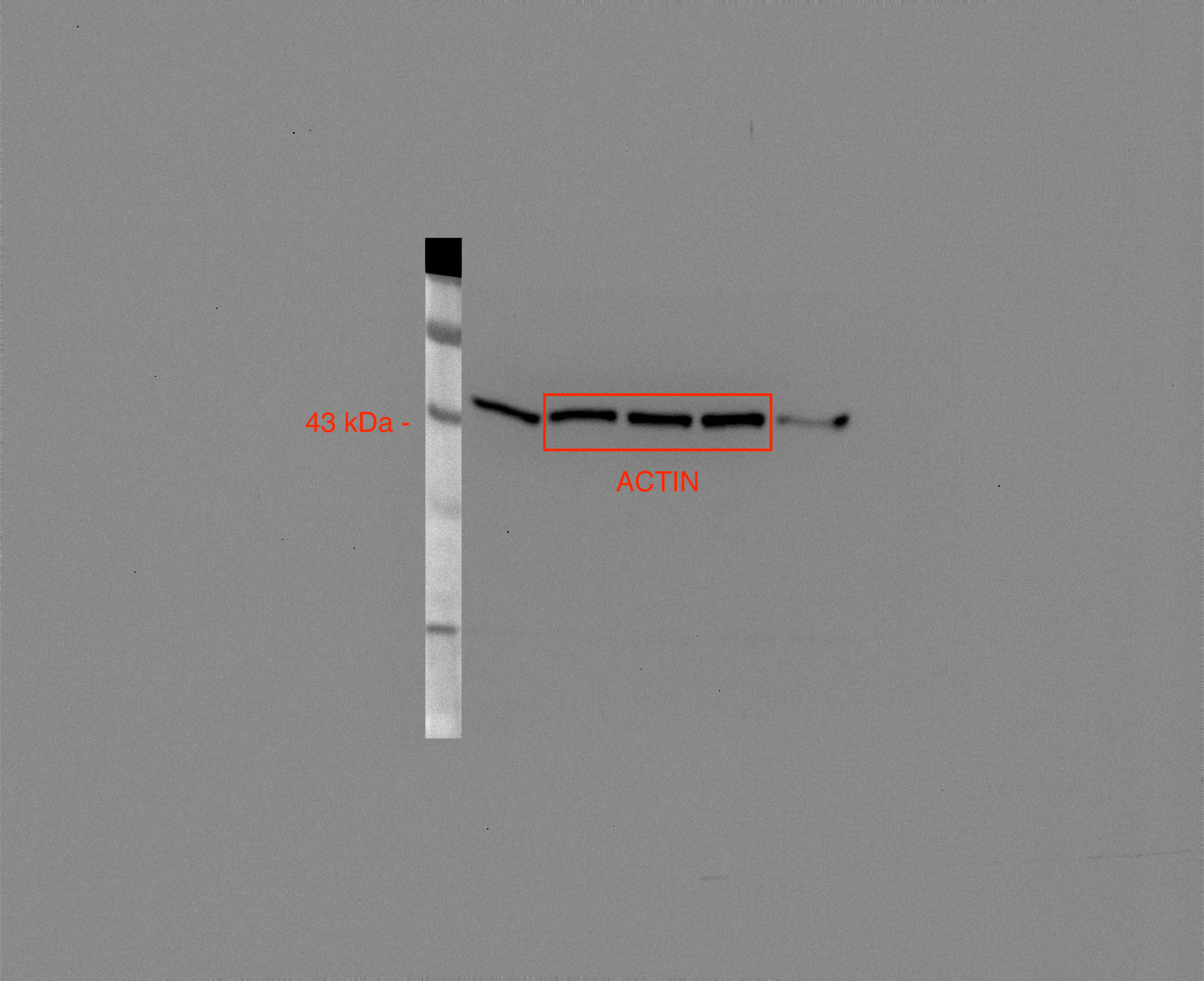

Supplement: Supplementary file 7 — Source data Fig. 3 [file 44318_2024_269_MOESM7_ESM.zip › Figure 3/3B/ACTIN.tiff]

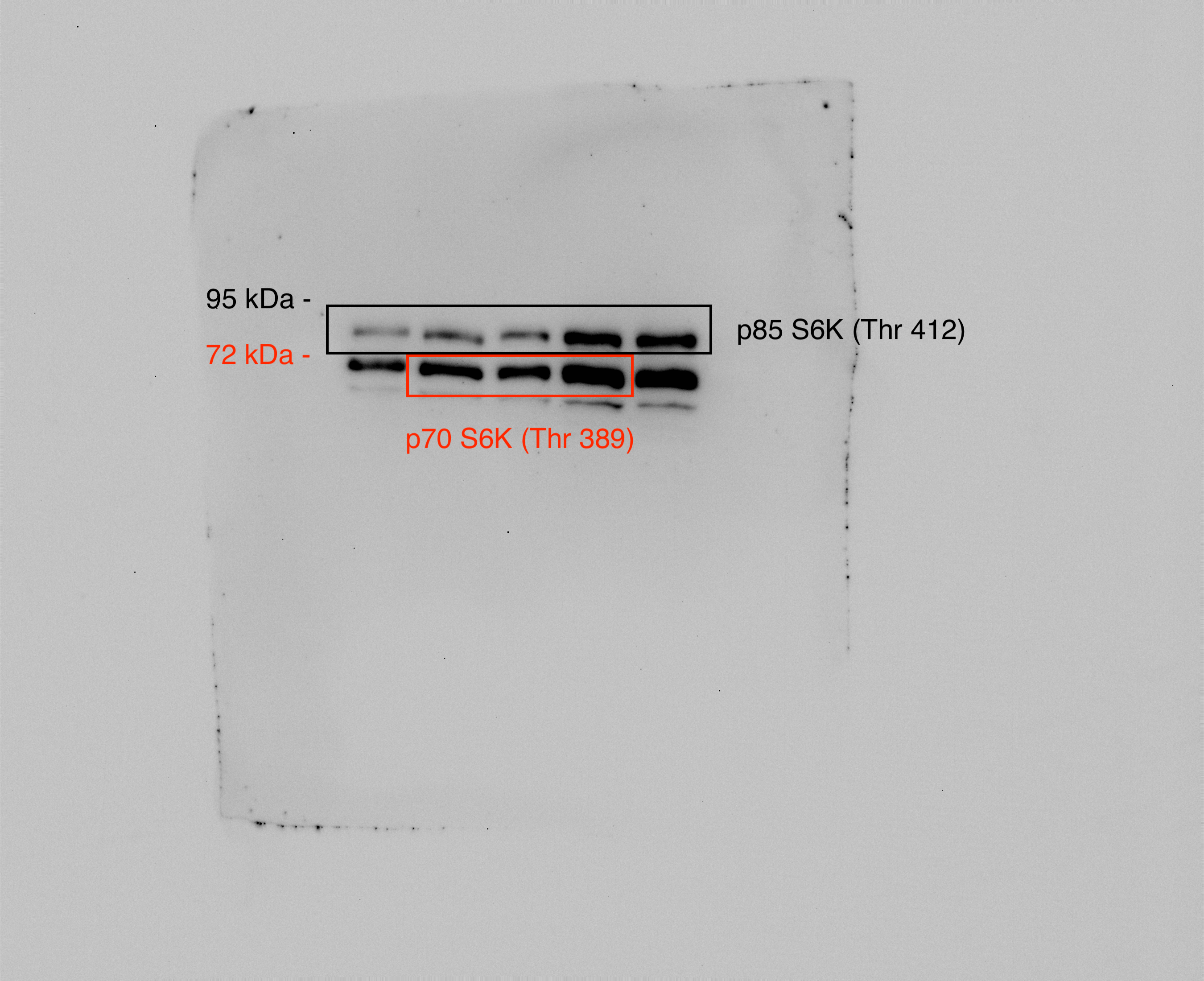

Supplement: Supplementary file 7 — Source data Fig. 3 [file 44318_2024_269_MOESM7_ESM.zip › Figure 3/3B/S6K.tiff]

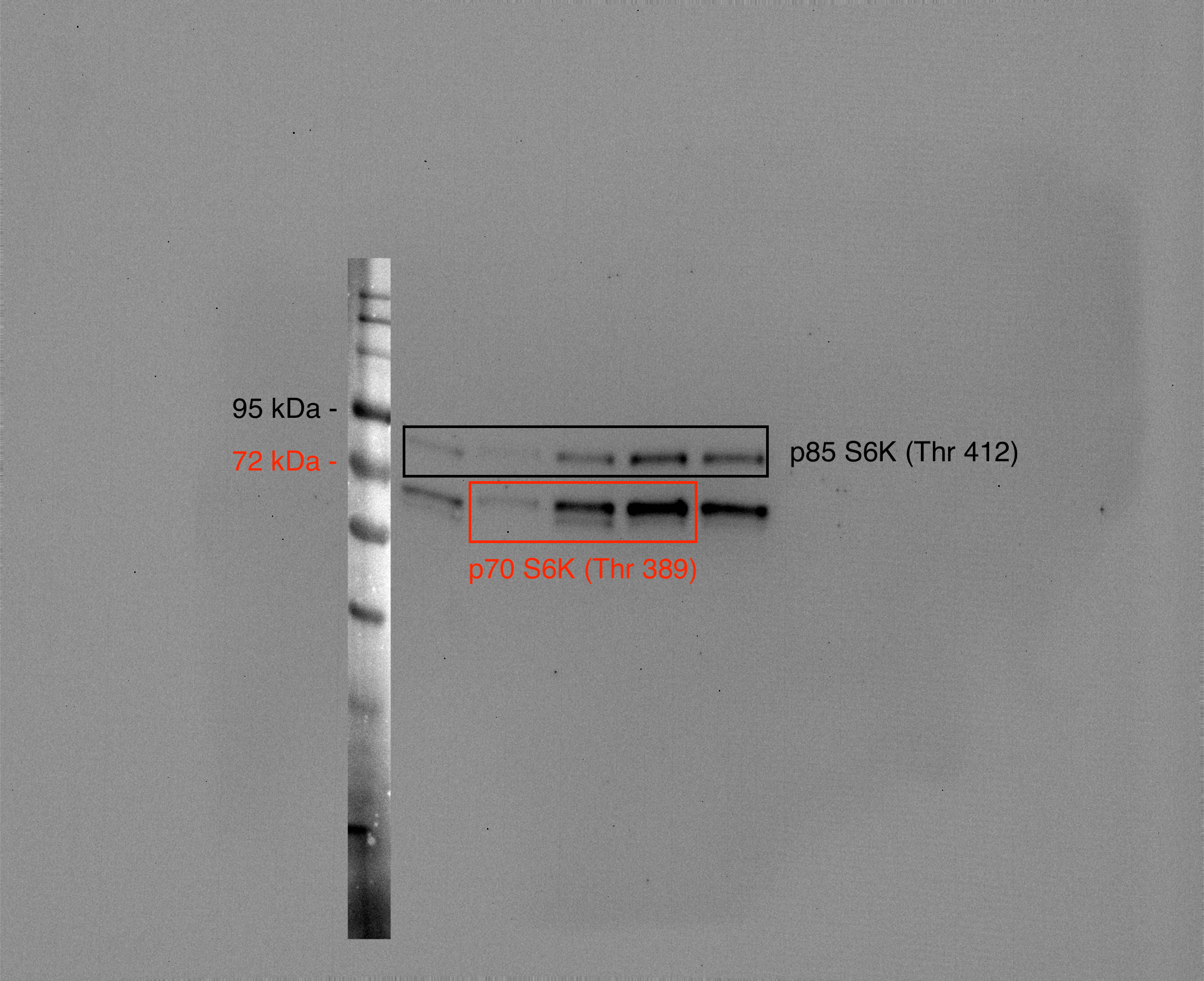

Supplement: Supplementary file 7 — Source data Fig. 3 [file 44318_2024_269_MOESM7_ESM.zip › Figure 3/3B/PS6K.tiff]

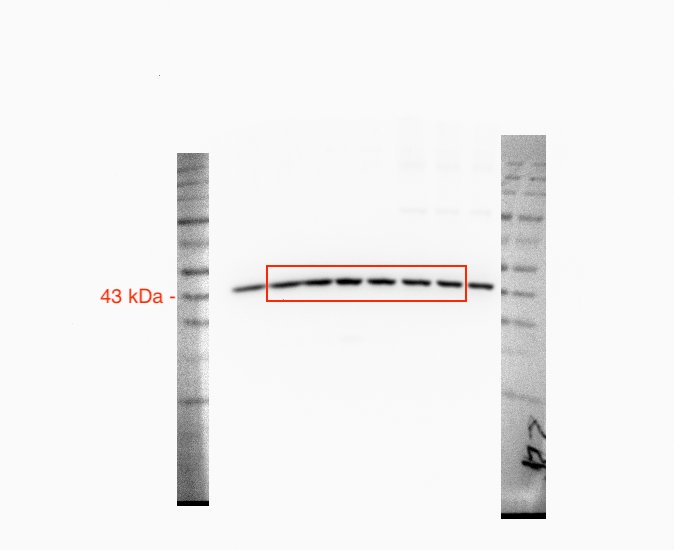

Supplement: Supplementary file 7 — Source data Fig. 3 [file 44318_2024_269_MOESM7_ESM.zip › Figure 3/3C/ACTIN.jpg]

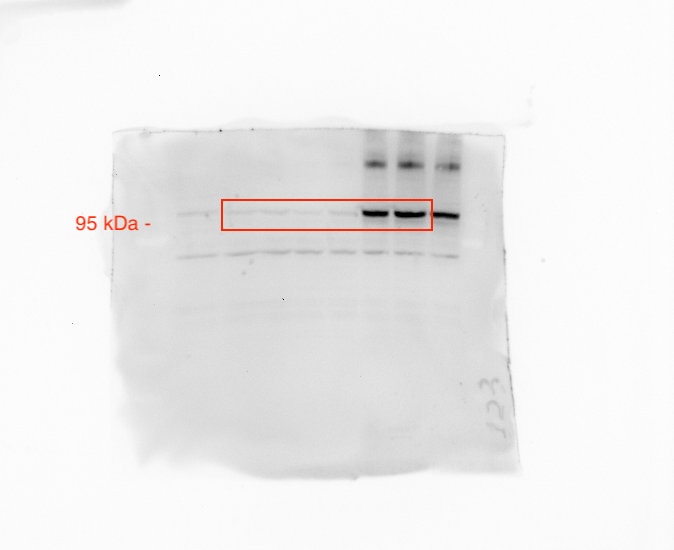

Supplement: Supplementary file 7 — Source data Fig. 3 [file 44318_2024_269_MOESM7_ESM.zip › Figure 3/3C/HMGCR.jpg]

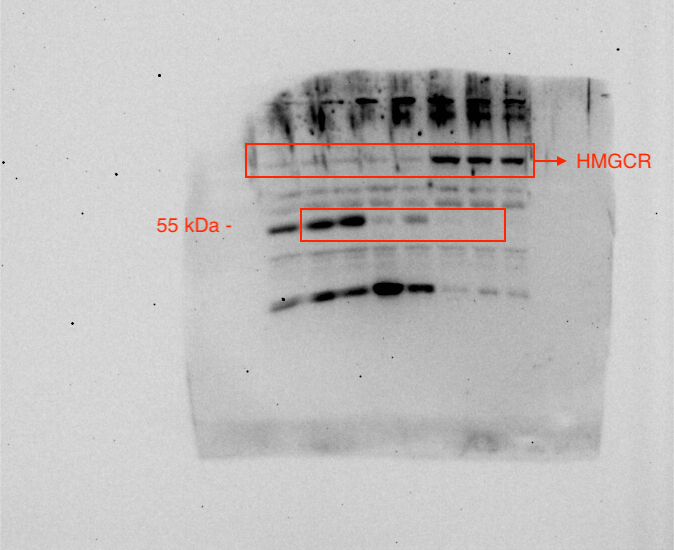

Supplement: Supplementary file 7 — Source data Fig. 3 [file 44318_2024_269_MOESM7_ESM.zip › Figure 3/3C/ATF4.tif]

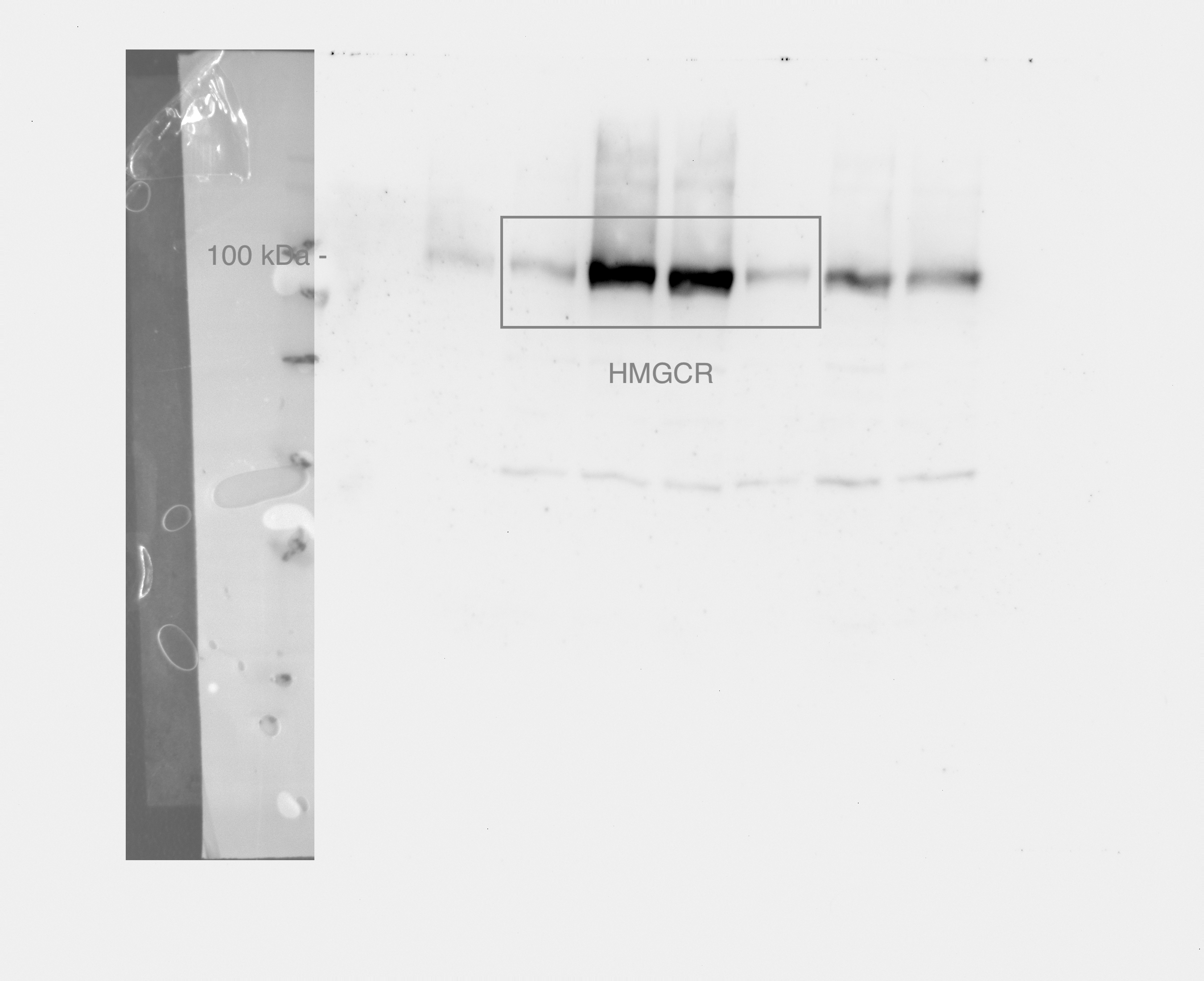

Supplement: Supplementary file 7 — Source data Fig. 3 [file 44318_2024_269_MOESM7_ESM.zip › Figure 3/3D/HMGCR.tif]

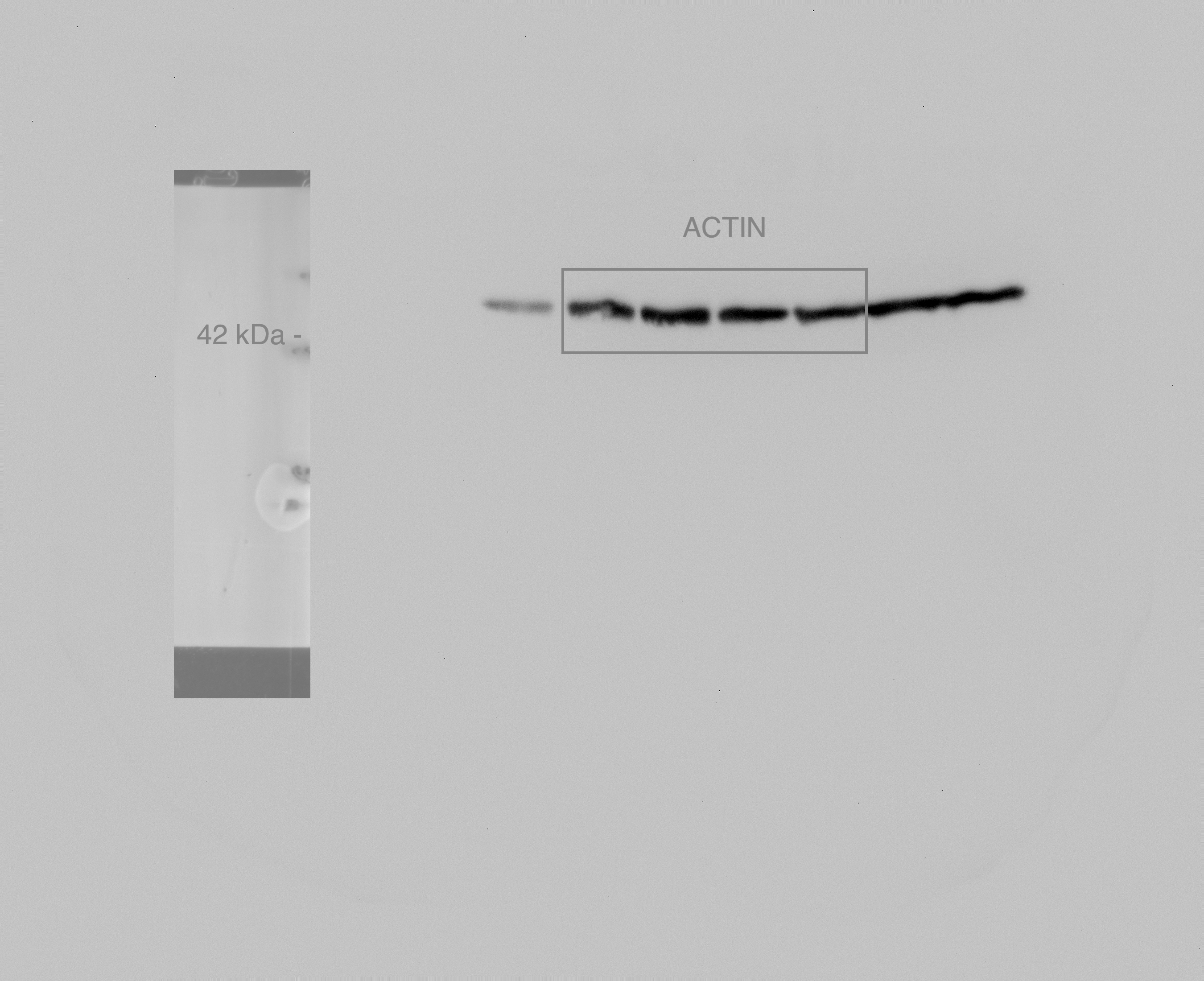

Supplement: Supplementary file 7 — Source data Fig. 3 [file 44318_2024_269_MOESM7_ESM.zip › Figure 3/3D/ACTIN.tif]

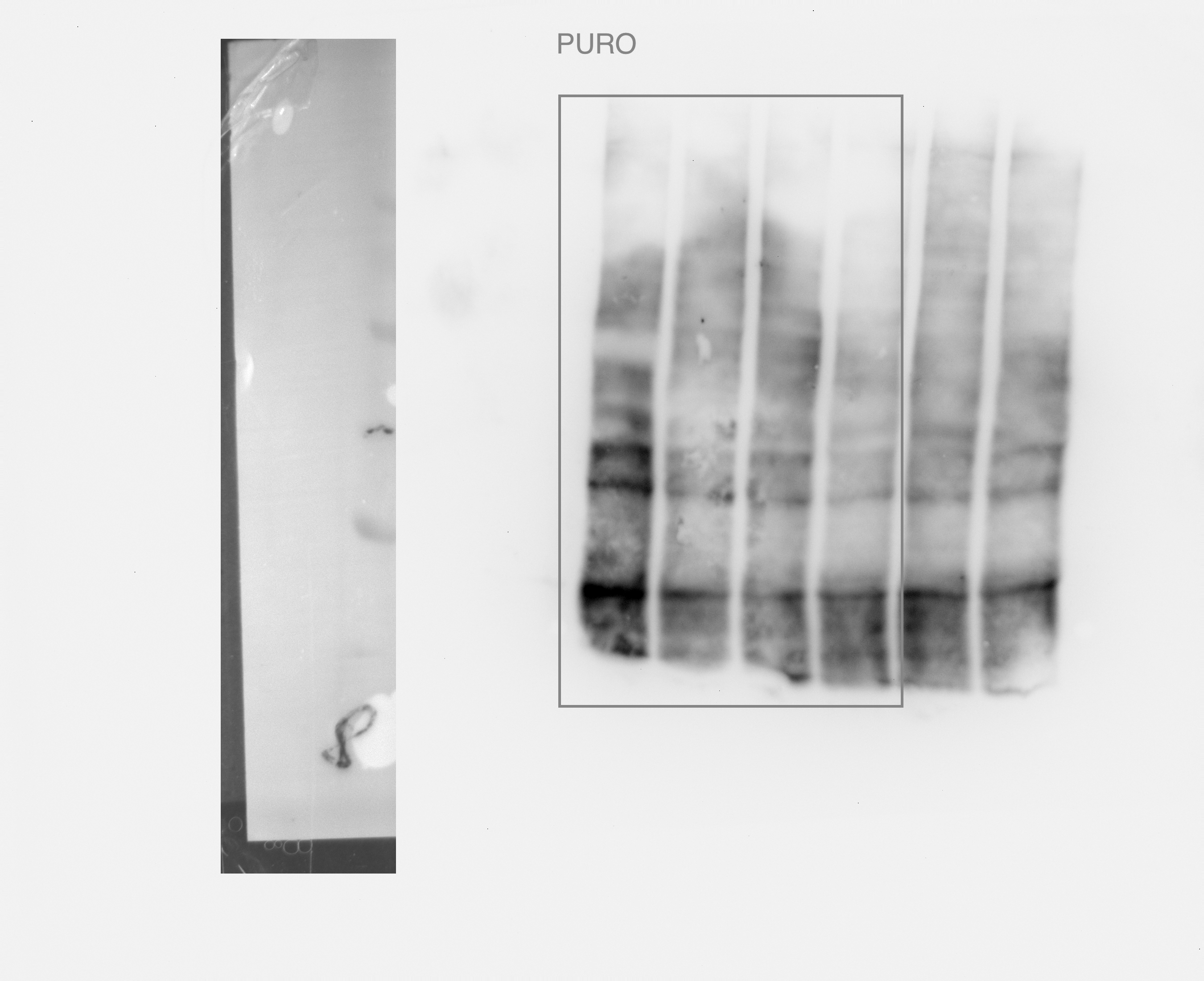

Supplement: Supplementary file 7 — Source data Fig. 3 [file 44318_2024_269_MOESM7_ESM.zip › Figure 3/3D/PURO.jpg]

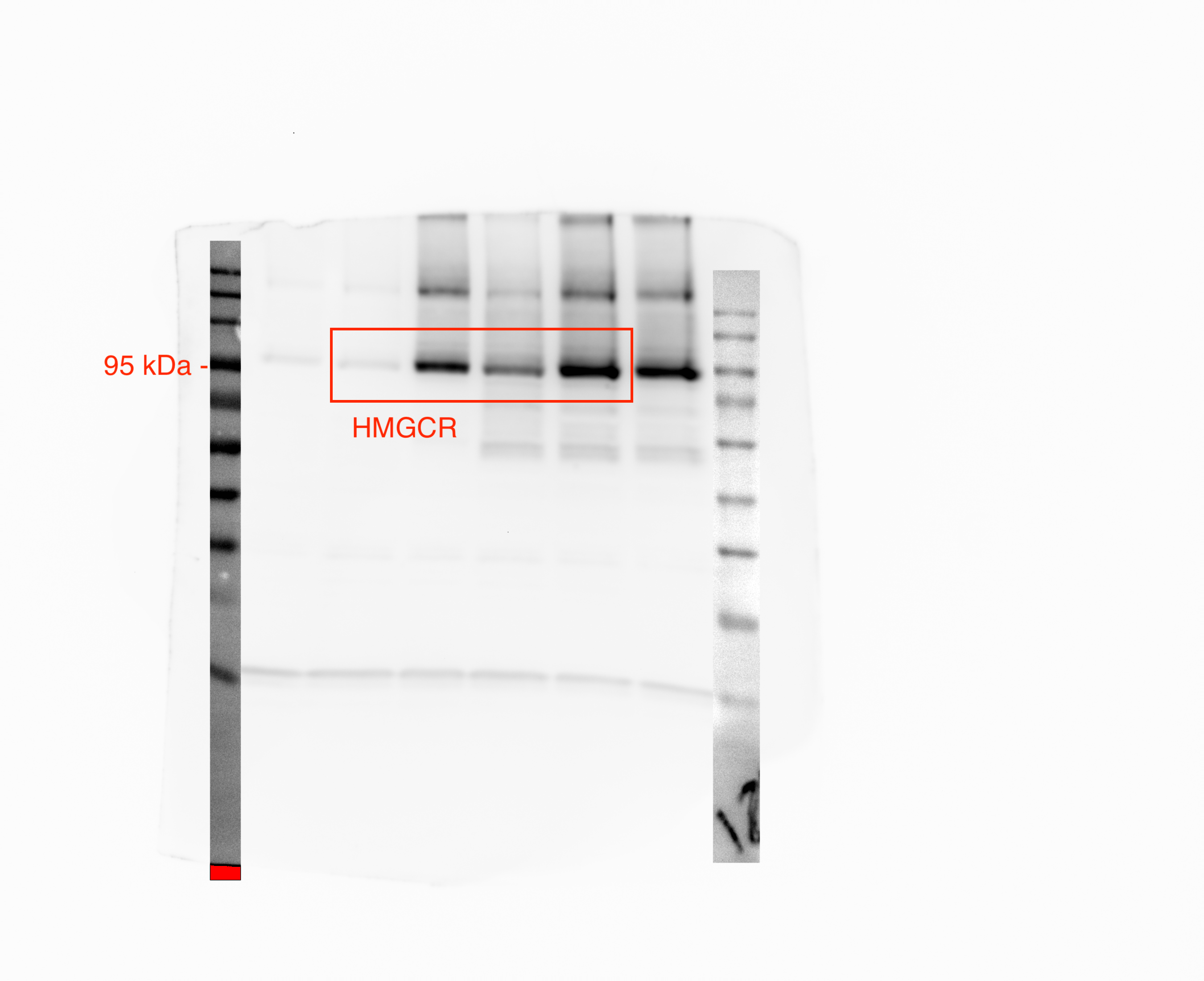

Supplement: Supplementary file 7 — Source data Fig. 3 [file 44318_2024_269_MOESM7_ESM.zip › Figure 3/3A/HMGCR.tiff]

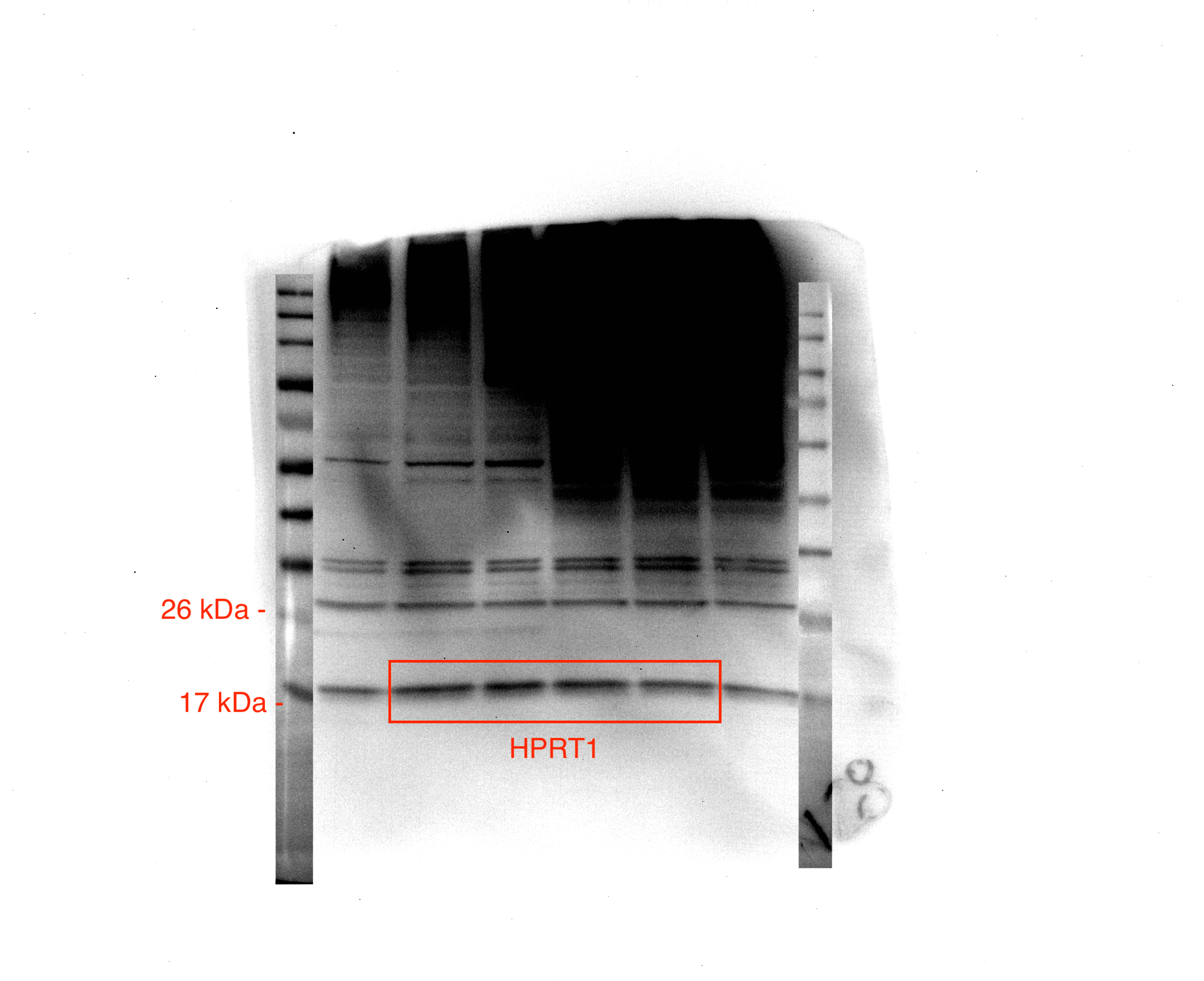

Supplement: Supplementary file 7 — Source data Fig. 3 [file 44318_2024_269_MOESM7_ESM.zip › Figure 3/3A/HPRT1.tiff]

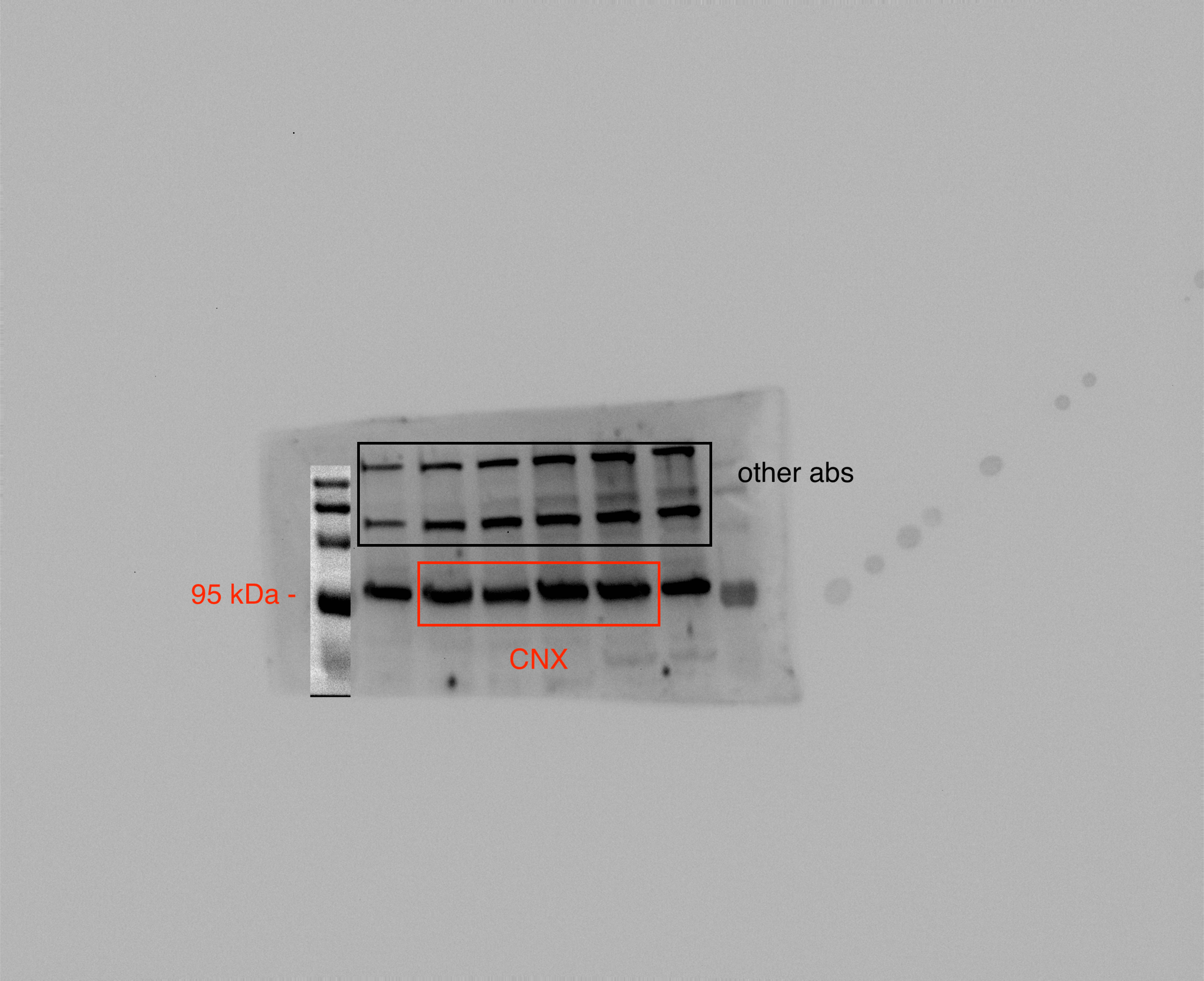

Supplement: Supplementary file 7 — Source data Fig. 3 [file 44318_2024_269_MOESM7_ESM.zip › Figure 3/3A/CNX.tiff]

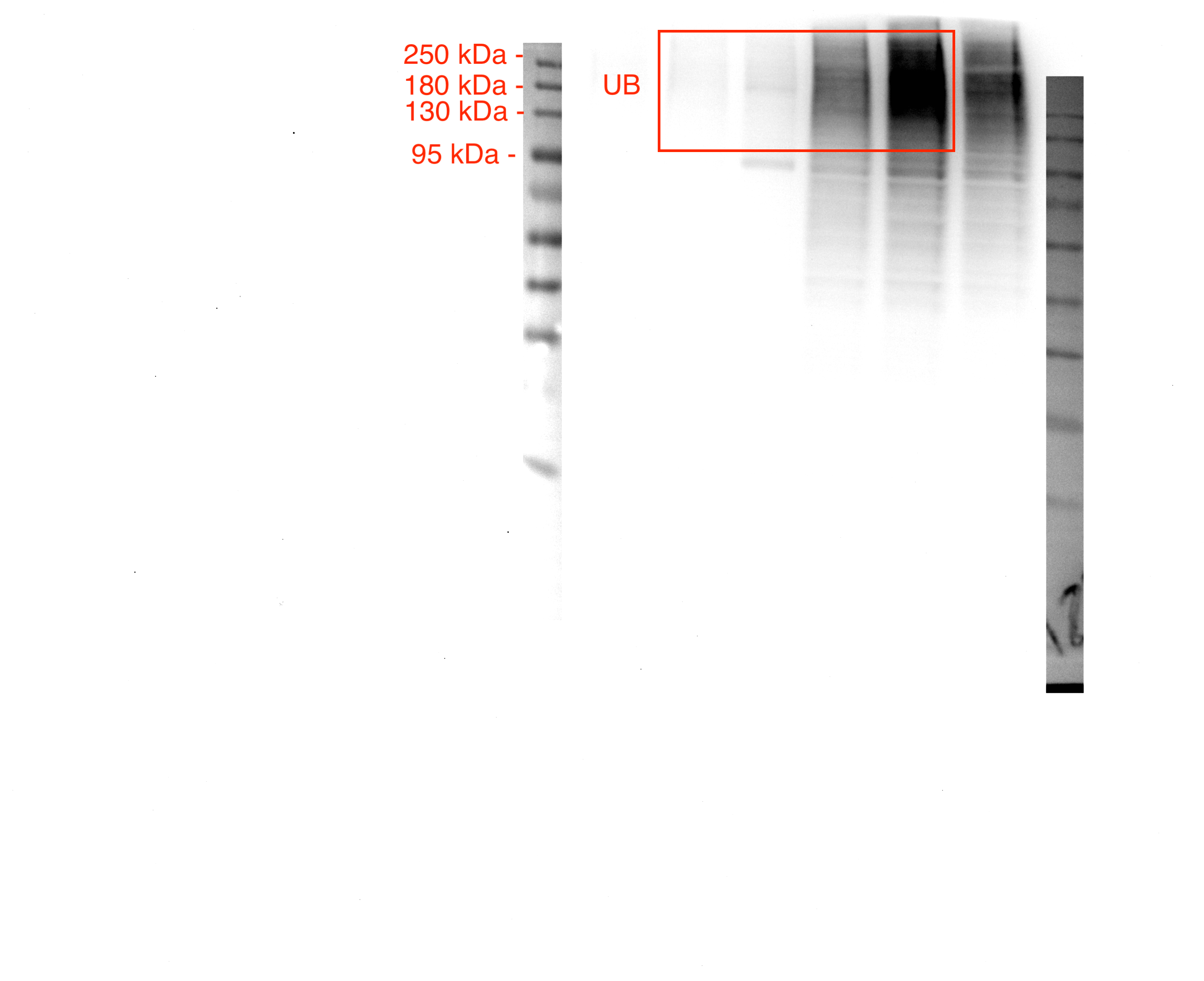

Supplement: Supplementary file 7 — Source data Fig. 3 [file 44318_2024_269_MOESM7_ESM.zip › Figure 3/3A/UB.tiff]

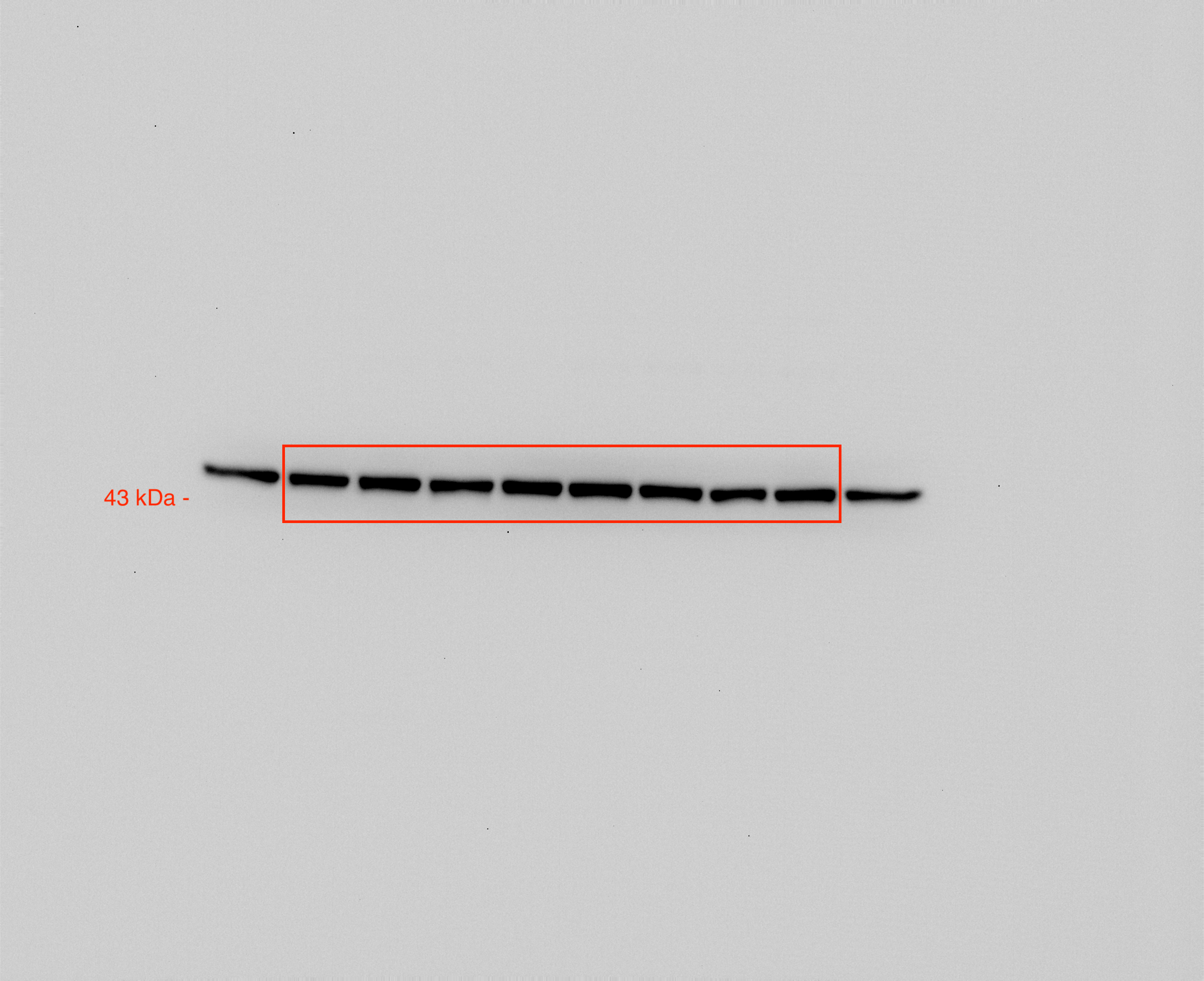

Supplement: Supplementary file 7 — Source data Fig. 3 [file 44318_2024_269_MOESM7_ESM.zip › Figure 3/3I/ACTIN.tif]

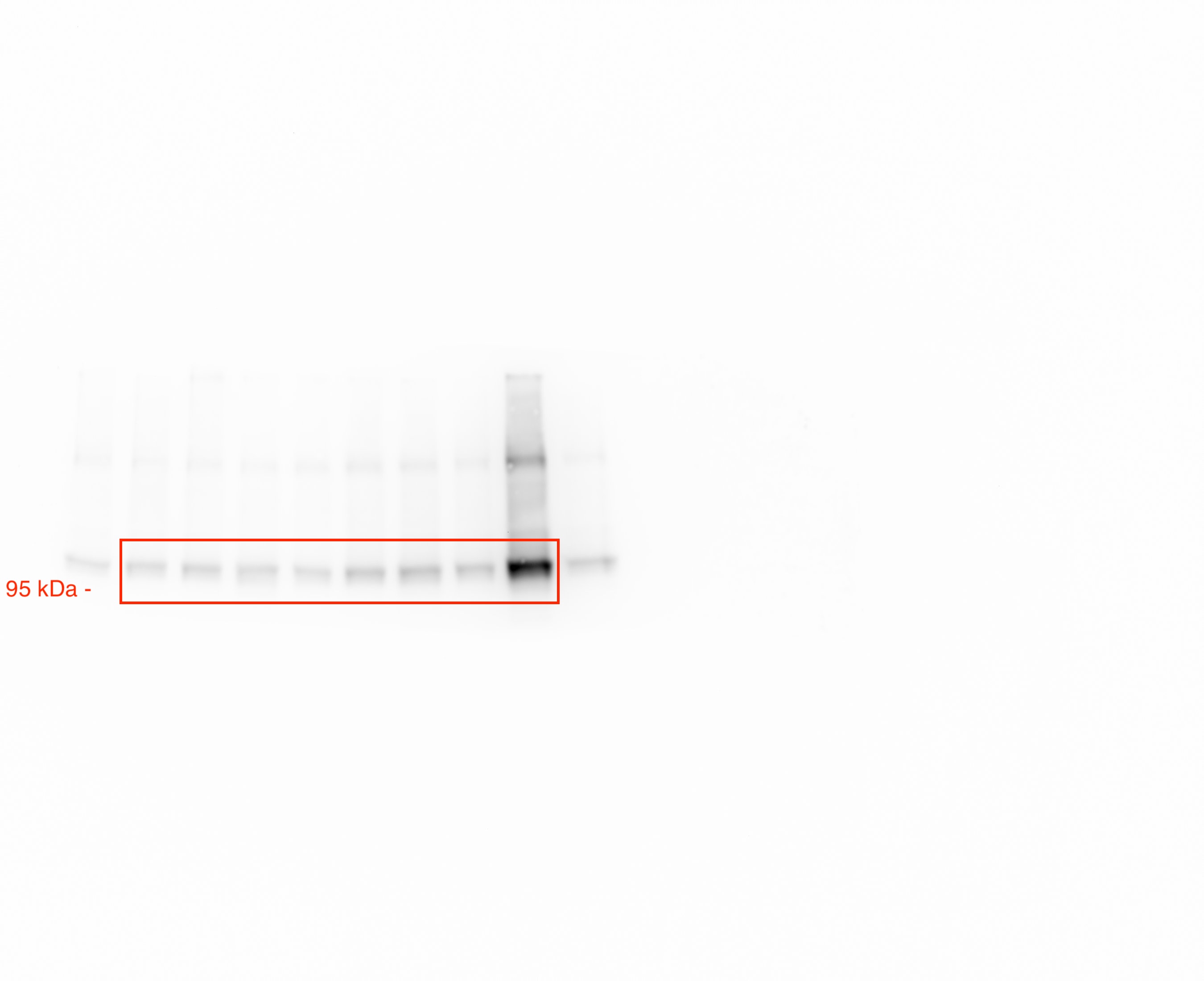

Supplement: Supplementary file 7 — Source data Fig. 3 [file 44318_2024_269_MOESM7_ESM.zip › Figure 3/3I/HMGCR.jpg]

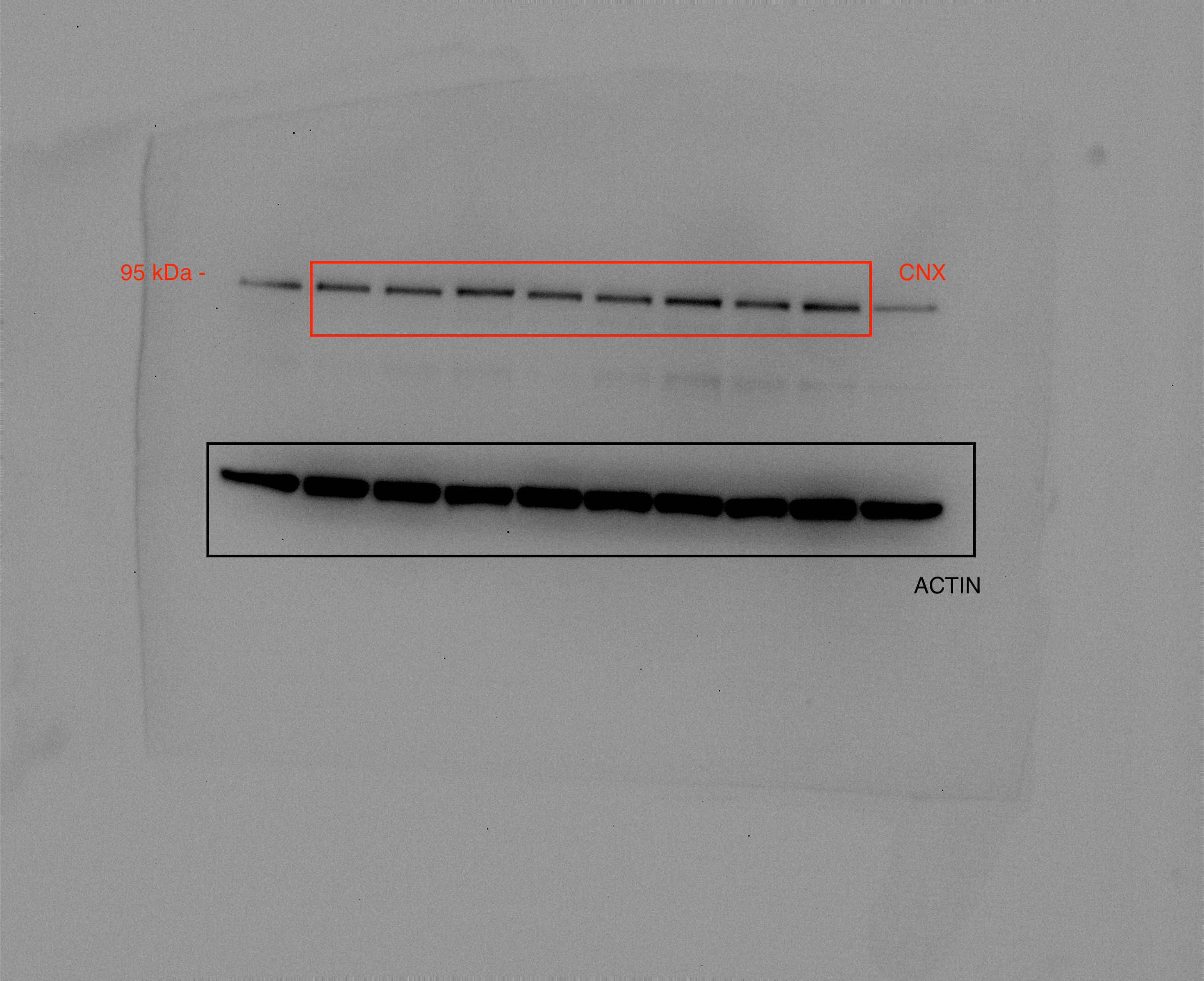

Supplement: Supplementary file 7 — Source data Fig. 3 [file 44318_2024_269_MOESM7_ESM.zip › Figure 3/3I/cnx.tiff]

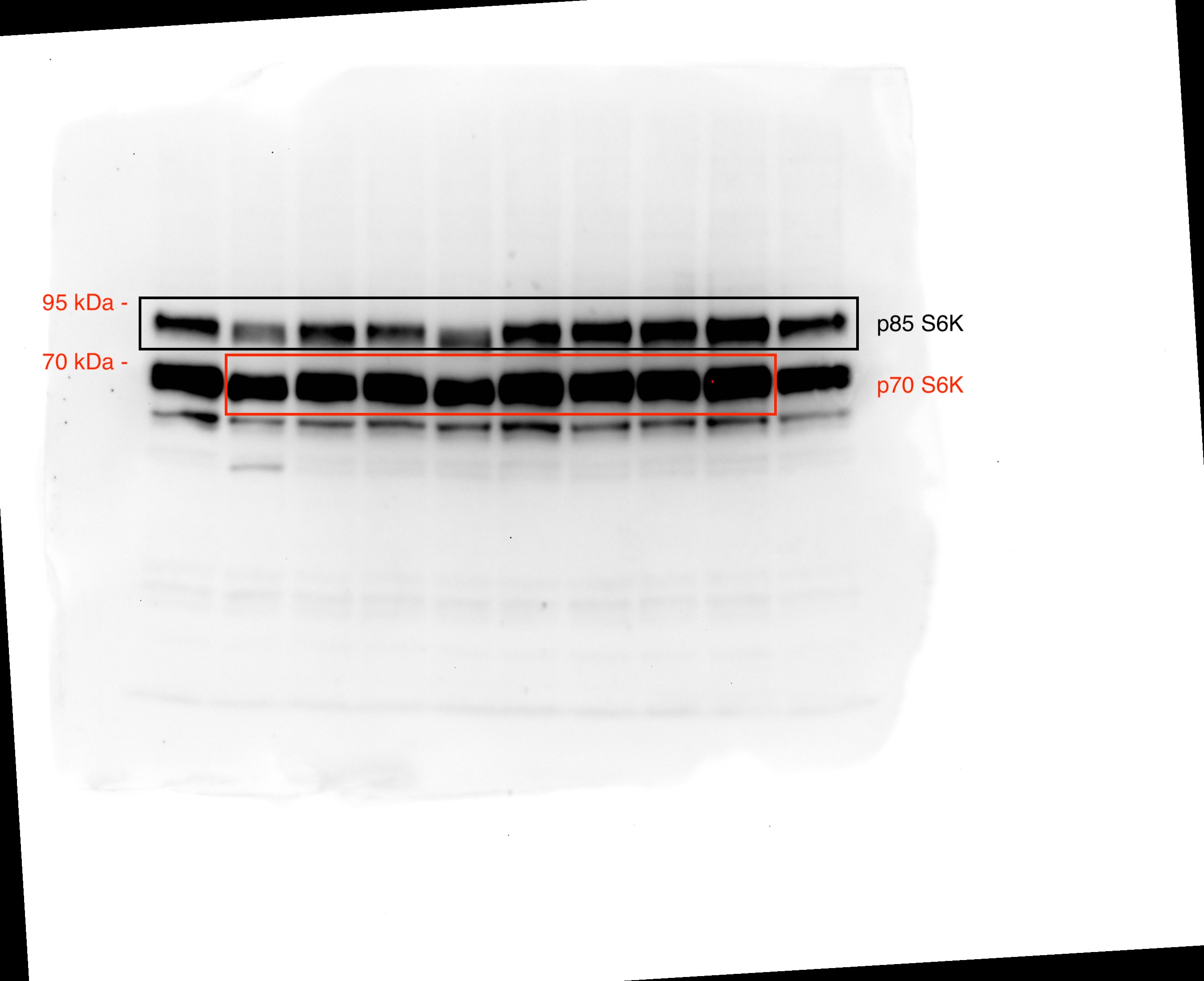

Supplement: Supplementary file 7 — Source data Fig. 3 [file 44318_2024_269_MOESM7_ESM.zip › Figure 3/3I/tS6K.jpg]

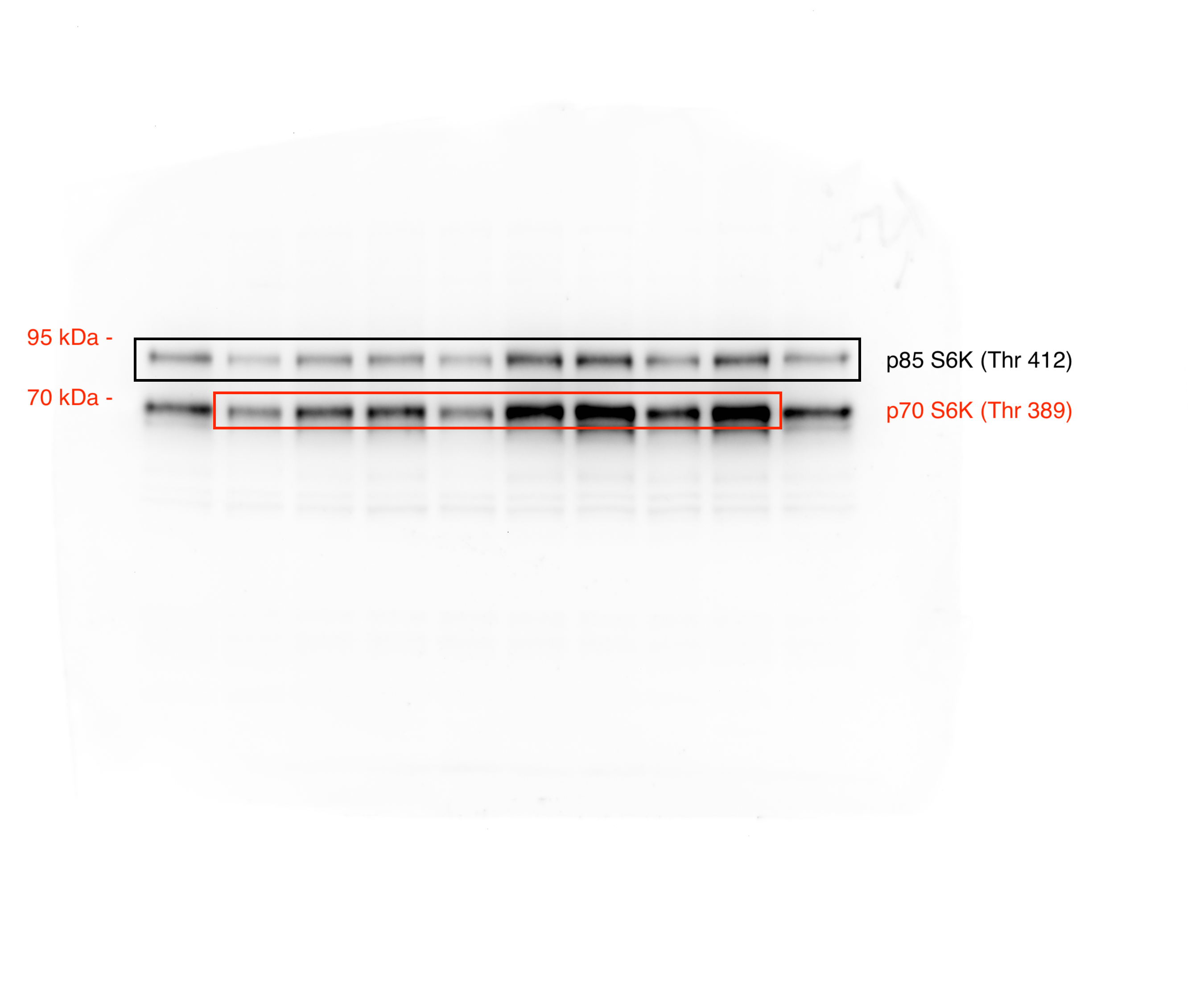

Supplement: Supplementary file 7 — Source data Fig. 3 [file 44318_2024_269_MOESM7_ESM.zip › Figure 3/3I/pS6K.tif]

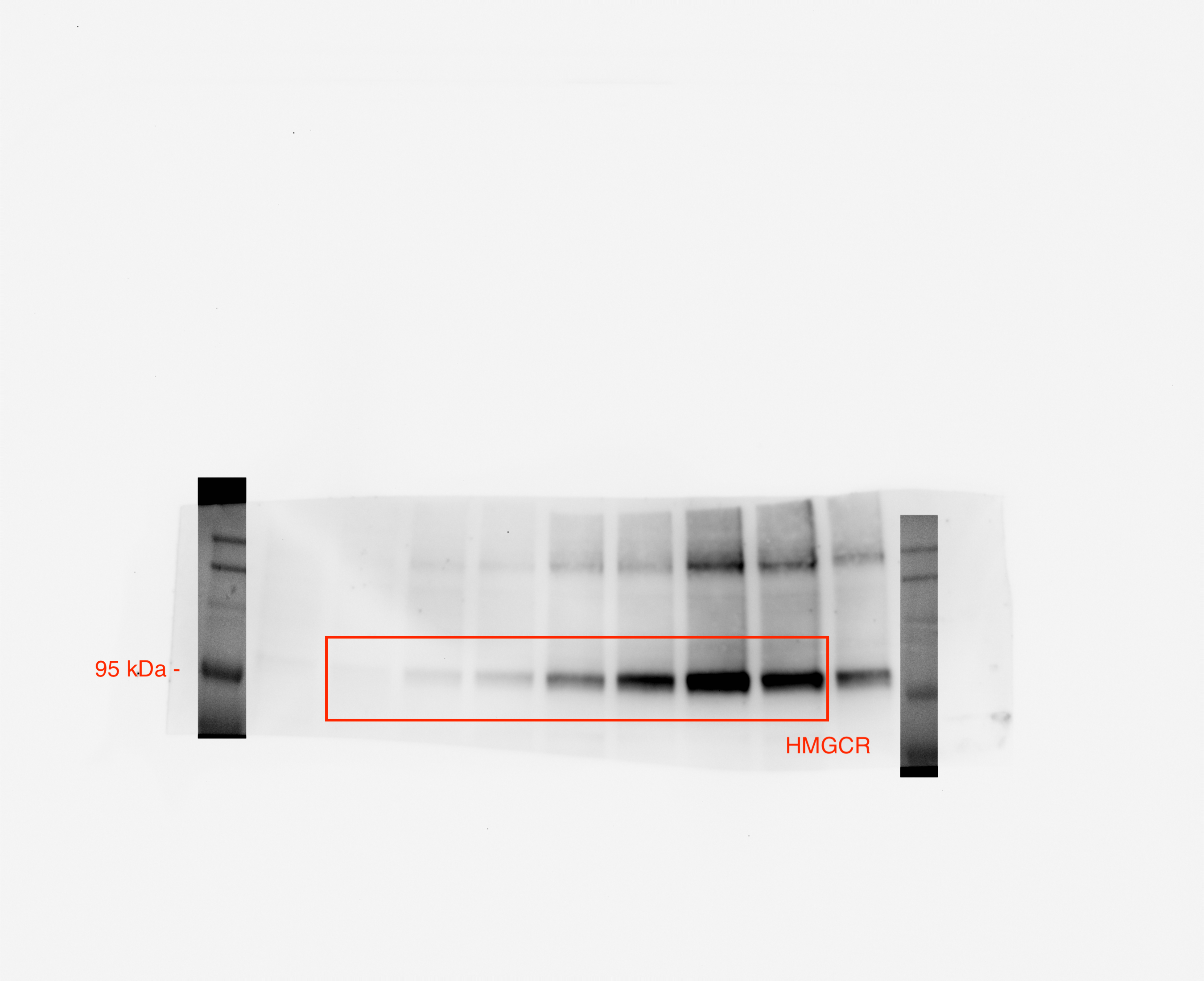

Supplement: Supplementary file 8 — Source data Fig. 4 [file 44318_2024_269_MOESM8_ESM.zip › Figure 4/4A/hmgcr.tiff]

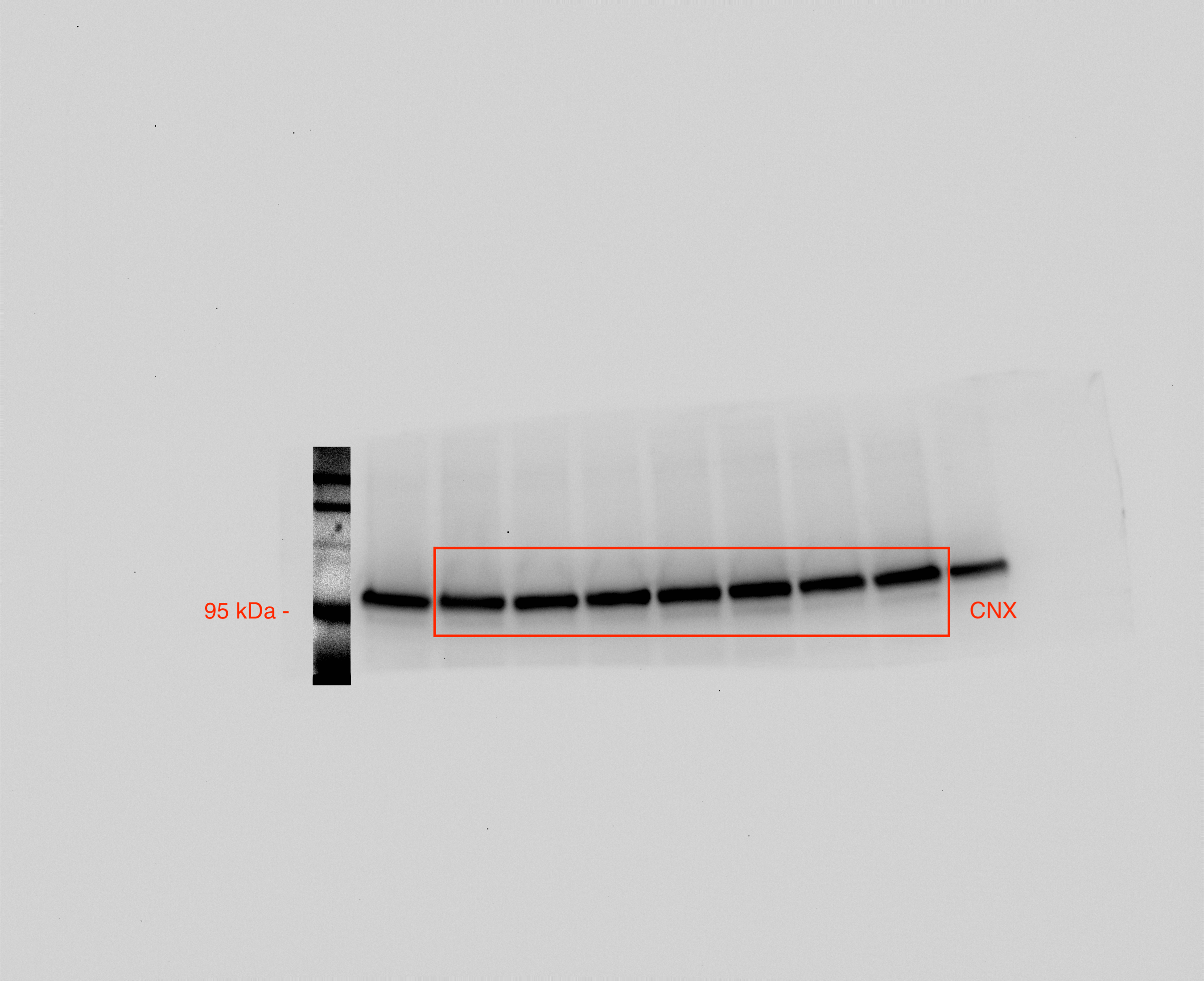

Supplement: Supplementary file 8 — Source data Fig. 4 [file 44318_2024_269_MOESM8_ESM.zip › Figure 4/4A/CNX.tiff]

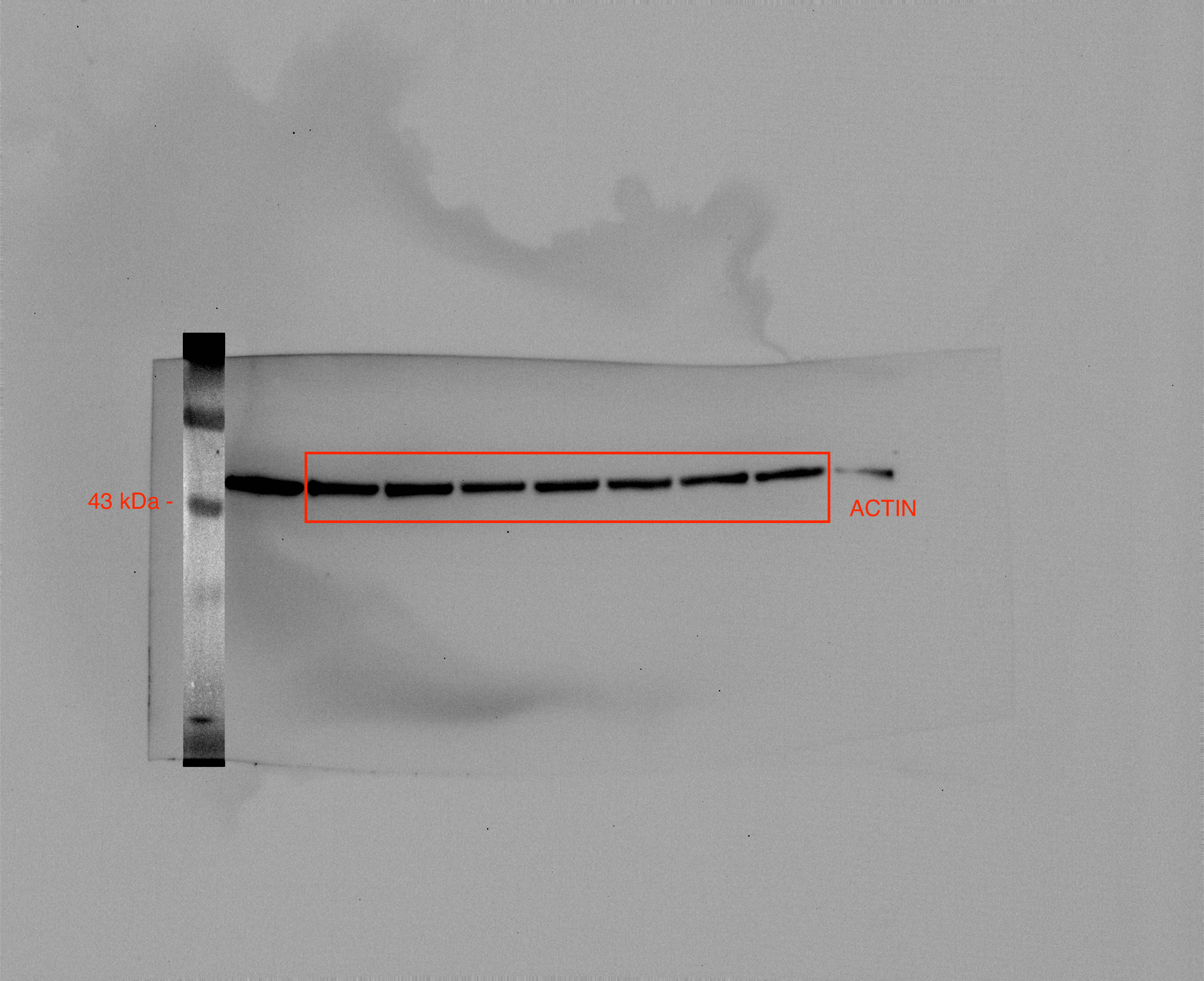

Supplement: Supplementary file 8 — Source data Fig. 4 [file 44318_2024_269_MOESM8_ESM.zip › Figure 4/4A/ACTIN.tiff]

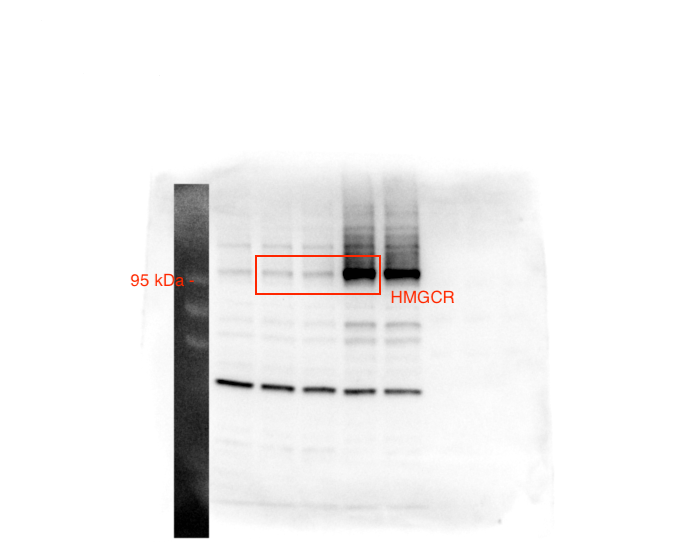

Supplement: Supplementary file 9 — Source data Fig. 5 [file 44318_2024_269_MOESM9_ESM.zip › Figure 5/5A/HMGCR.tiff]

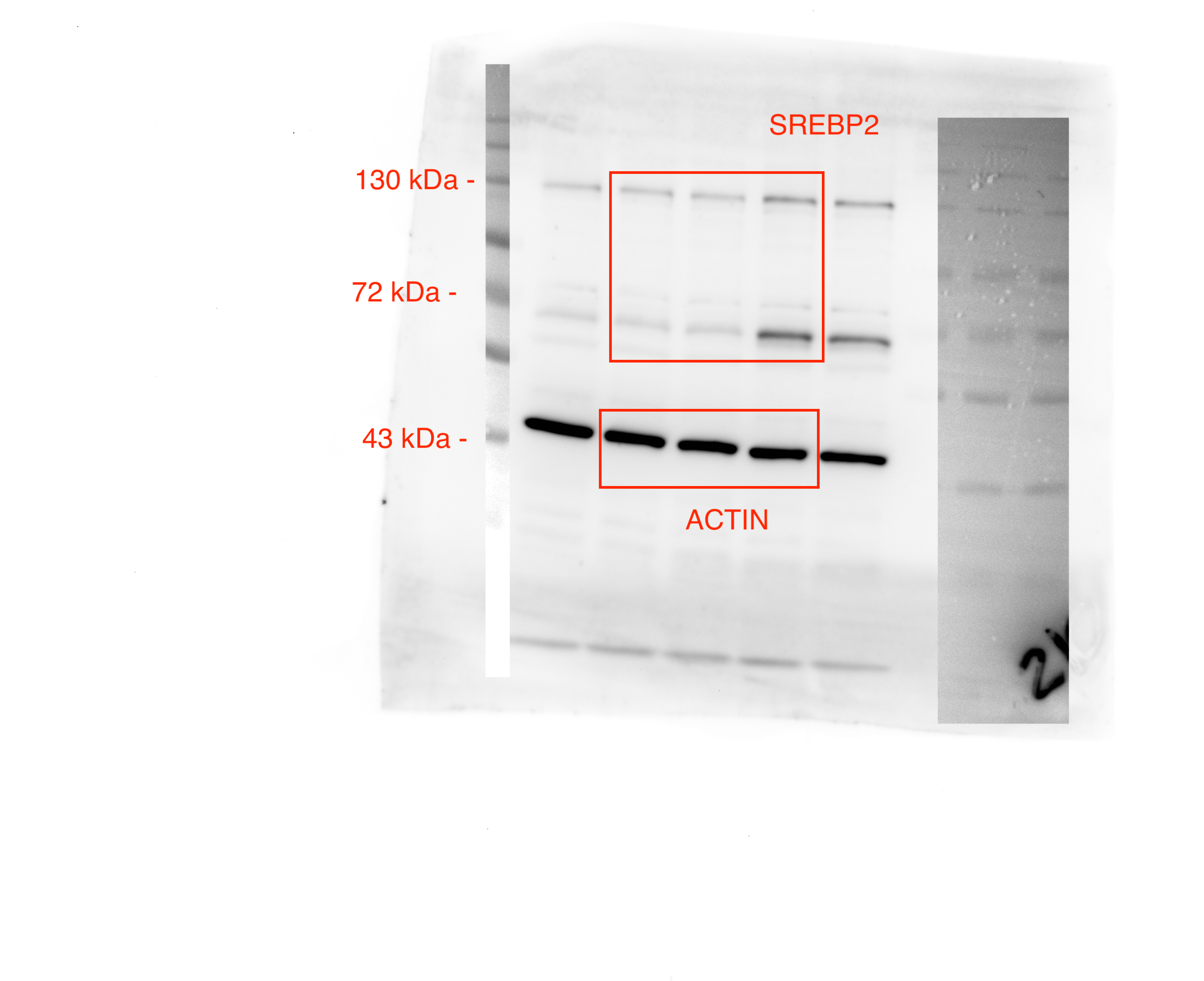

Supplement: Supplementary file 9 — Source data Fig. 5 [file 44318_2024_269_MOESM9_ESM.zip › Figure 5/5A/SREBP2 AND ACTIN.tiff]

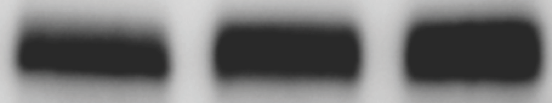

Supplement: Supplementary file 9 — Source data Fig. 5 [file 44318_2024_269_MOESM9_ESM.zip › Figure 5/5A/tS6K.png]

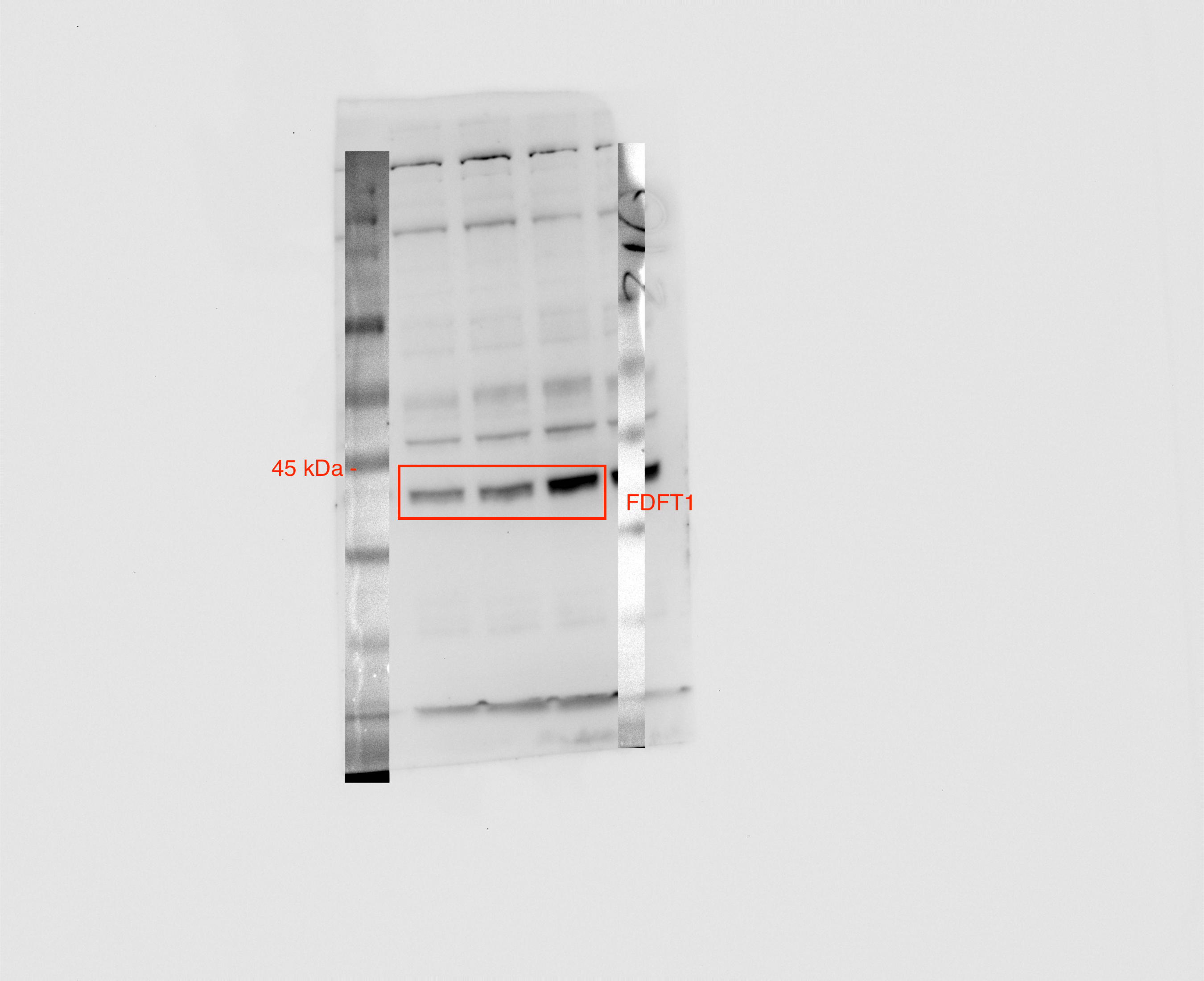

Supplement: Supplementary file 9 — Source data Fig. 5 [file 44318_2024_269_MOESM9_ESM.zip › Figure 5/5A/FDFT1.tiff]

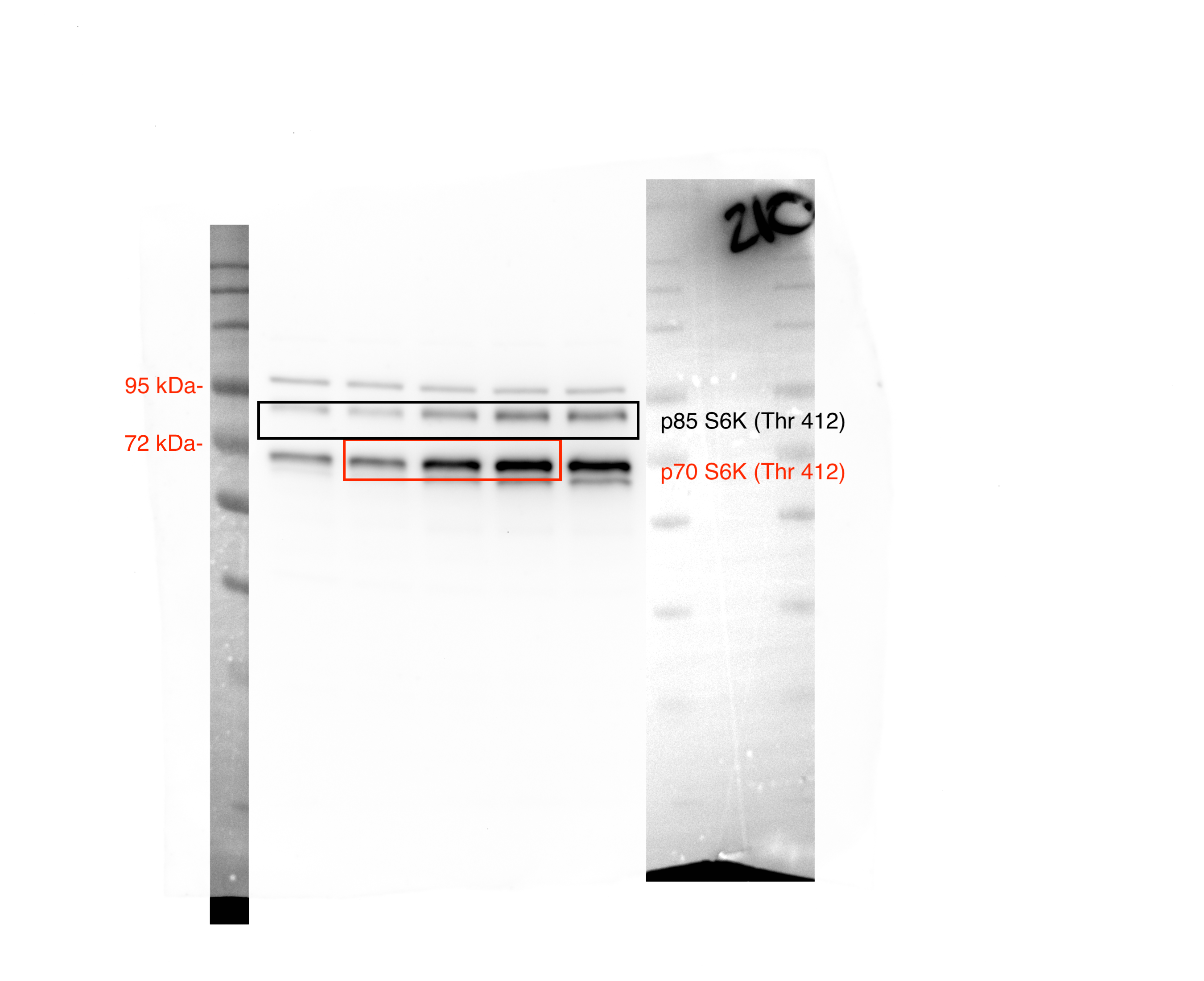

Supplement: Supplementary file 9 — Source data Fig. 5 [file 44318_2024_269_MOESM9_ESM.zip › Figure 5/5A/pS6K.tiff]

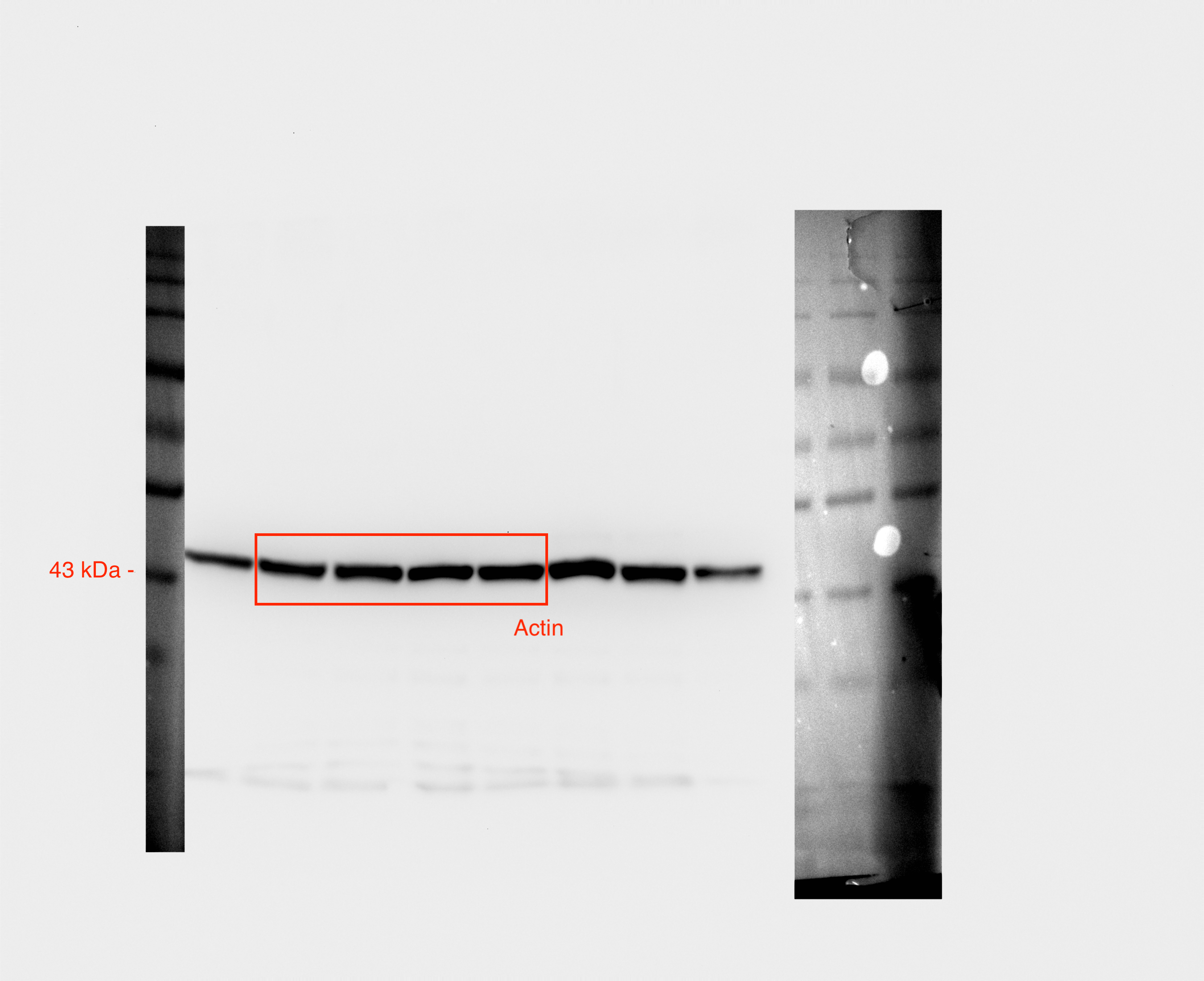

Supplement: Supplementary file 9 — Source data Fig. 5 [file 44318_2024_269_MOESM9_ESM.zip › Figure 5/5F/ACTIN.jpg]

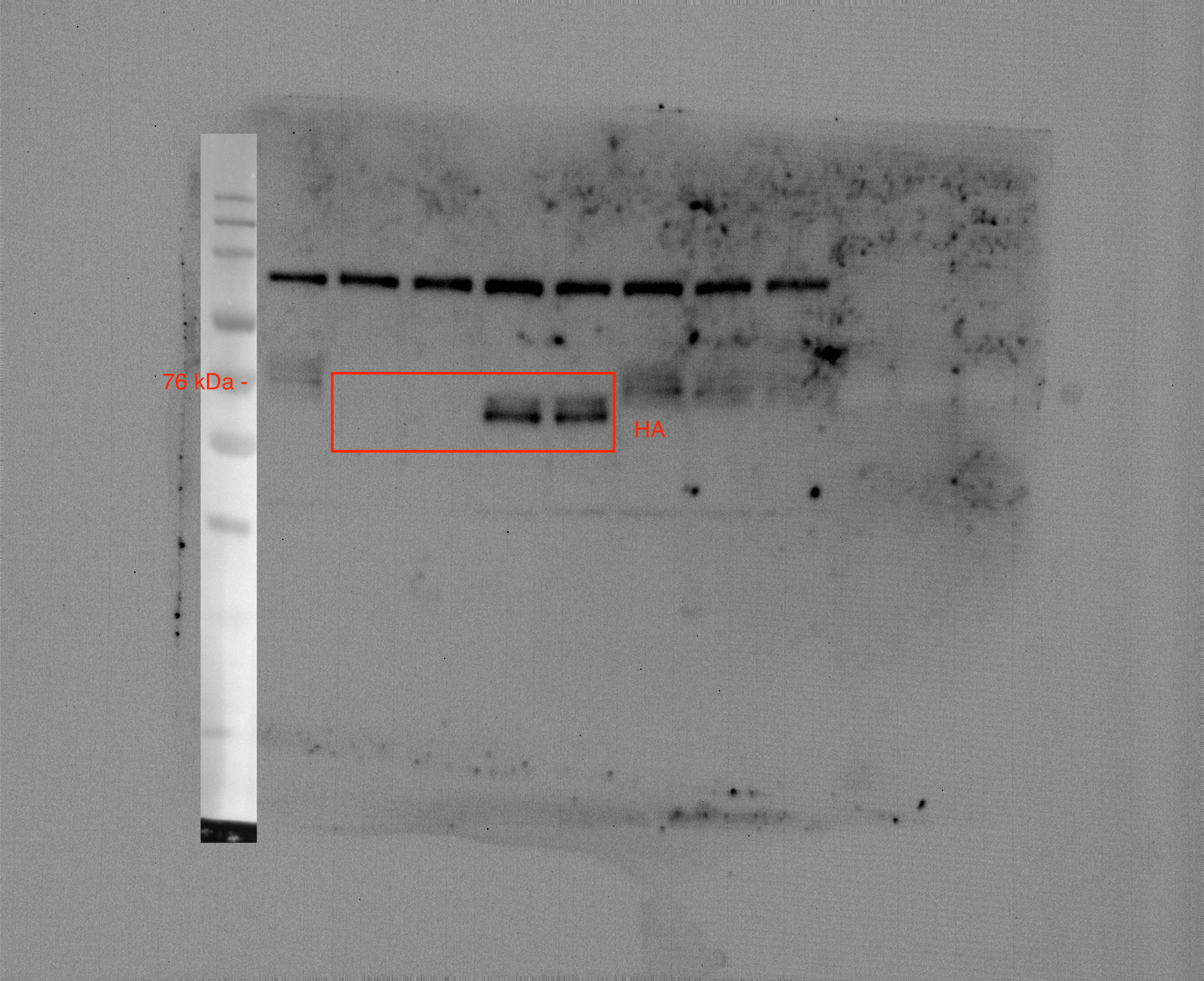

Supplement: Supplementary file 9 — Source data Fig. 5 [file 44318_2024_269_MOESM9_ESM.zip › Figure 5/5F/HA .jpg]

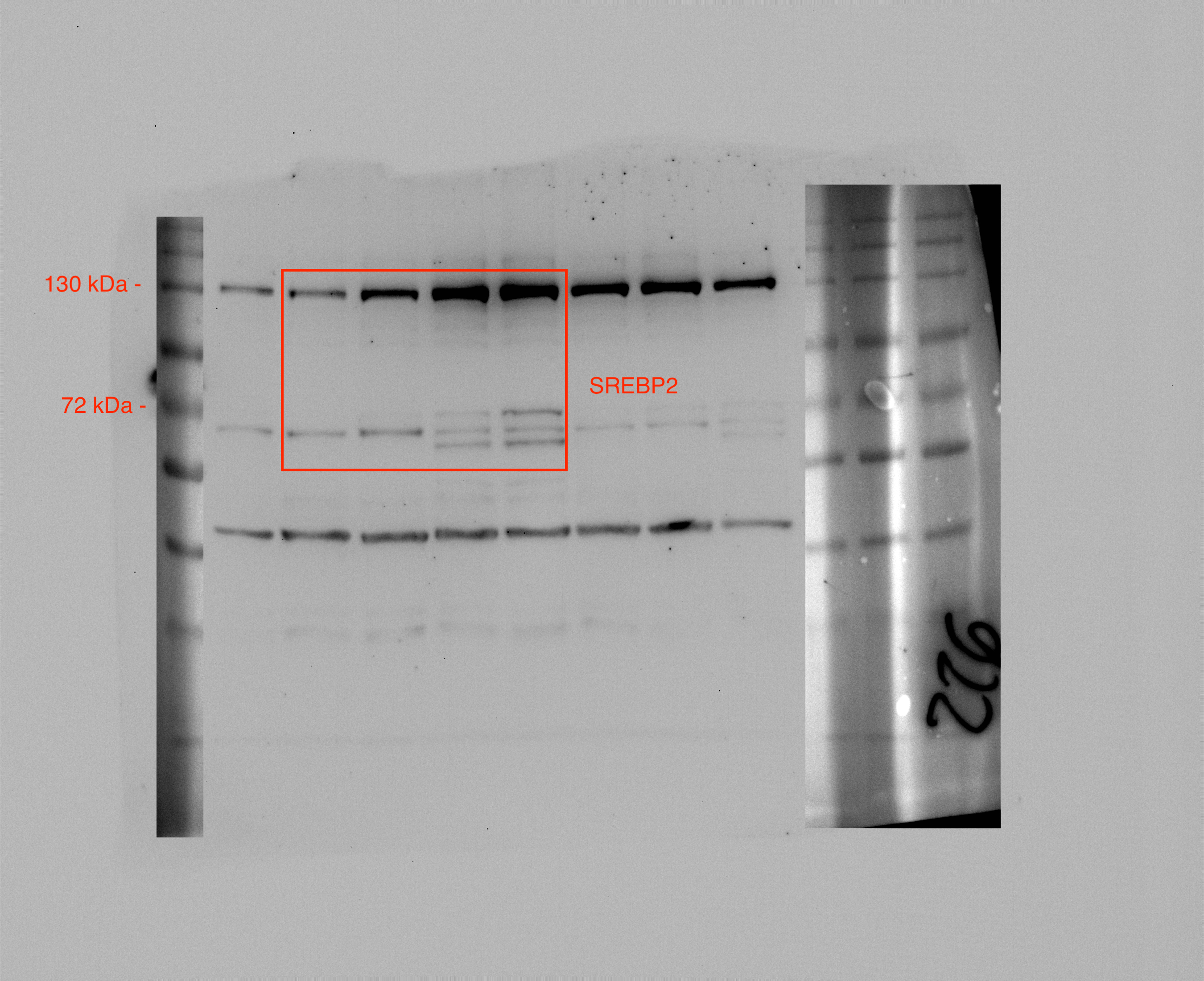

Supplement: Supplementary file 9 — Source data Fig. 5 [file 44318_2024_269_MOESM9_ESM.zip › Figure 5/5F/SREBP2.jpg]

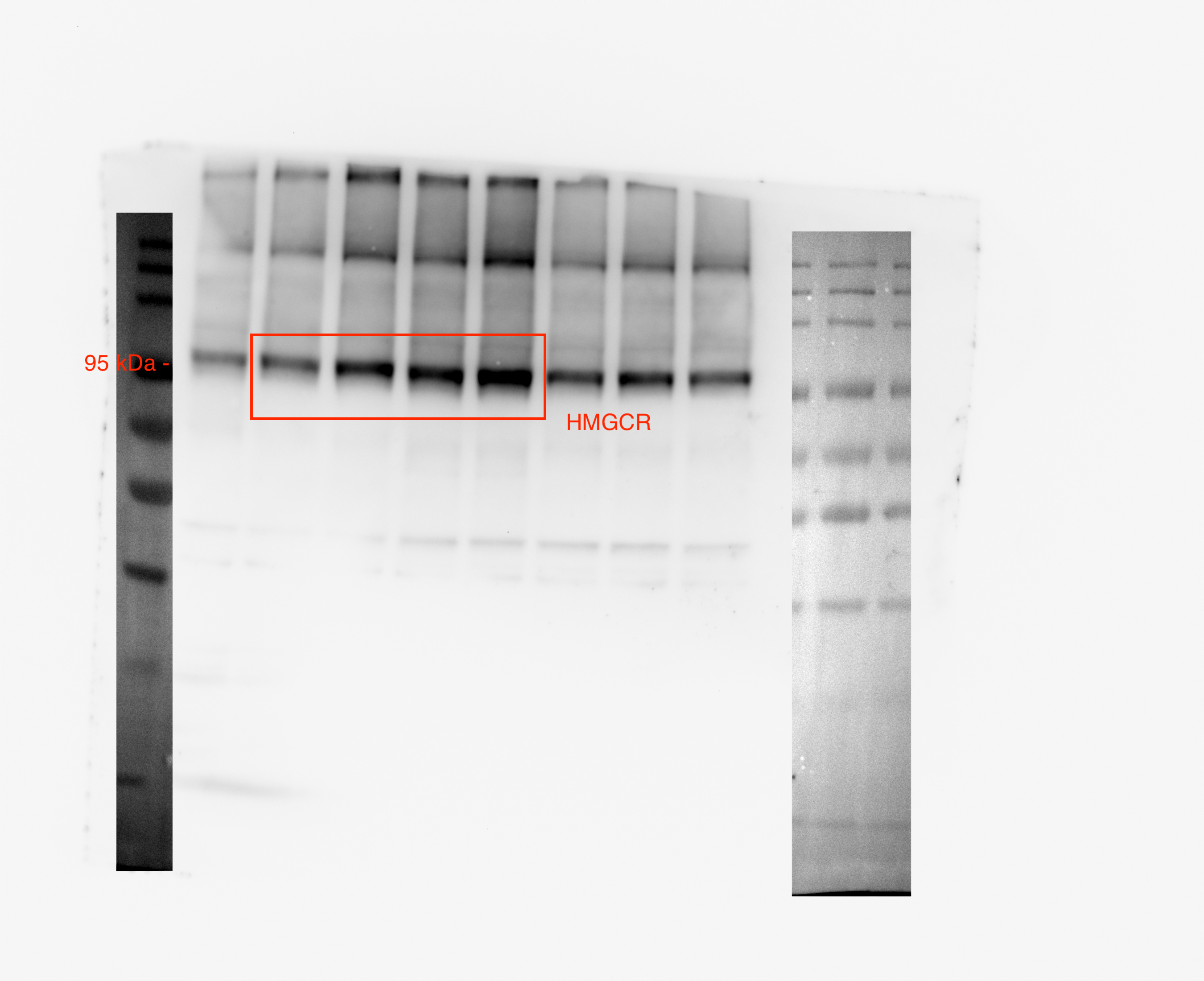

Supplement: Supplementary file 9 — Source data Fig. 5 [file 44318_2024_269_MOESM9_ESM.zip › Figure 5/5F/HMGCR.jpg]

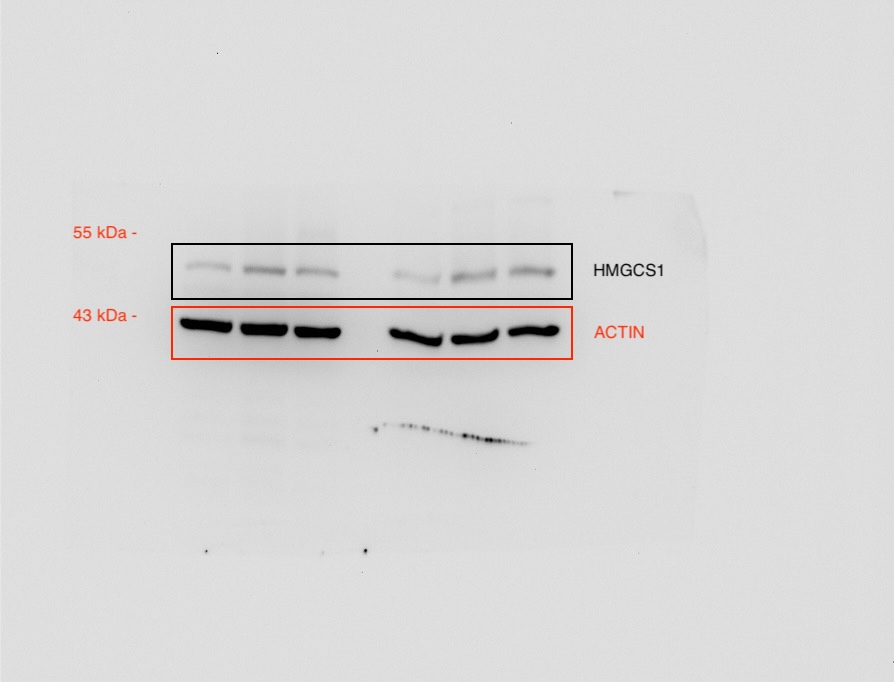

Supplement: Supplementary file 9 — Source data Fig. 5 [file 44318_2024_269_MOESM9_ESM.zip › Figure 5/5H/ACTIN.jpg]

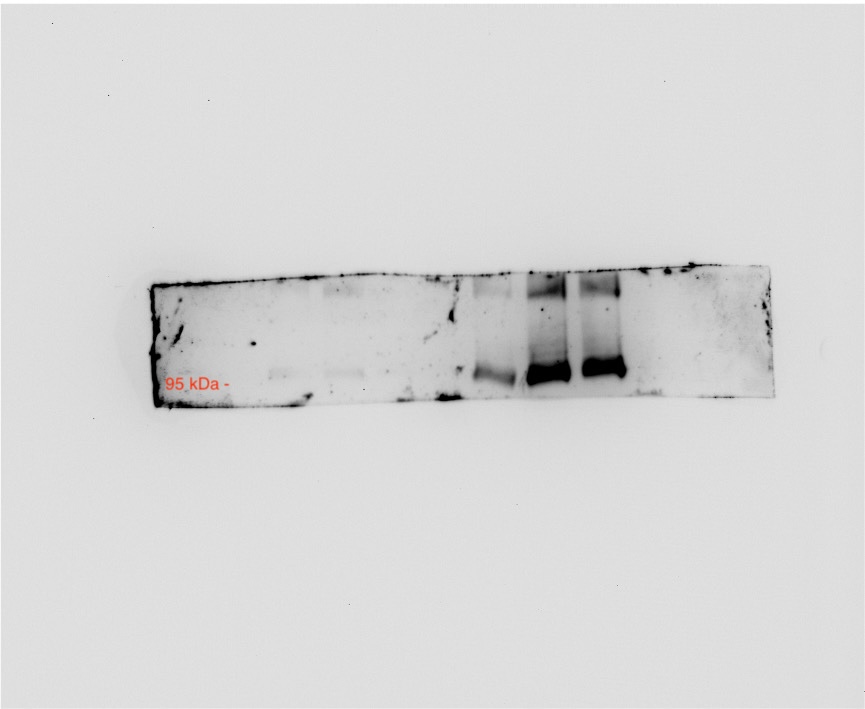

Supplement: Supplementary file 9 — Source data Fig. 5 [file 44318_2024_269_MOESM9_ESM.zip › Figure 5/5H/HMCR short.jpg]

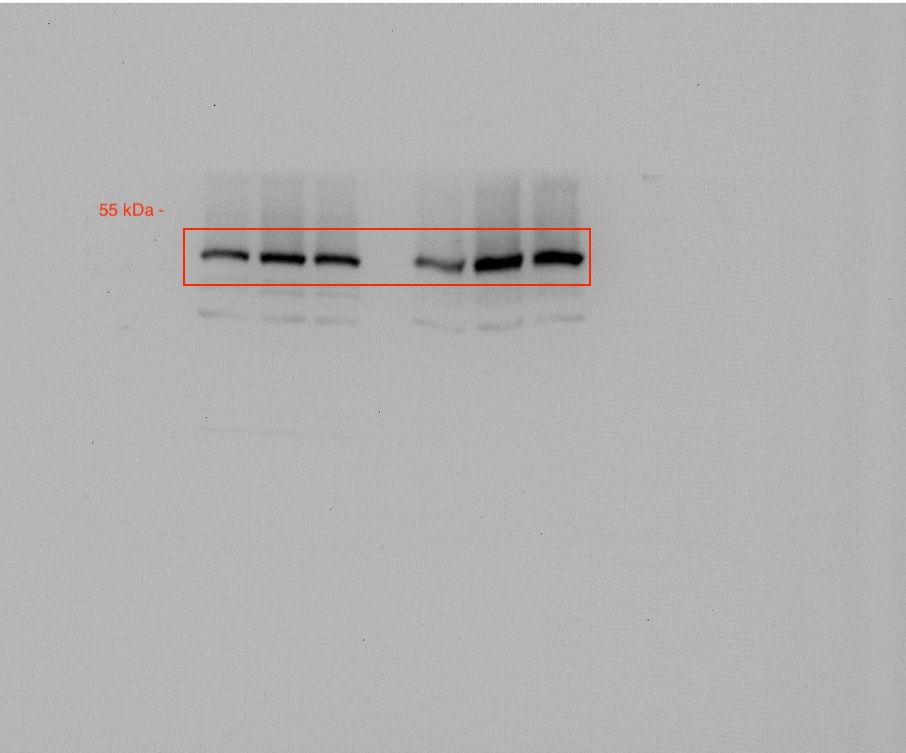

Supplement: Supplementary file 9 — Source data Fig. 5 [file 44318_2024_269_MOESM9_ESM.zip › Figure 5/5H/HMGCS1.jpg]

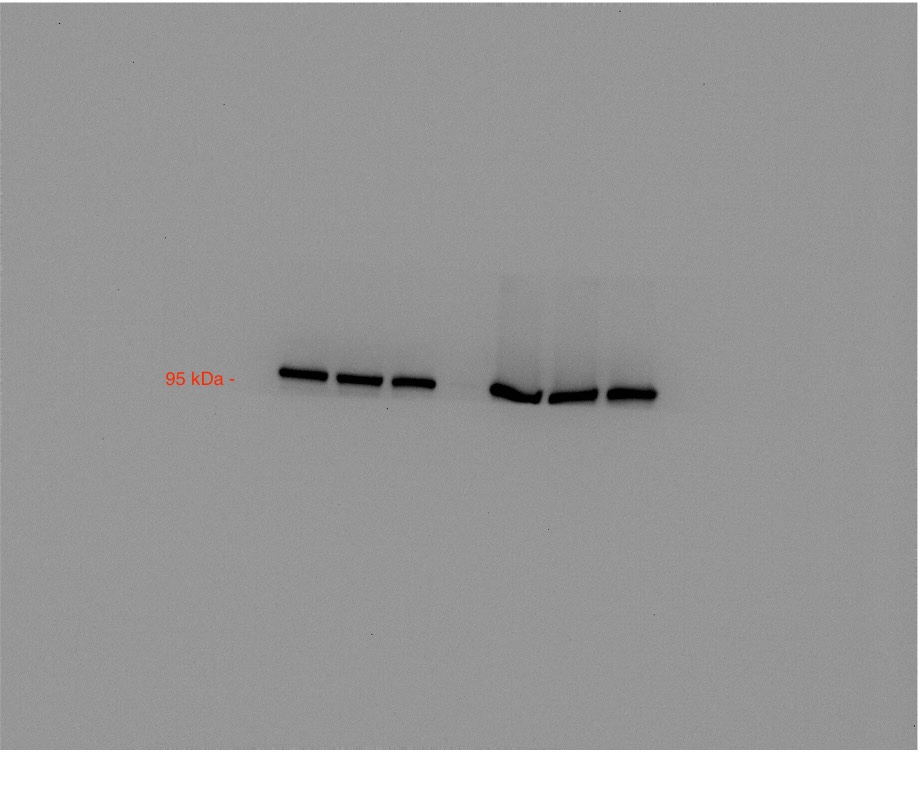

Supplement: Supplementary file 9 — Source data Fig. 5 [file 44318_2024_269_MOESM9_ESM.zip › Figure 5/5H/CNX.jpg]

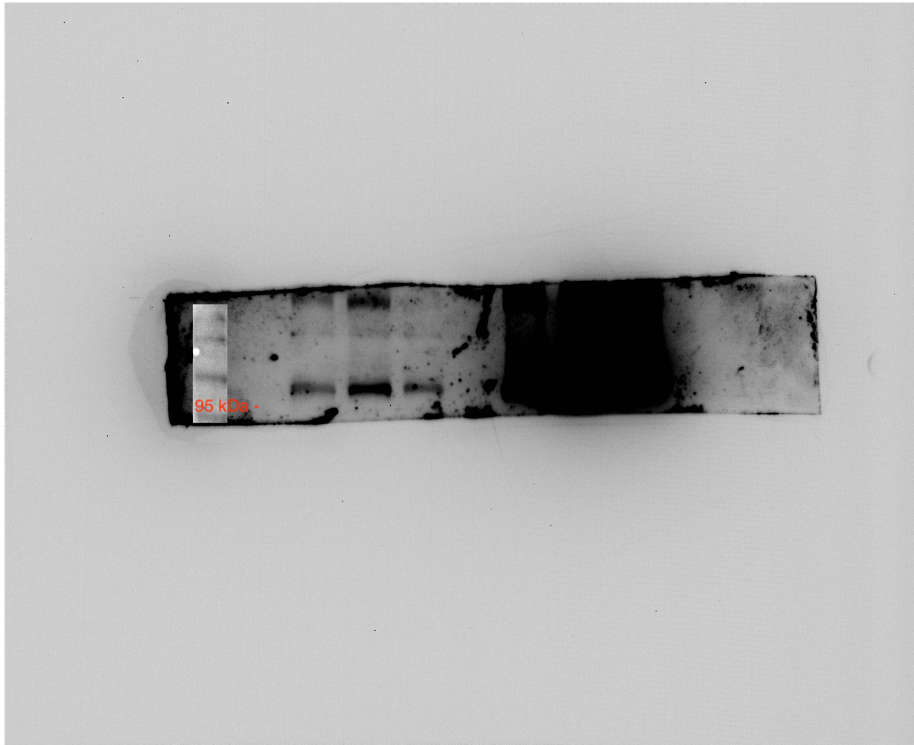

Supplement: Supplementary file 9 — Source data Fig. 5 [file 44318_2024_269_MOESM9_ESM.zip › Figure 5/5H/HMGCR high.png]

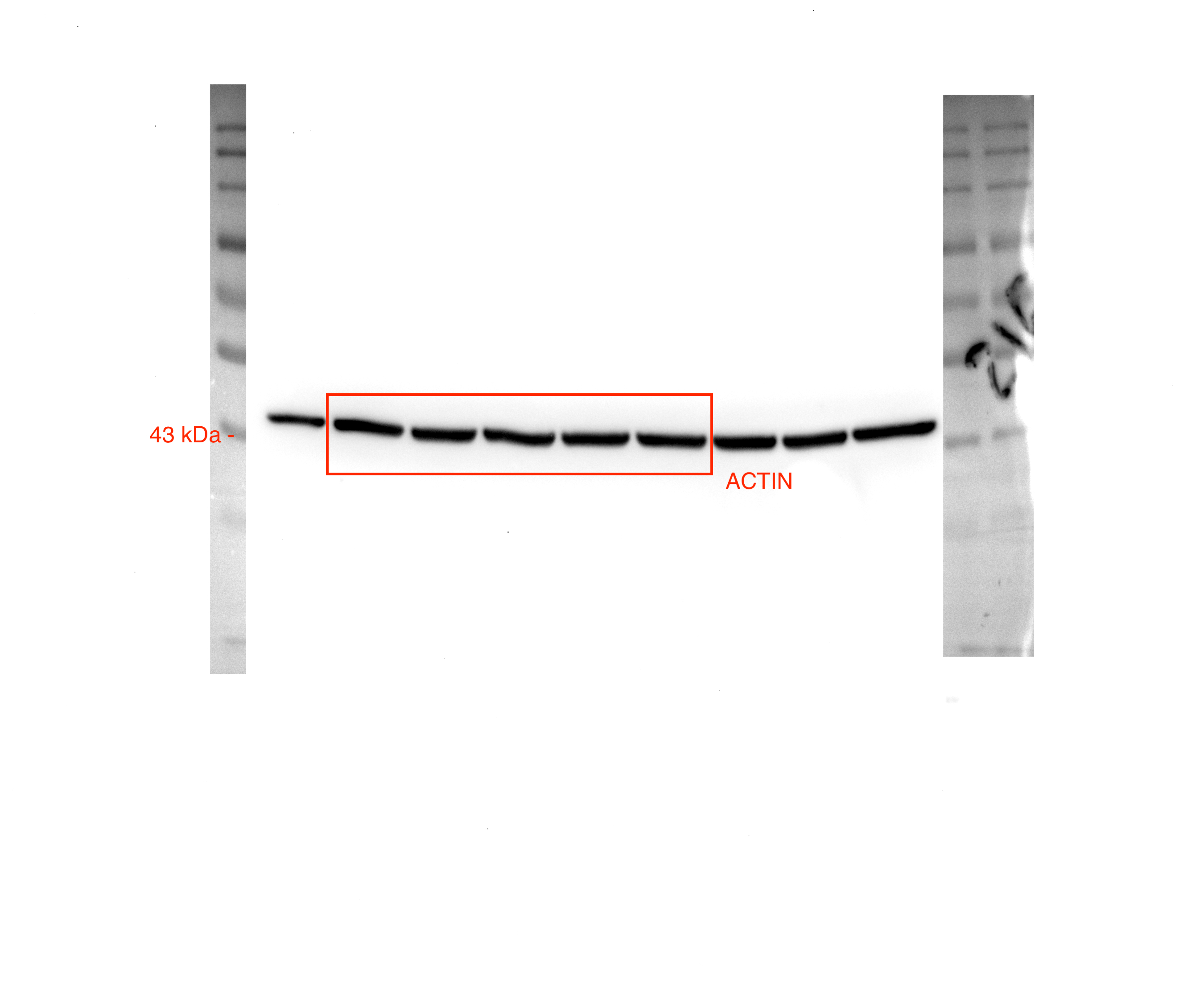

Supplement: Supplementary file 9 — Source data Fig. 5 [file 44318_2024_269_MOESM9_ESM.zip › Figure 5/5E/ACTIN.jpg]

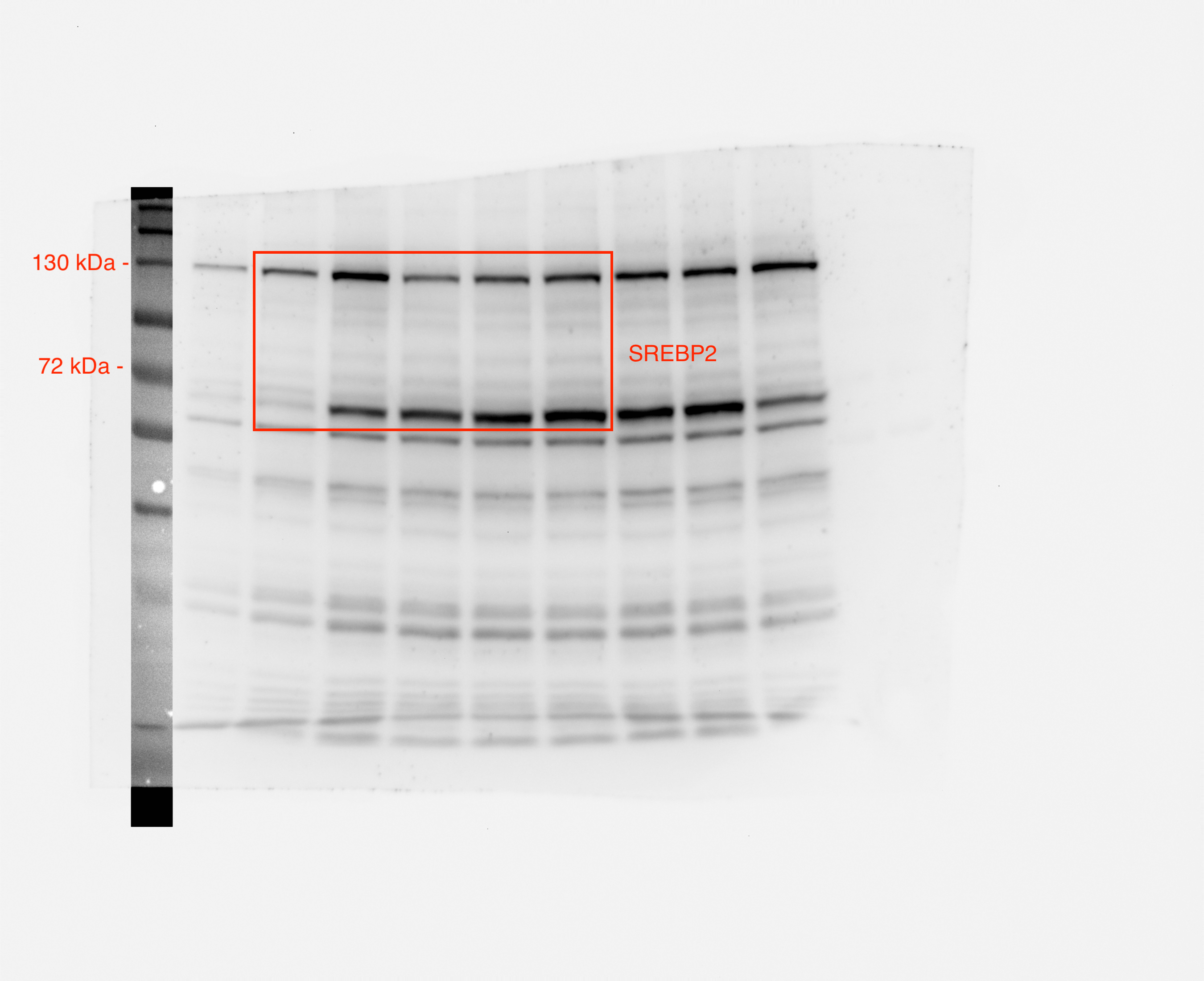

Supplement: Supplementary file 9 — Source data Fig. 5 [file 44318_2024_269_MOESM9_ESM.zip › Figure 5/5E/SREBP2.jpg]

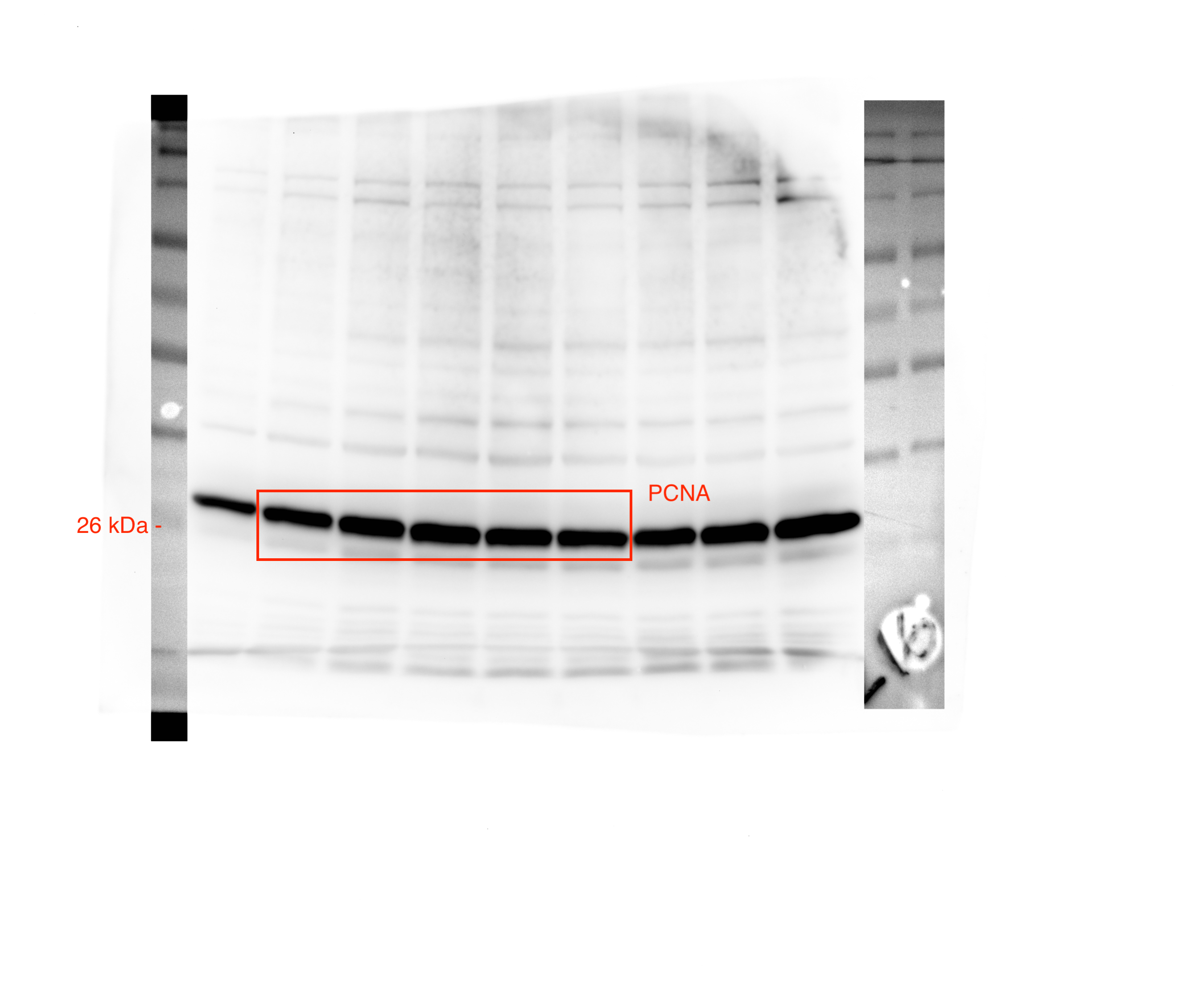

Supplement: Supplementary file 9 — Source data Fig. 5 [file 44318_2024_269_MOESM9_ESM.zip › Figure 5/5E/PCNA.jpg]

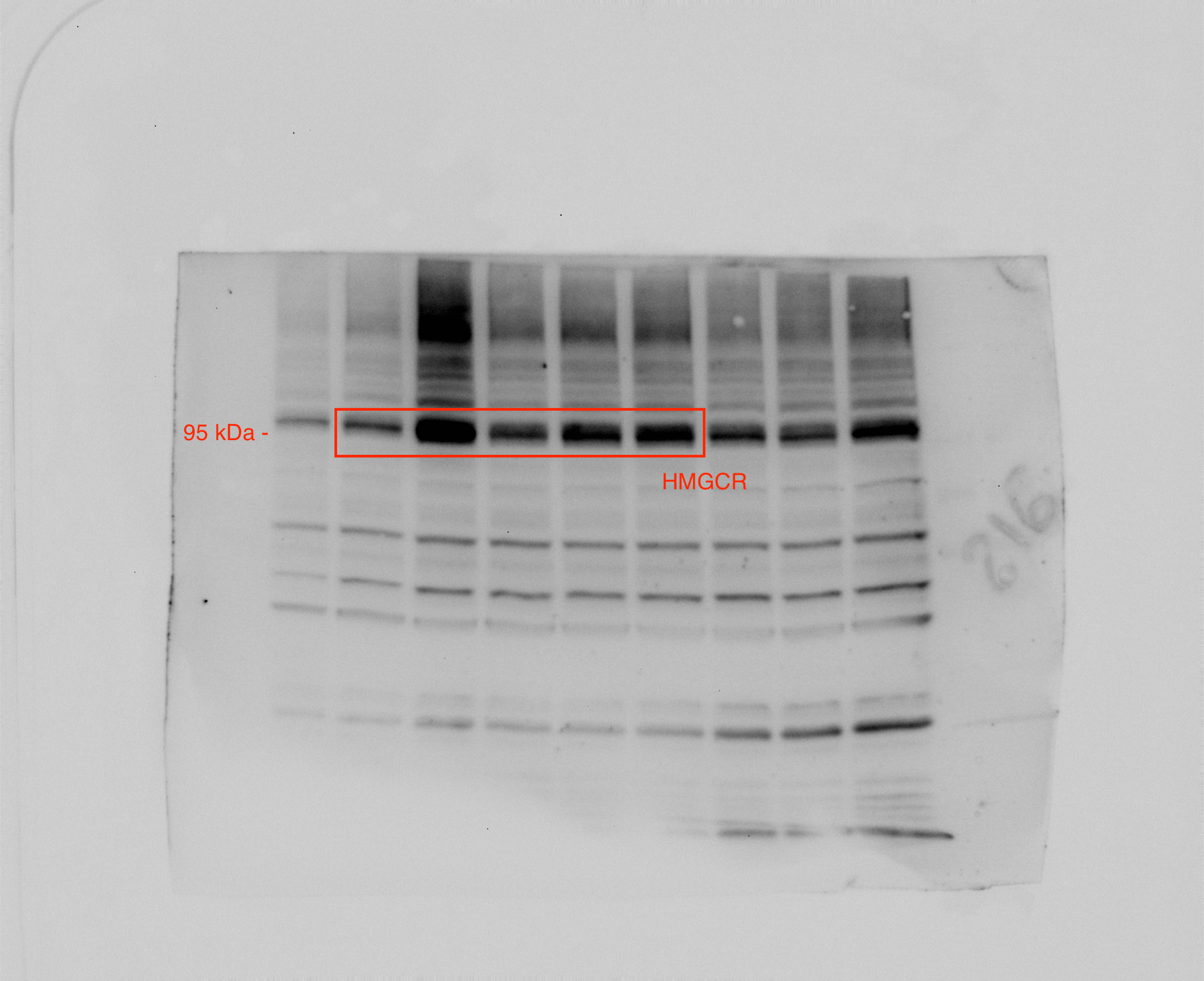

Supplement: Supplementary file 9 — Source data Fig. 5 [file 44318_2024_269_MOESM9_ESM.zip › Figure 5/5E/HMGCR.jpg]

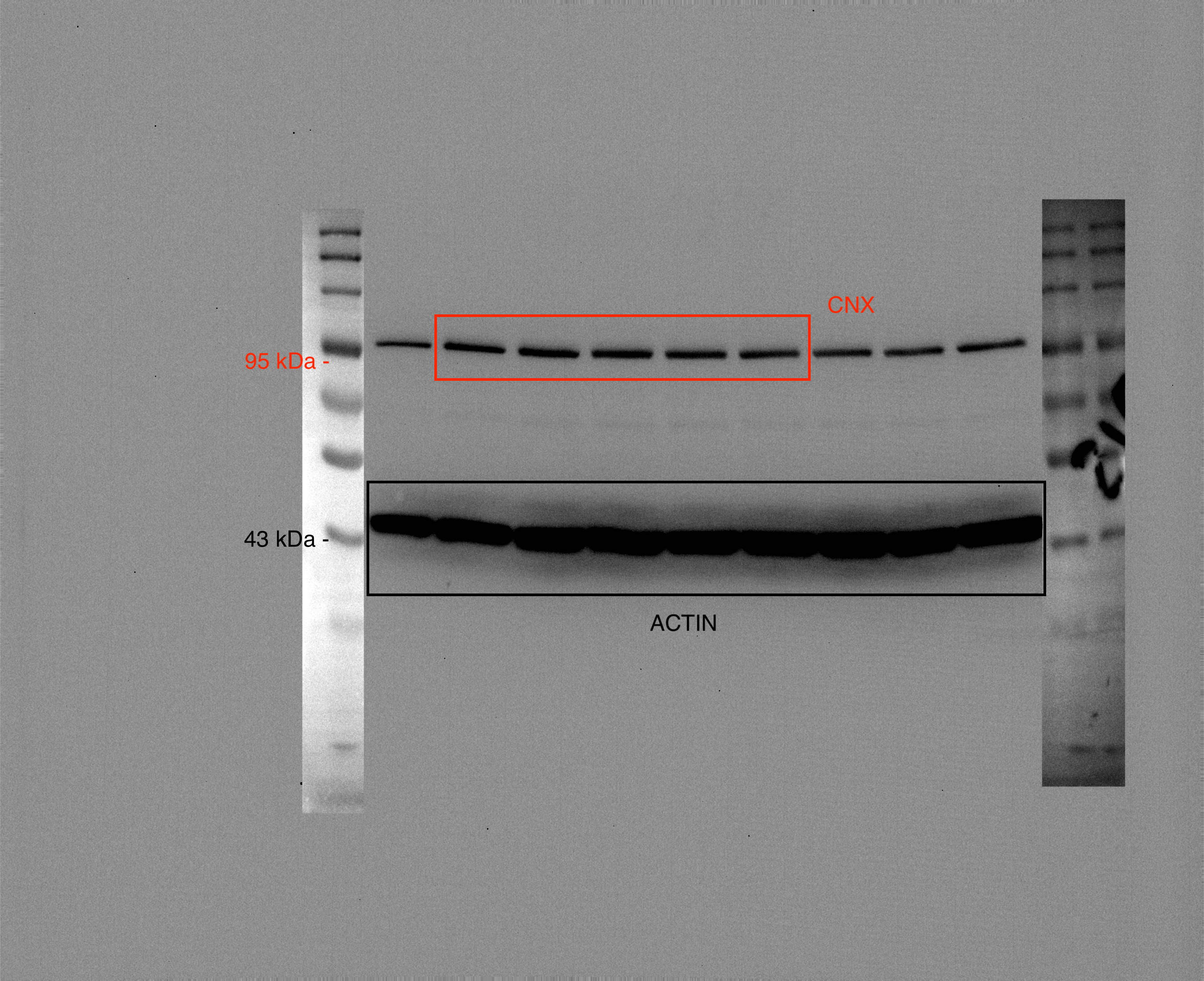

Supplement: Supplementary file 9 — Source data Fig. 5 [file 44318_2024_269_MOESM9_ESM.zip › Figure 5/5E/CNX.jpg]

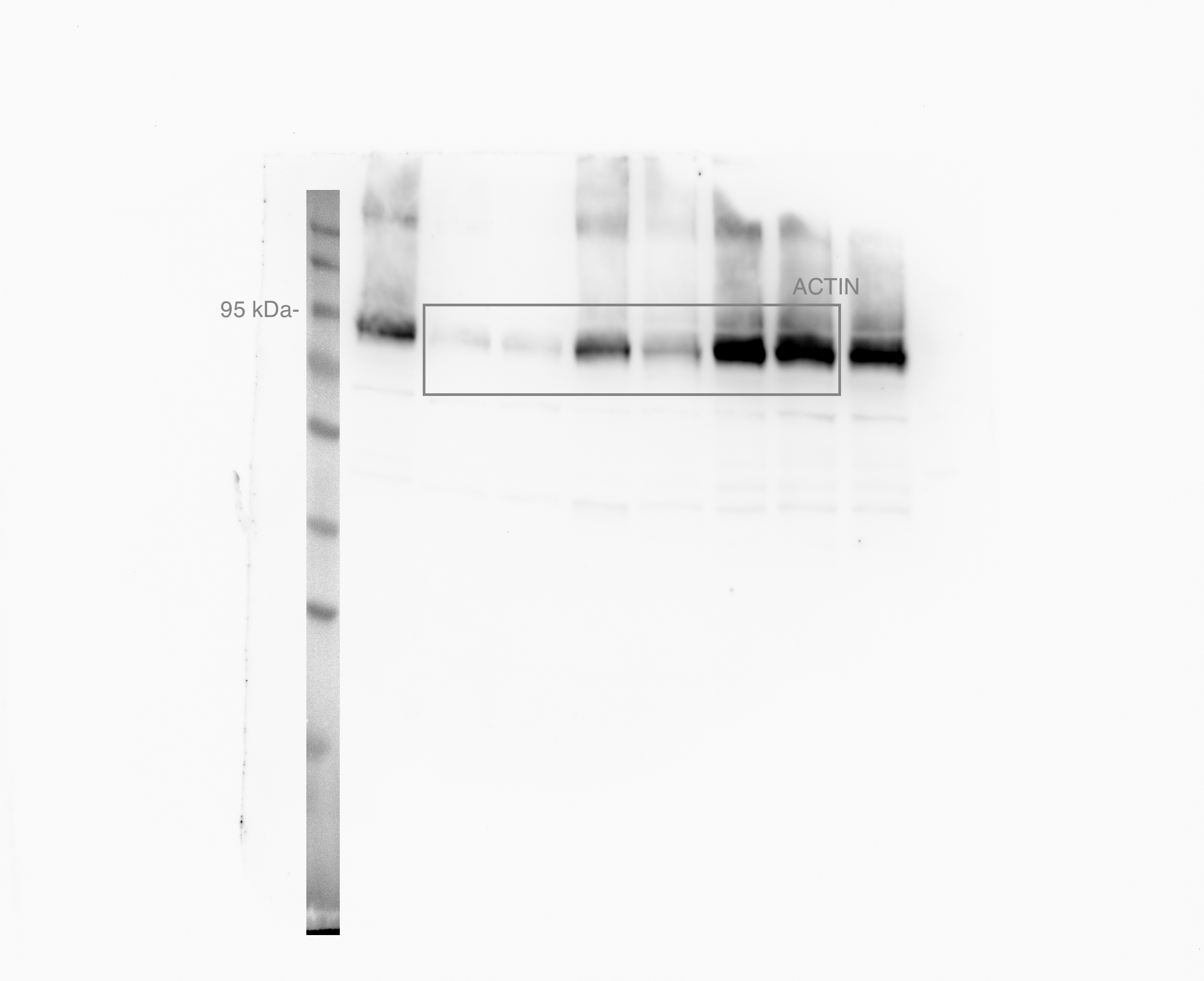

Supplement: Supplementary file 9 — Source data Fig. 5 [file 44318_2024_269_MOESM9_ESM.zip › Figure 5/5B/HMGCR.jpg]

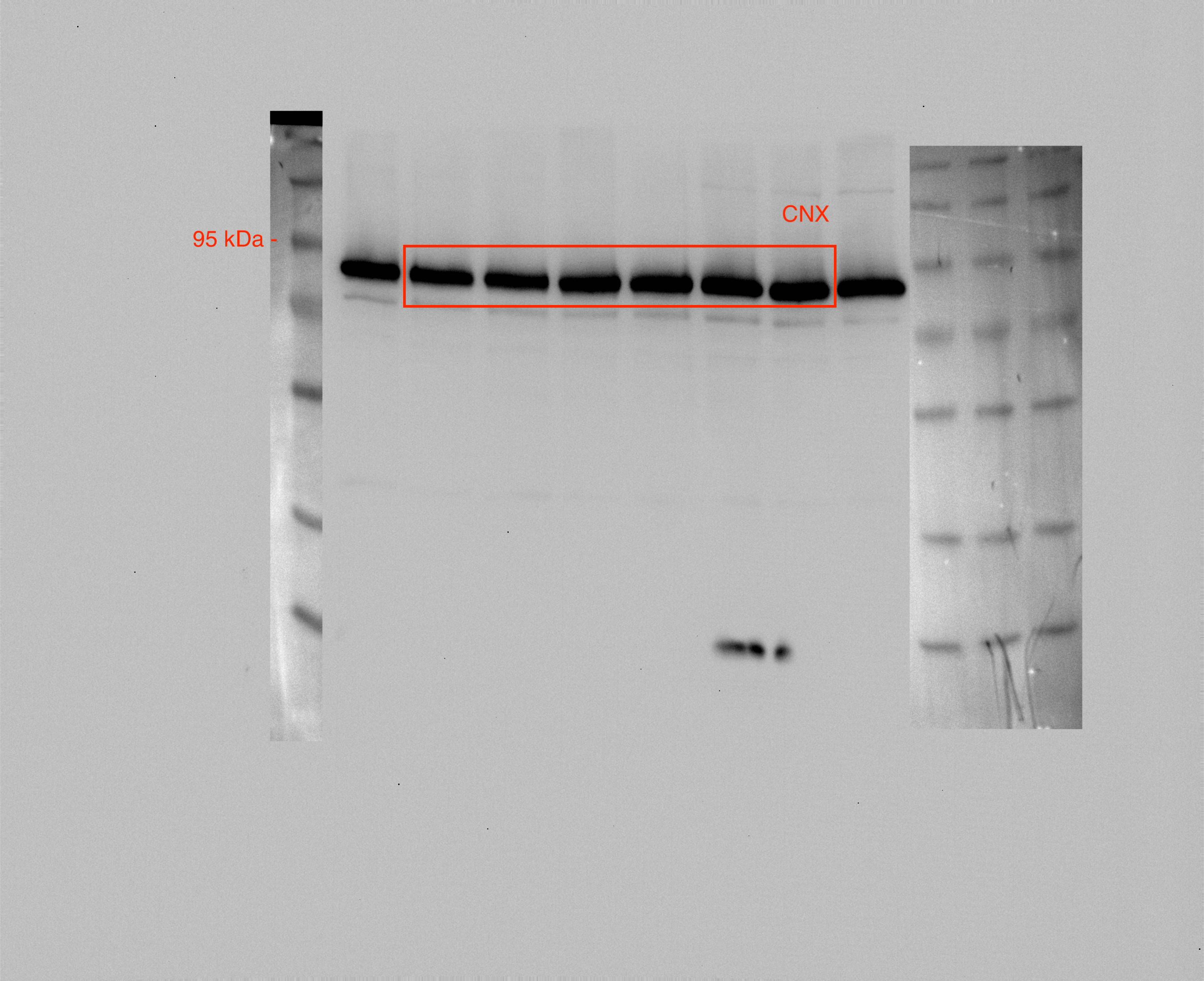

Supplement: Supplementary file 9 — Source data Fig. 5 [file 44318_2024_269_MOESM9_ESM.zip › Figure 5/5B/CNX.tiff]

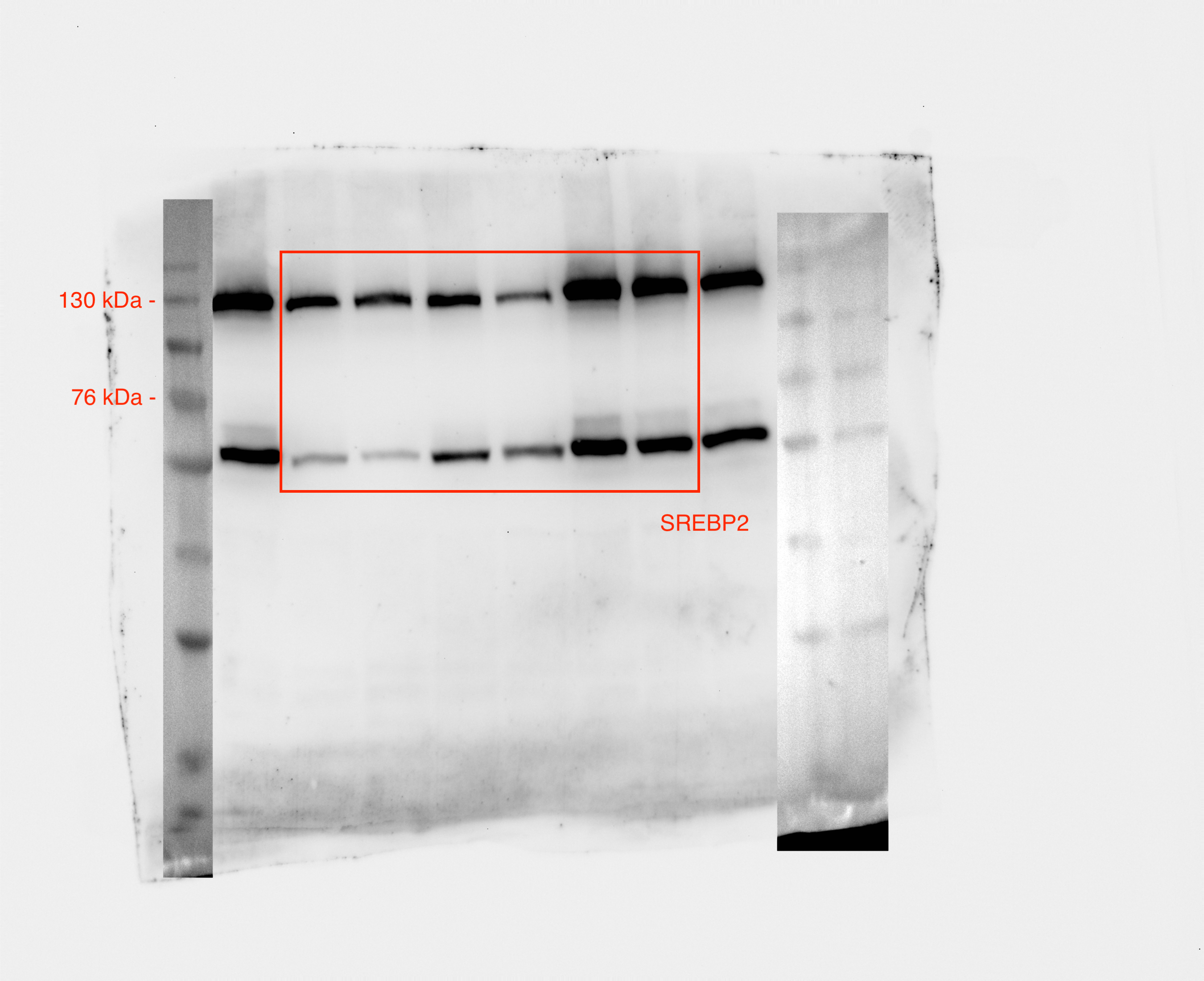

Supplement: Supplementary file 9 — Source data Fig. 5 [file 44318_2024_269_MOESM9_ESM.zip › Figure 5/5B/SREBP2.tiff]

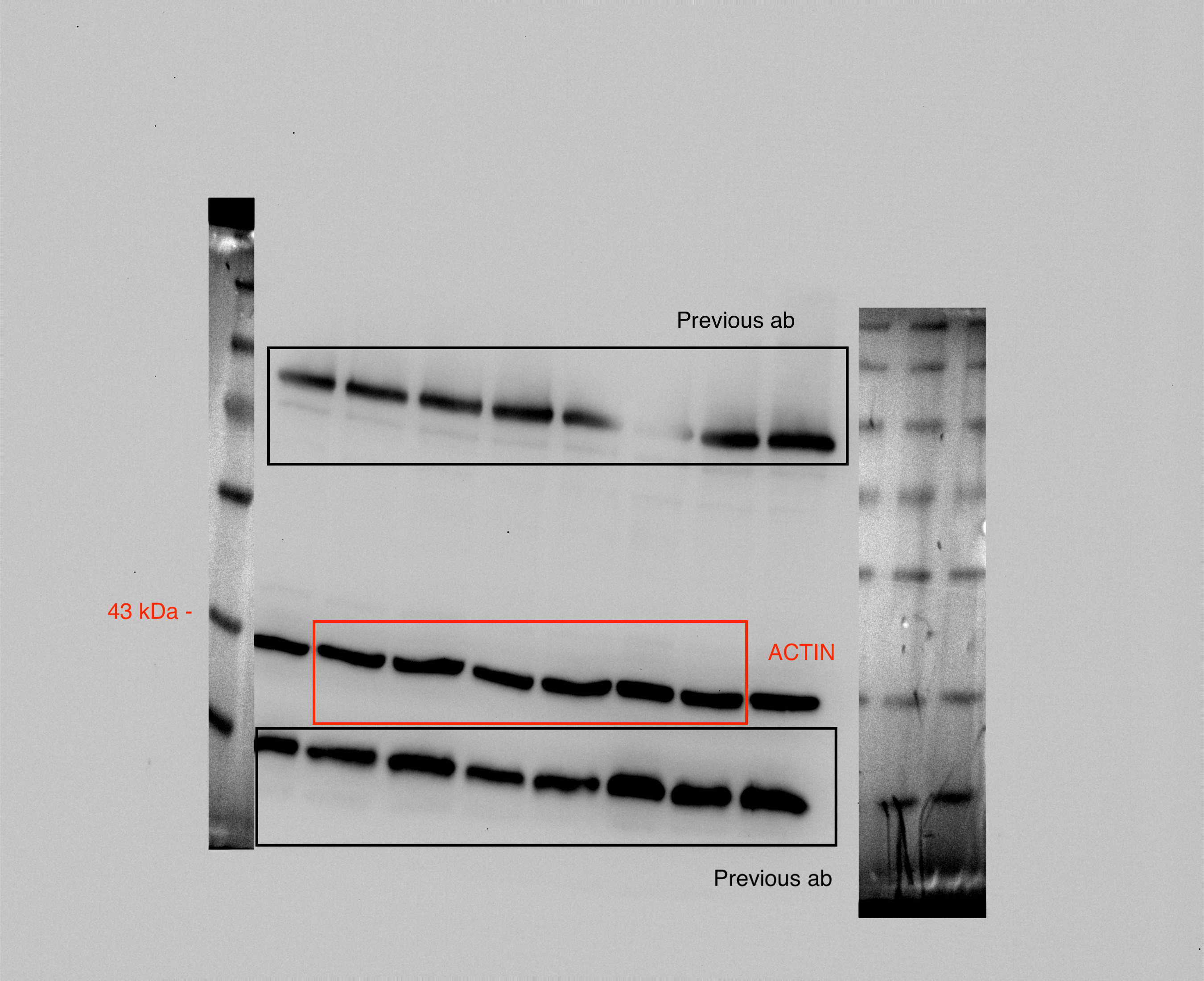

Supplement: Supplementary file 9 — Source data Fig. 5 [file 44318_2024_269_MOESM9_ESM.zip › Figure 5/5B/ACTIN.tiff]

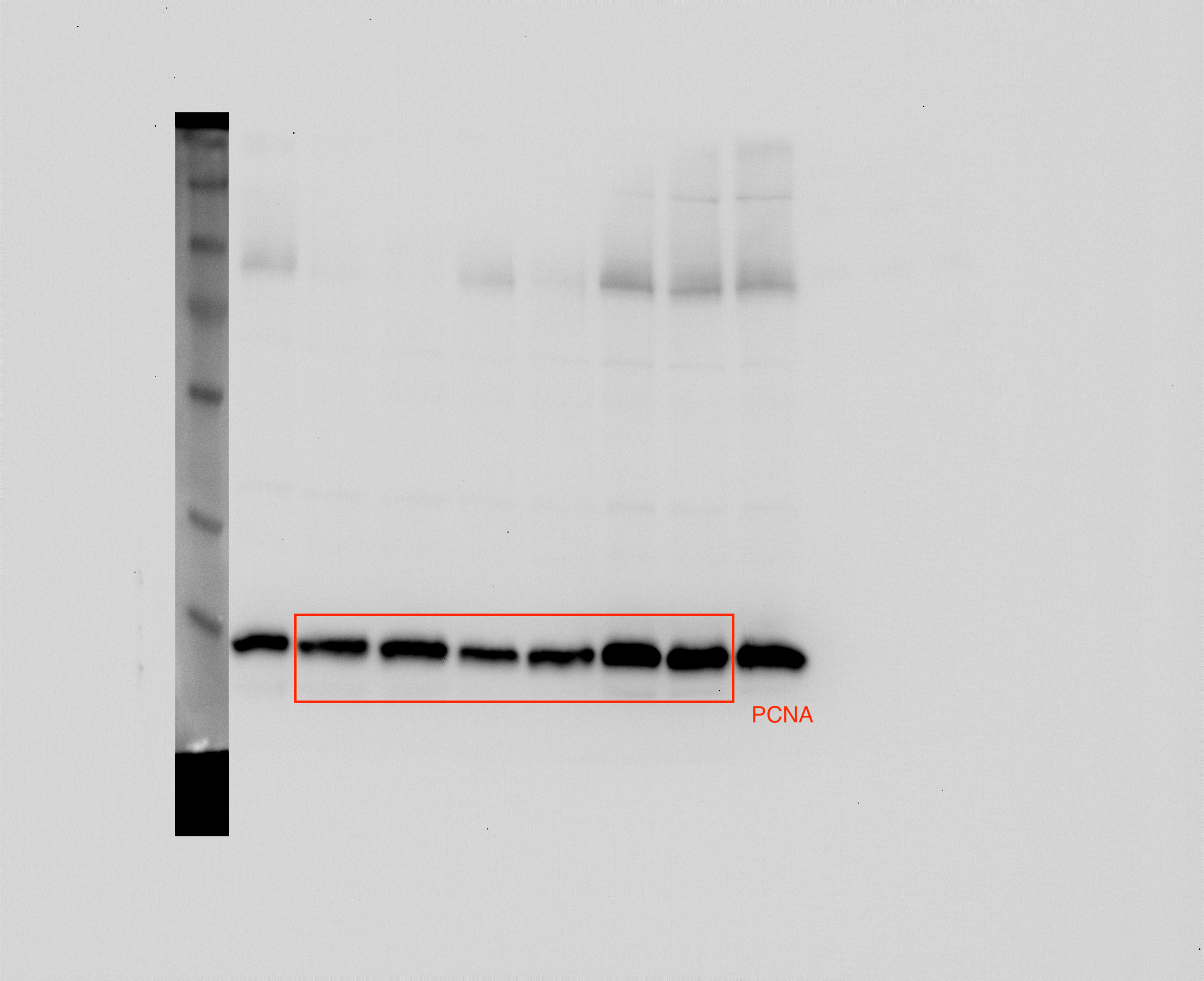

Supplement: Supplementary file 9 — Source data Fig. 5 [file 44318_2024_269_MOESM9_ESM.zip › Figure 5/5B/PCNA.tiff]

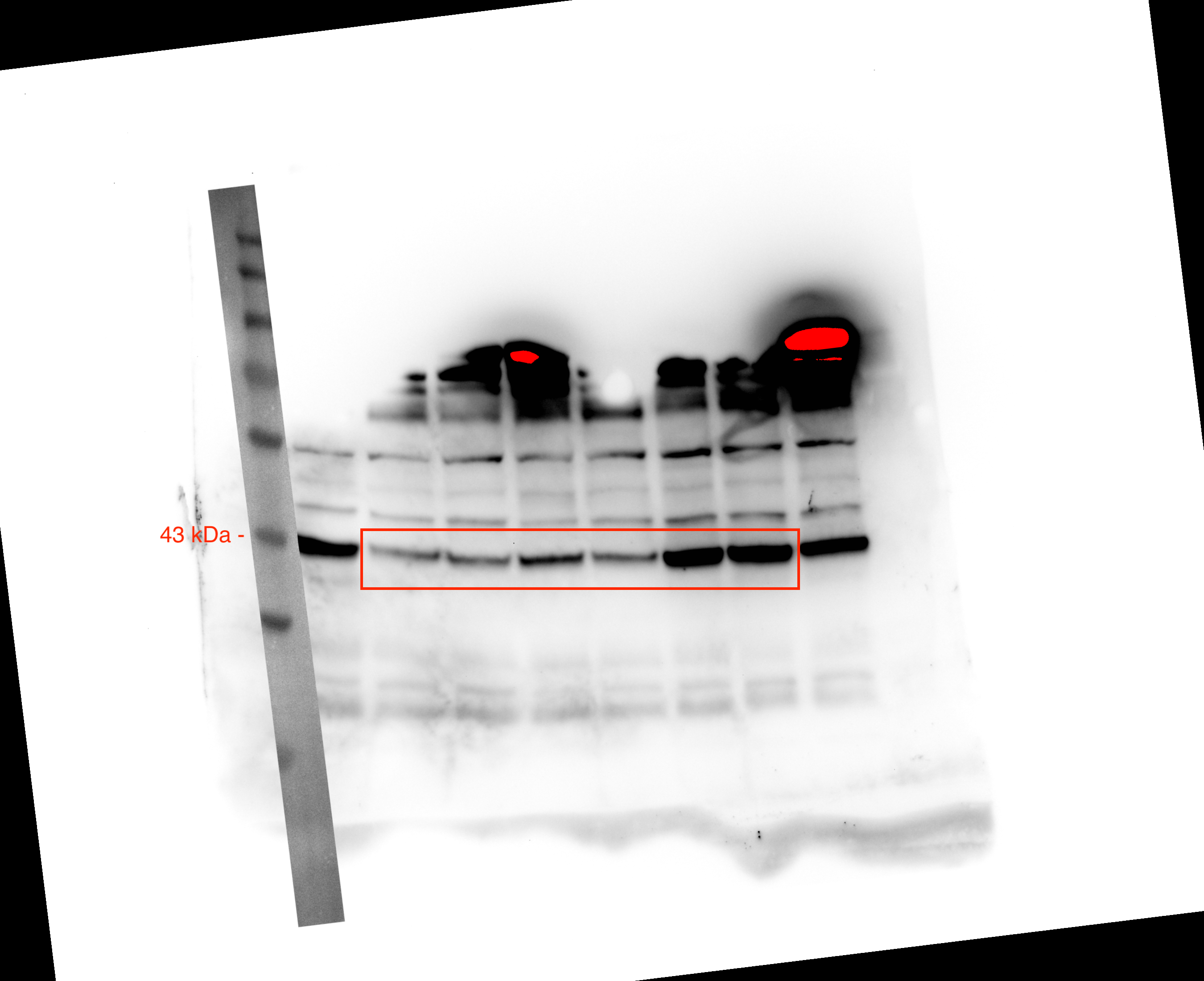

Supplement: Supplementary file 9 — Source data Fig. 5 [file 44318_2024_269_MOESM9_ESM.zip › Figure 5/5B/FDFT1.tif]

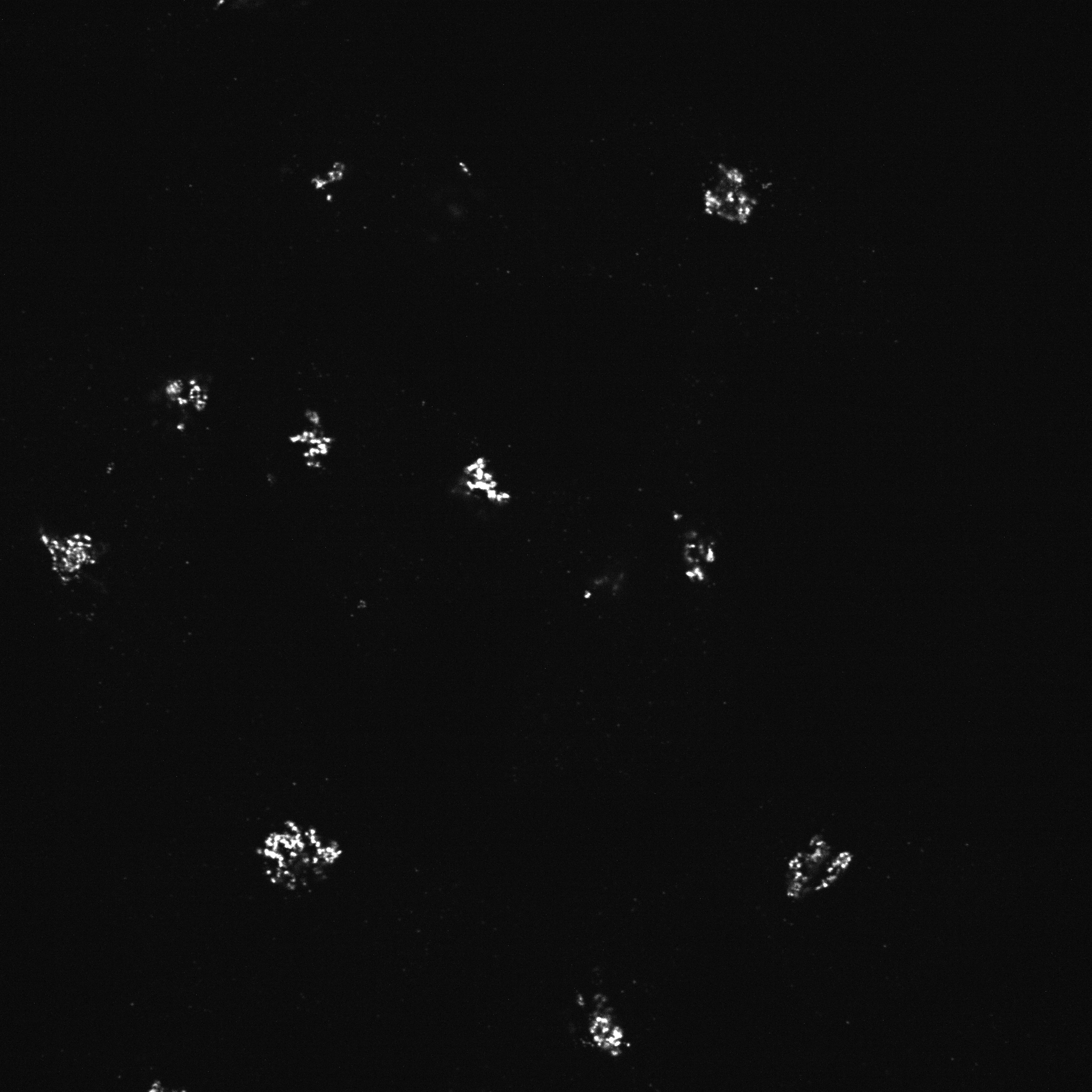

Supplement: Supplementary file 9 — Source data Fig. 5 [file 44318_2024_269_MOESM9_ESM.zip › Figure 5/5C-D/13915_+aKG_GOLGA1.tif]

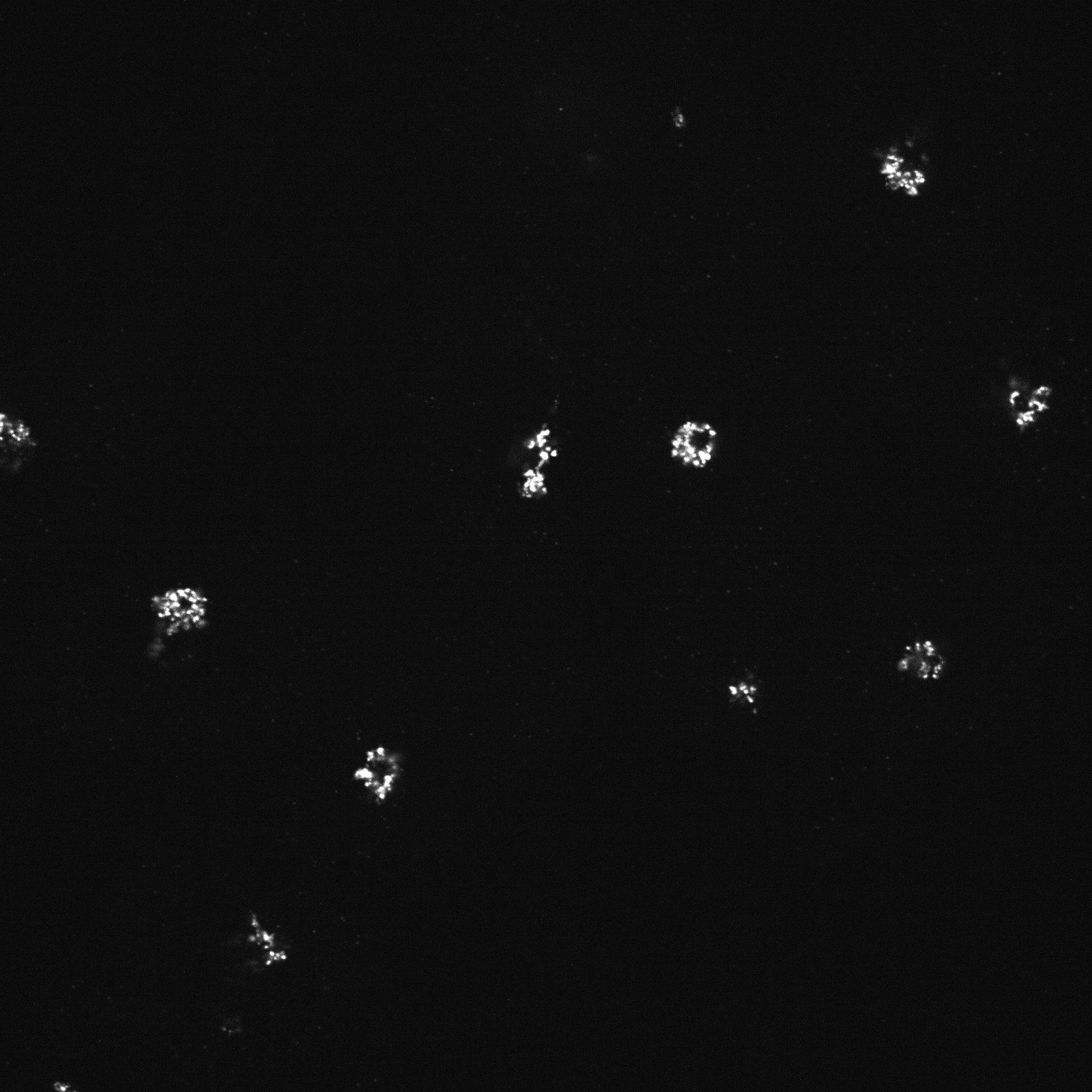

Supplement: Supplementary file 9 — Source data Fig. 5 [file 44318_2024_269_MOESM9_ESM.zip › Figure 5/5C-D/13916_+glut_merge.tif]

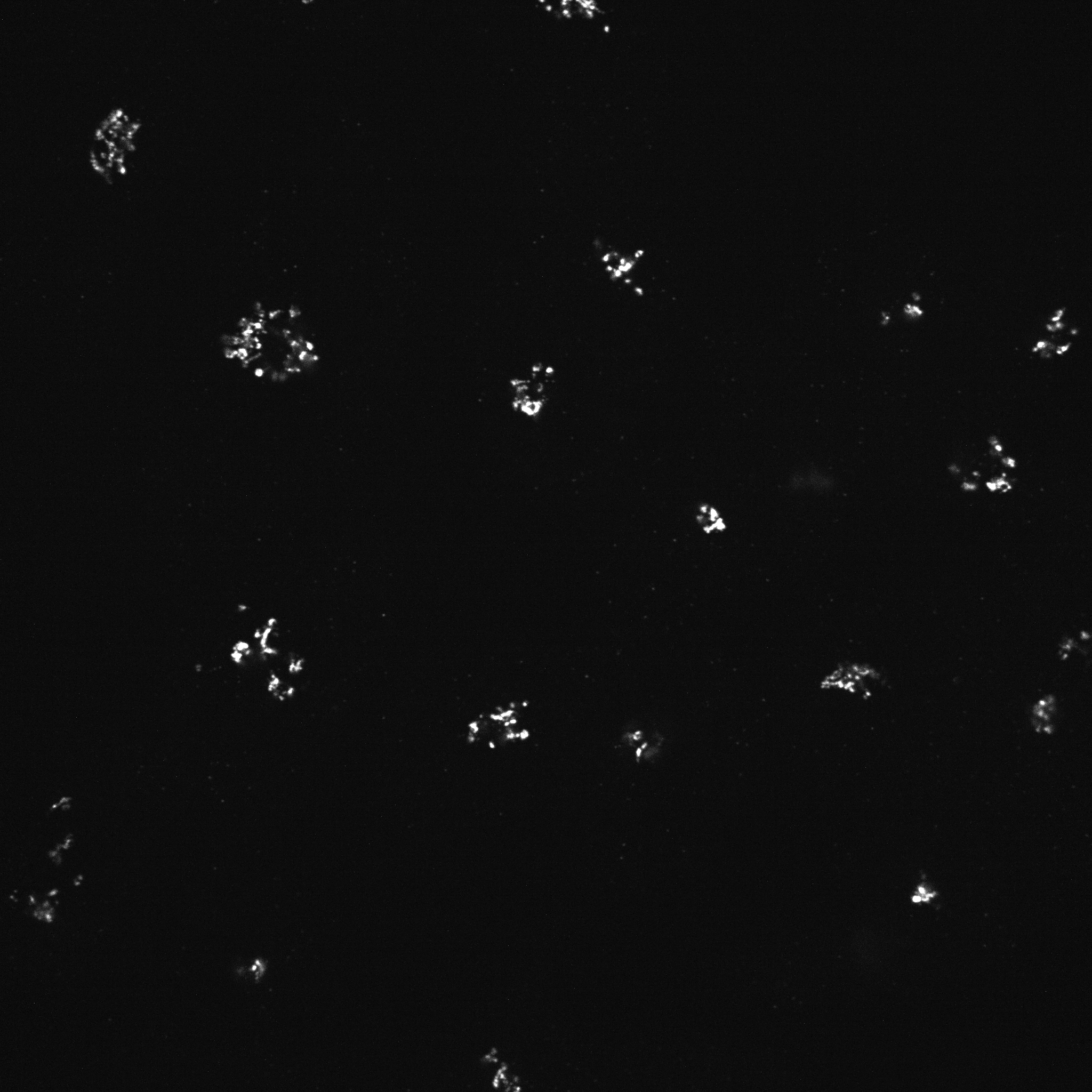

Supplement: Supplementary file 9 — Source data Fig. 5 [file 44318_2024_269_MOESM9_ESM.zip › Figure 5/5C-D/13912_noglut_merge.tif]

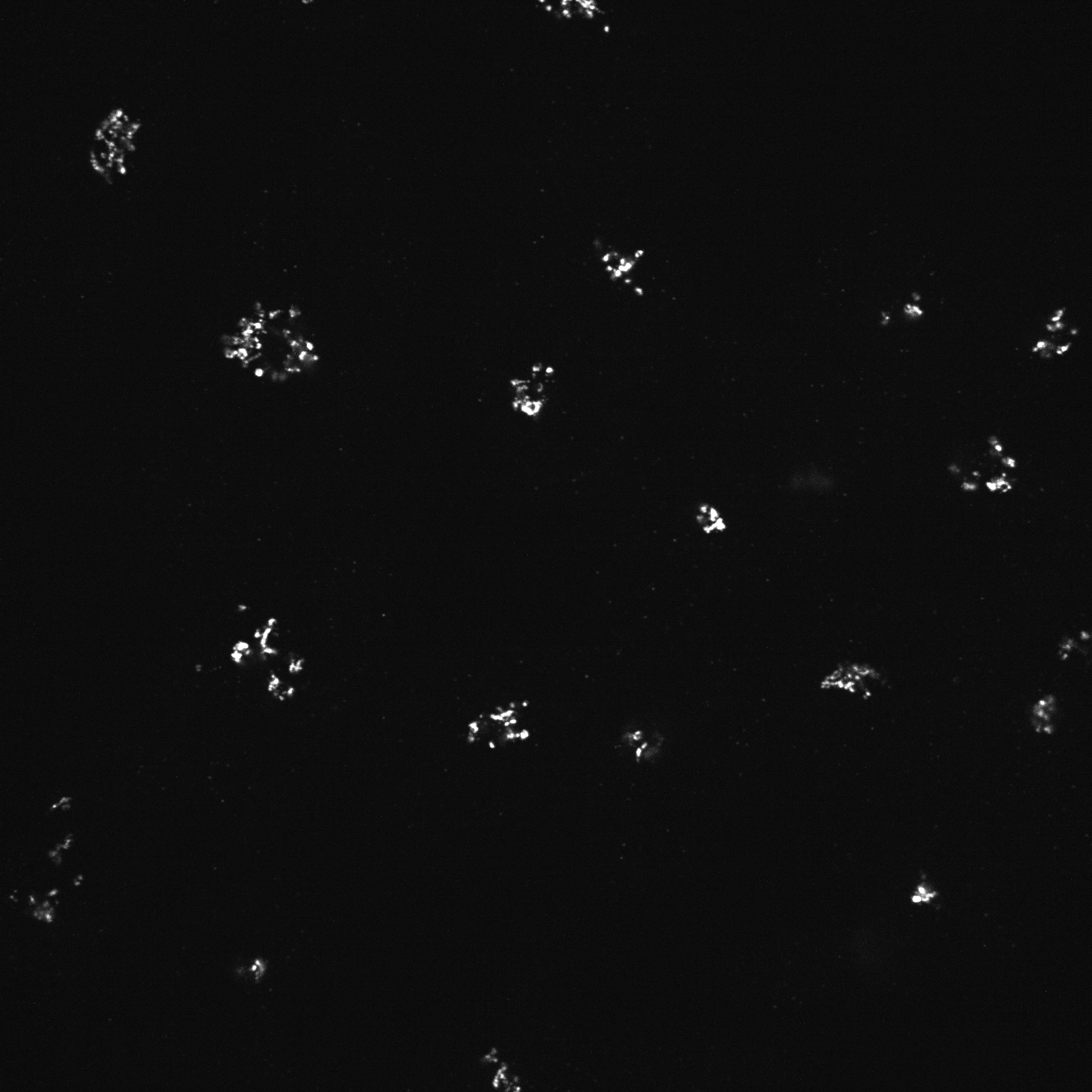

Supplement: Supplementary file 9 — Source data Fig. 5 [file 44318_2024_269_MOESM9_ESM.zip › Figure 5/5C-D/13912_noglut_GOLGA1.tif]

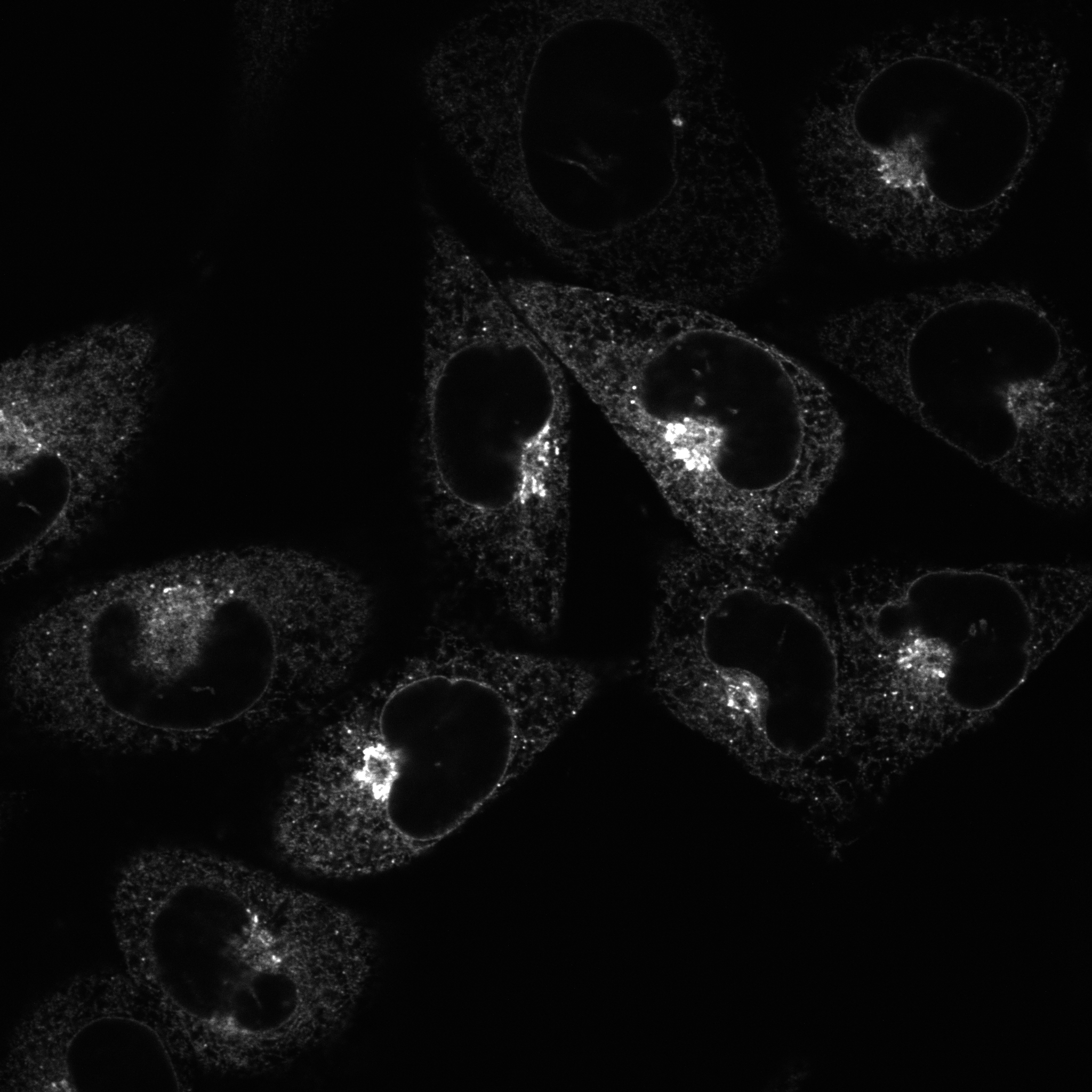

Supplement: Supplementary file 9 — Source data Fig. 5 [file 44318_2024_269_MOESM9_ESM.zip › Figure 5/5C-D/13916_+glut_scap.tif]

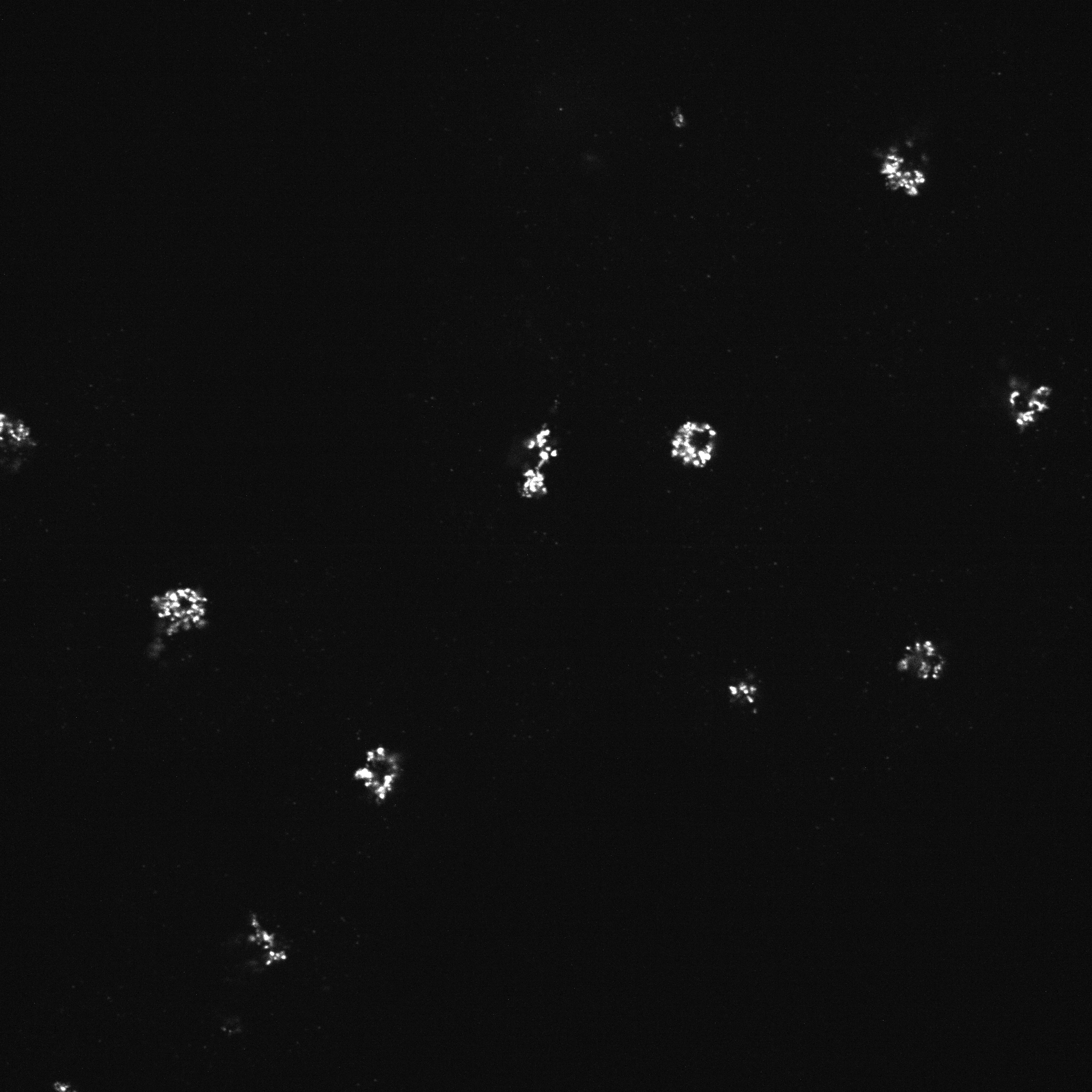

Supplement: Supplementary file 9 — Source data Fig. 5 [file 44318_2024_269_MOESM9_ESM.zip › Figure 5/5C-D/13916_+glut_GOLGA1.tif]

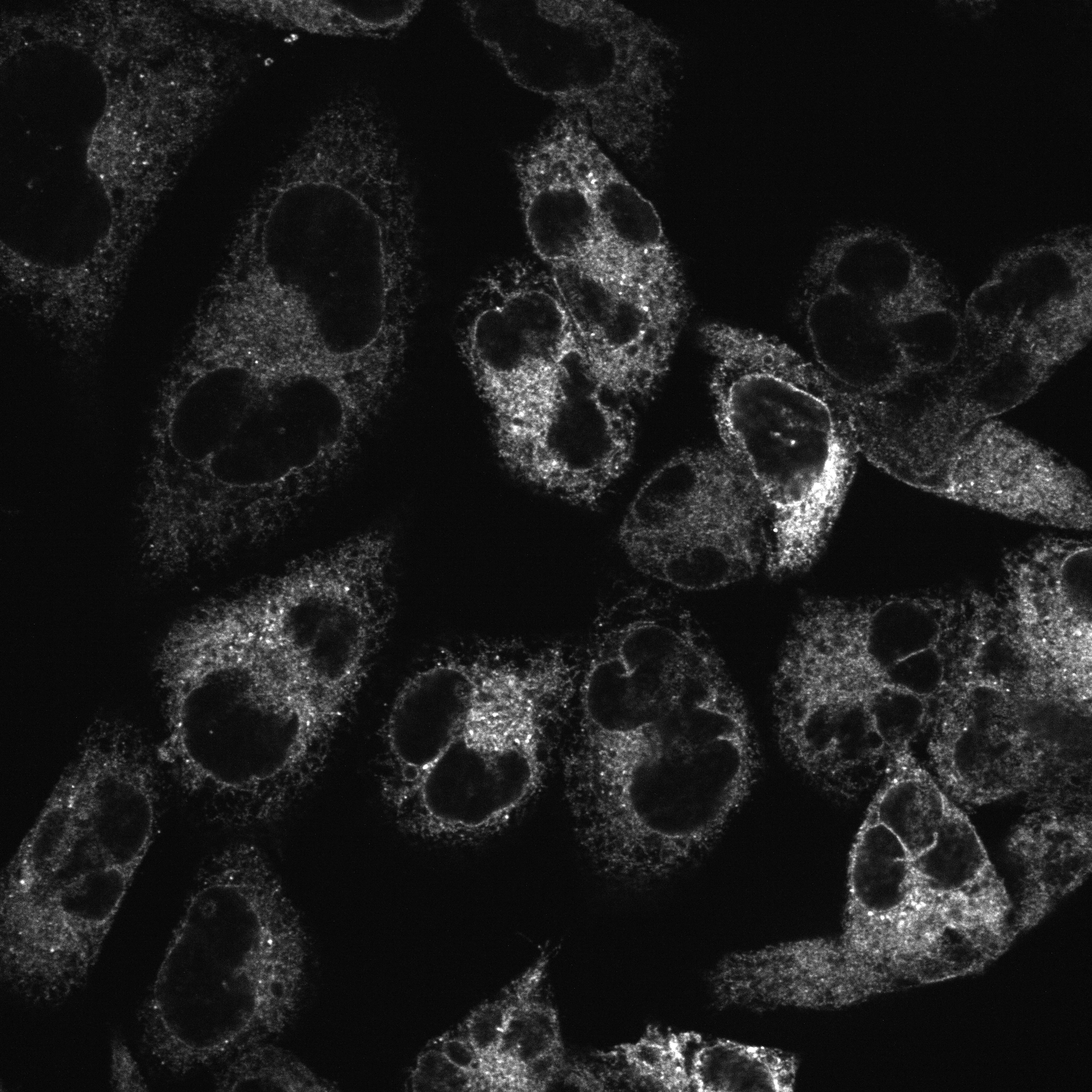

Supplement: Supplementary file 9 — Source data Fig. 5 [file 44318_2024_269_MOESM9_ESM.zip › Figure 5/5C-D/13912_noglut_scap.tif]

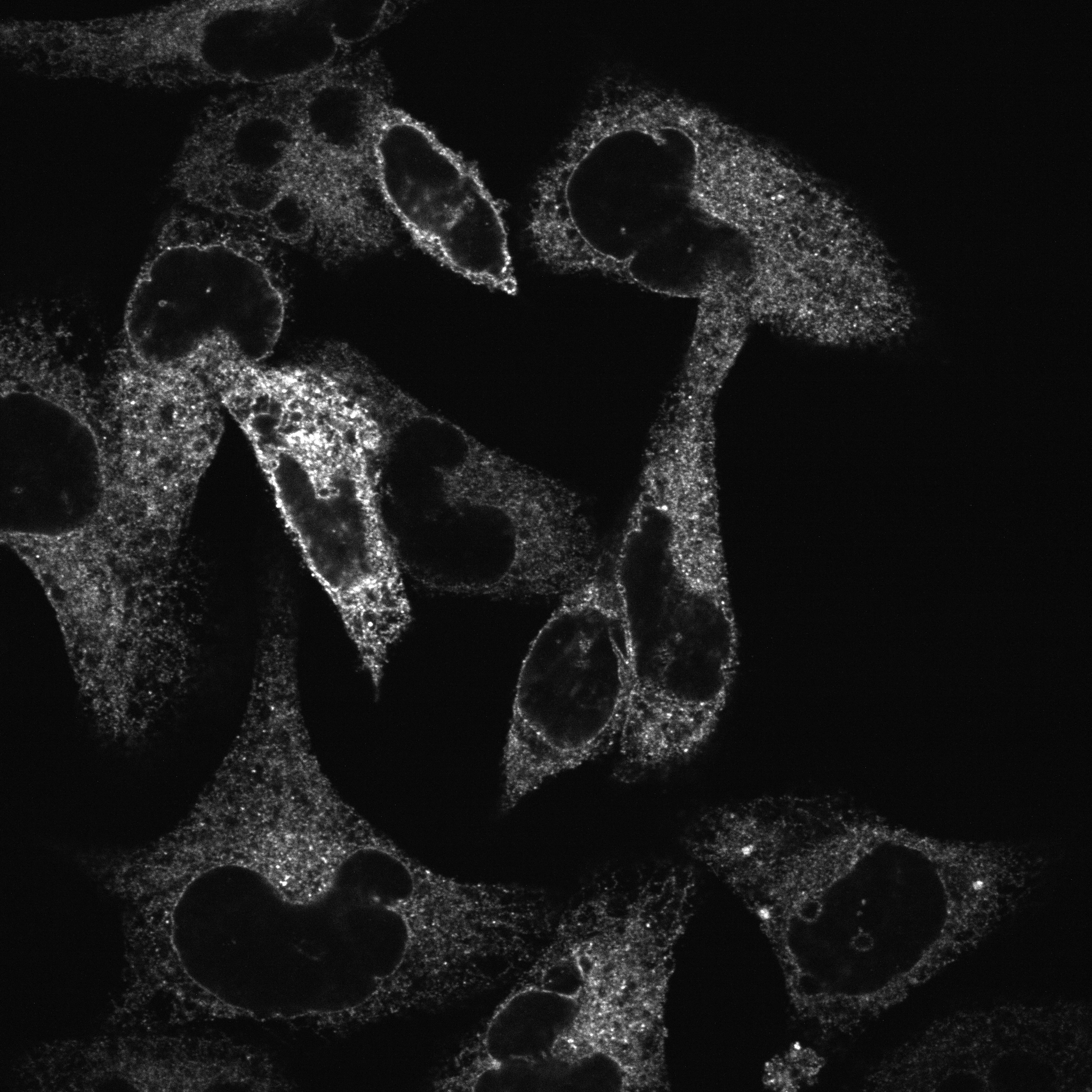

Supplement: Supplementary file 9 — Source data Fig. 5 [file 44318_2024_269_MOESM9_ESM.zip › Figure 5/5C-D/13915_+aKG_scap.tif]

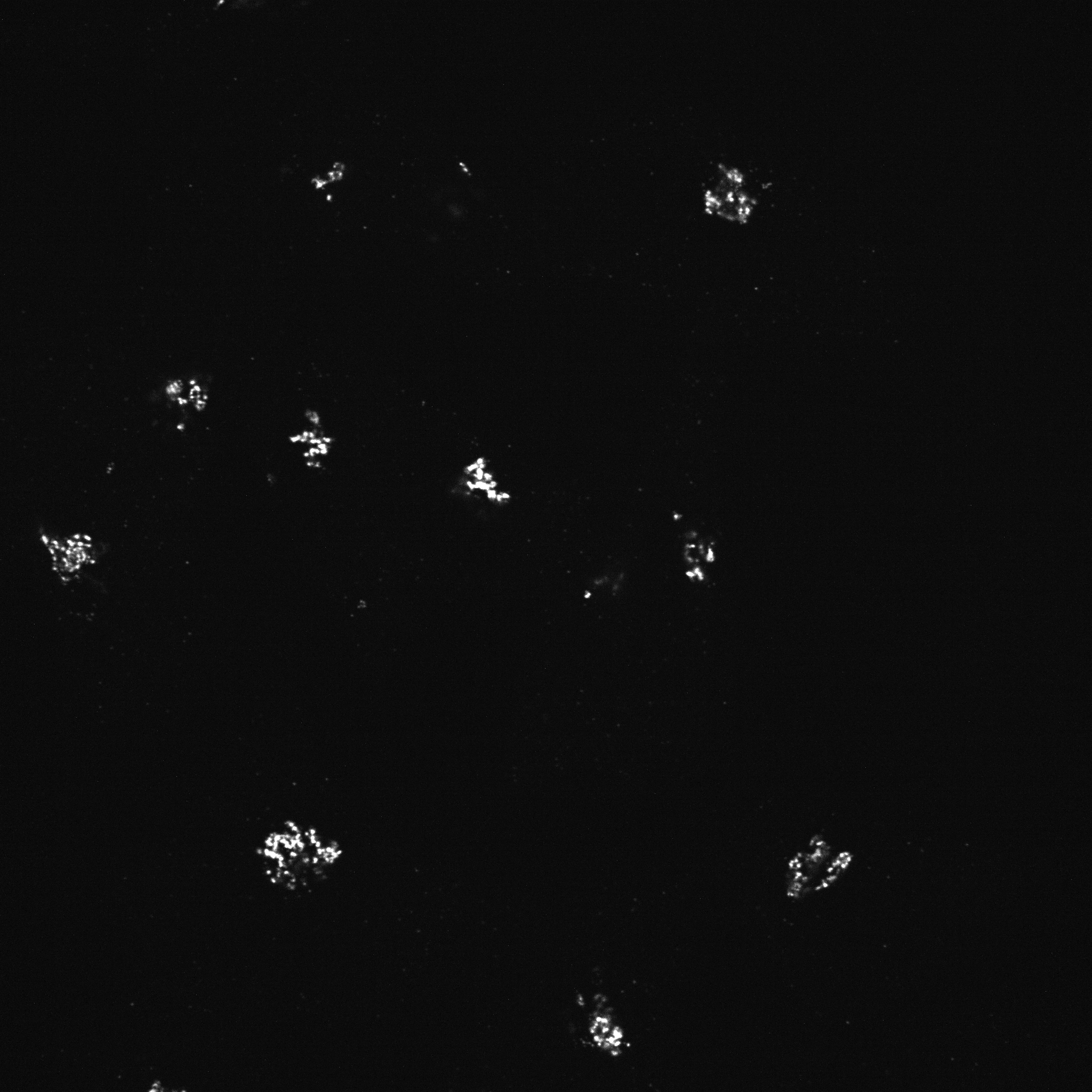

Supplement: Supplementary file 9 — Source data Fig. 5 [file 44318_2024_269_MOESM9_ESM.zip › Figure 5/5C-D/13915_+aKG_merge.tif]

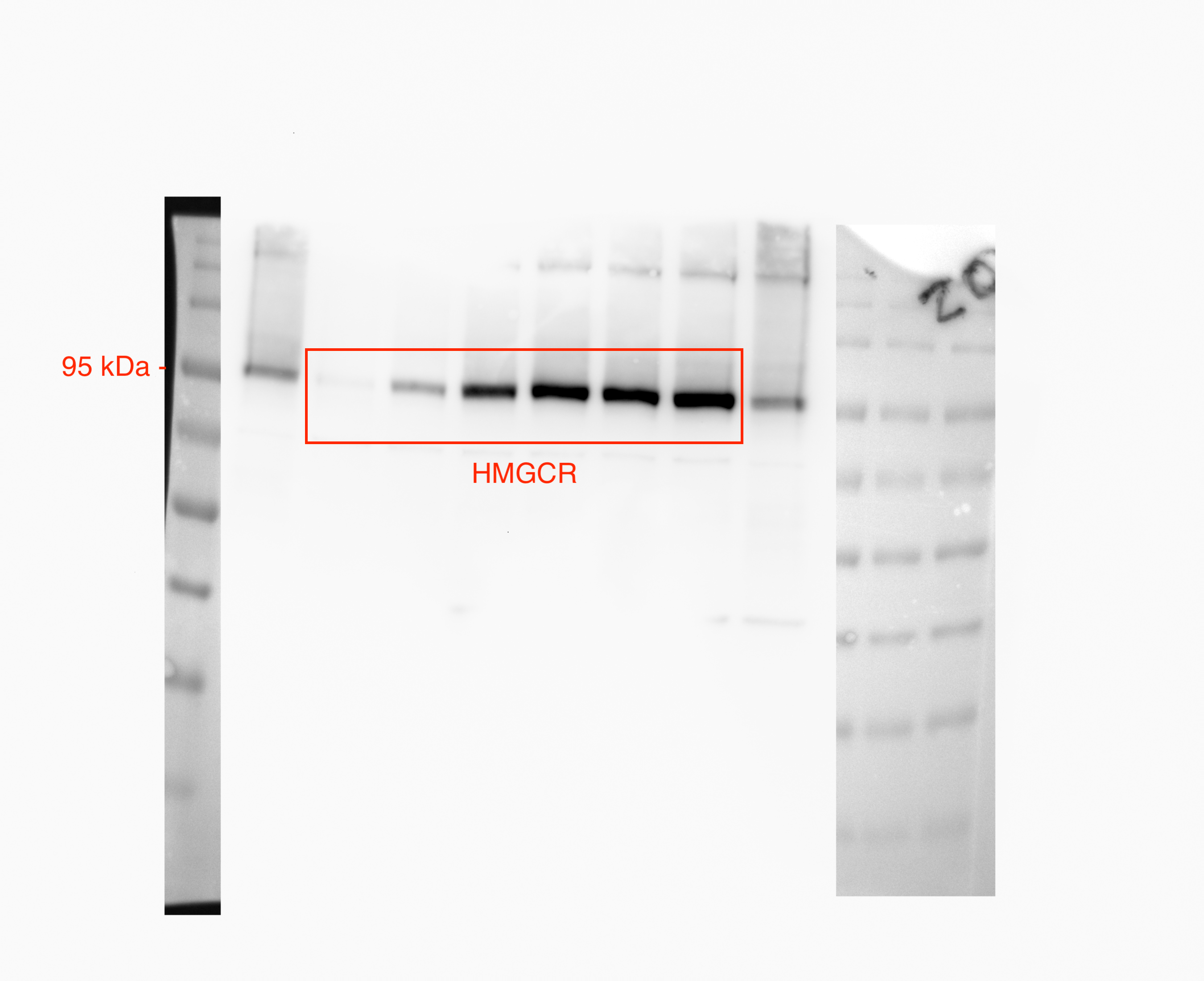

Supplement: Supplementary file 10 — Source data Fig. 6 [file 44318_2024_269_MOESM10_ESM.zip › Figure 6/6A/HMGCR.tiff]

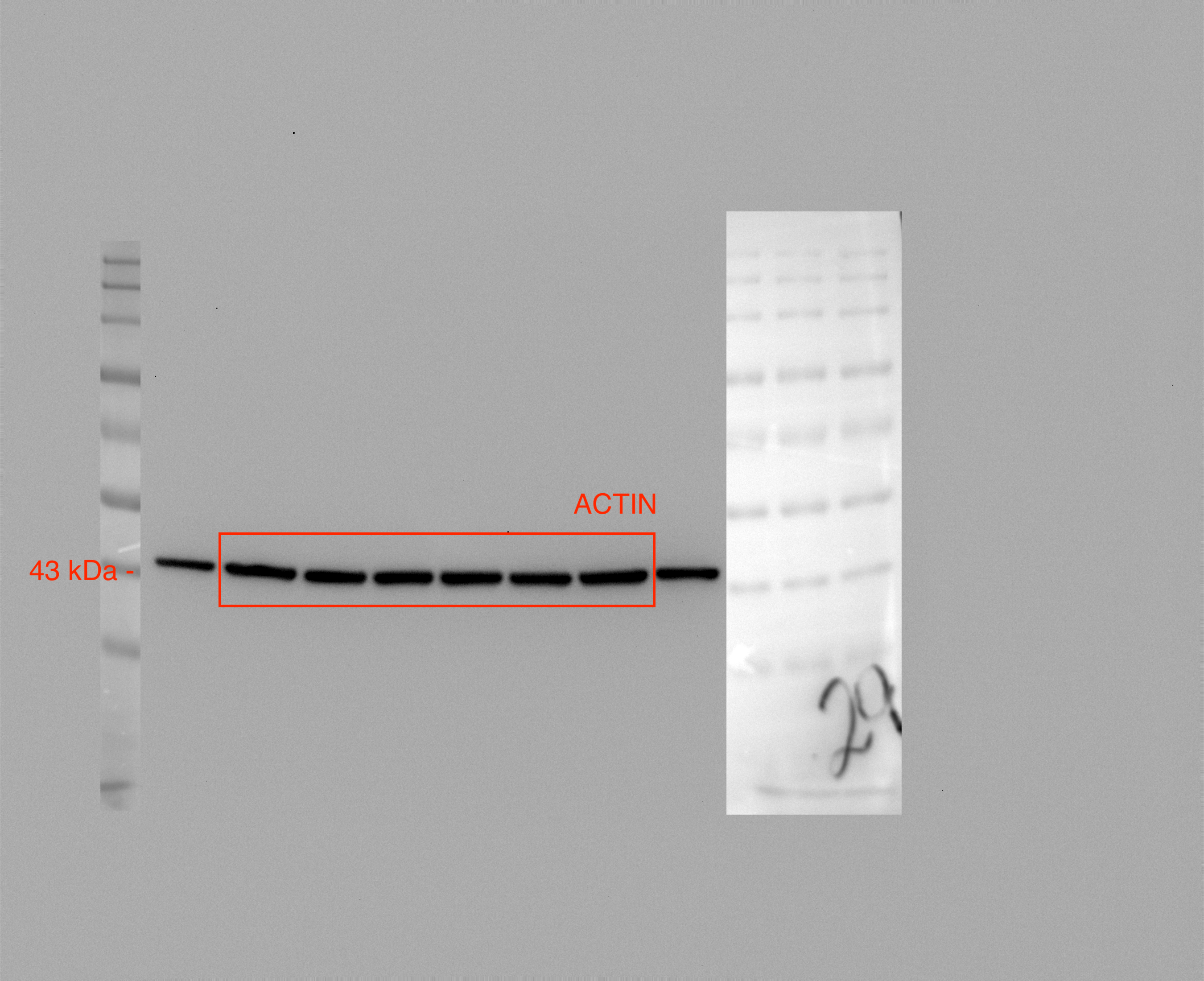

Supplement: Supplementary file 10 — Source data Fig. 6 [file 44318_2024_269_MOESM10_ESM.zip › Figure 6/6A/ACTIN.tiff]

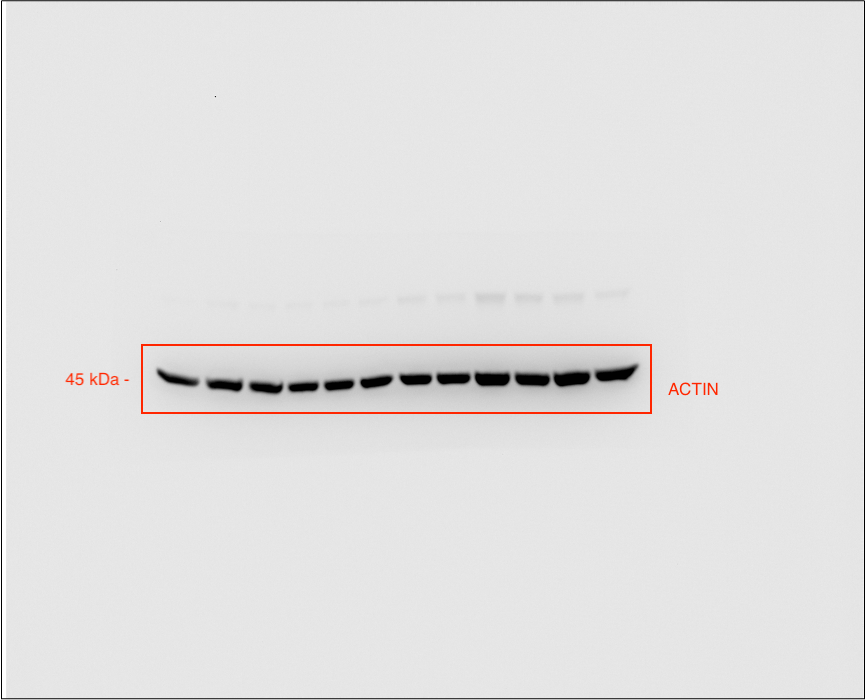

Supplement: Supplementary file 10 — Source data Fig. 6 [file 44318_2024_269_MOESM10_ESM.zip › Figure 6/6O/actin.png]

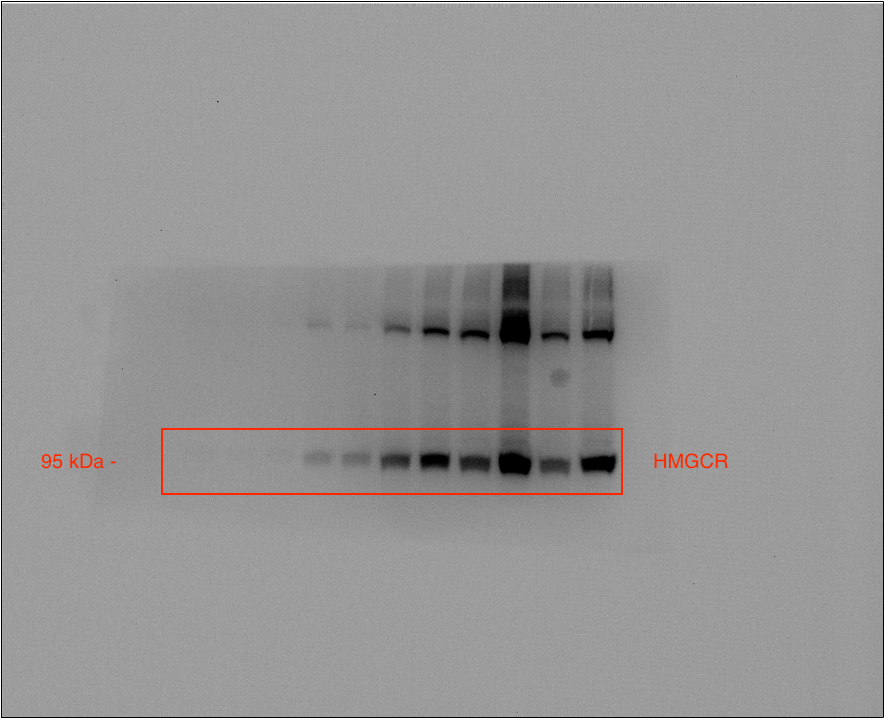

Supplement: Supplementary file 10 — Source data Fig. 6 [file 44318_2024_269_MOESM10_ESM.zip › Figure 6/6O/hmgcr.png]

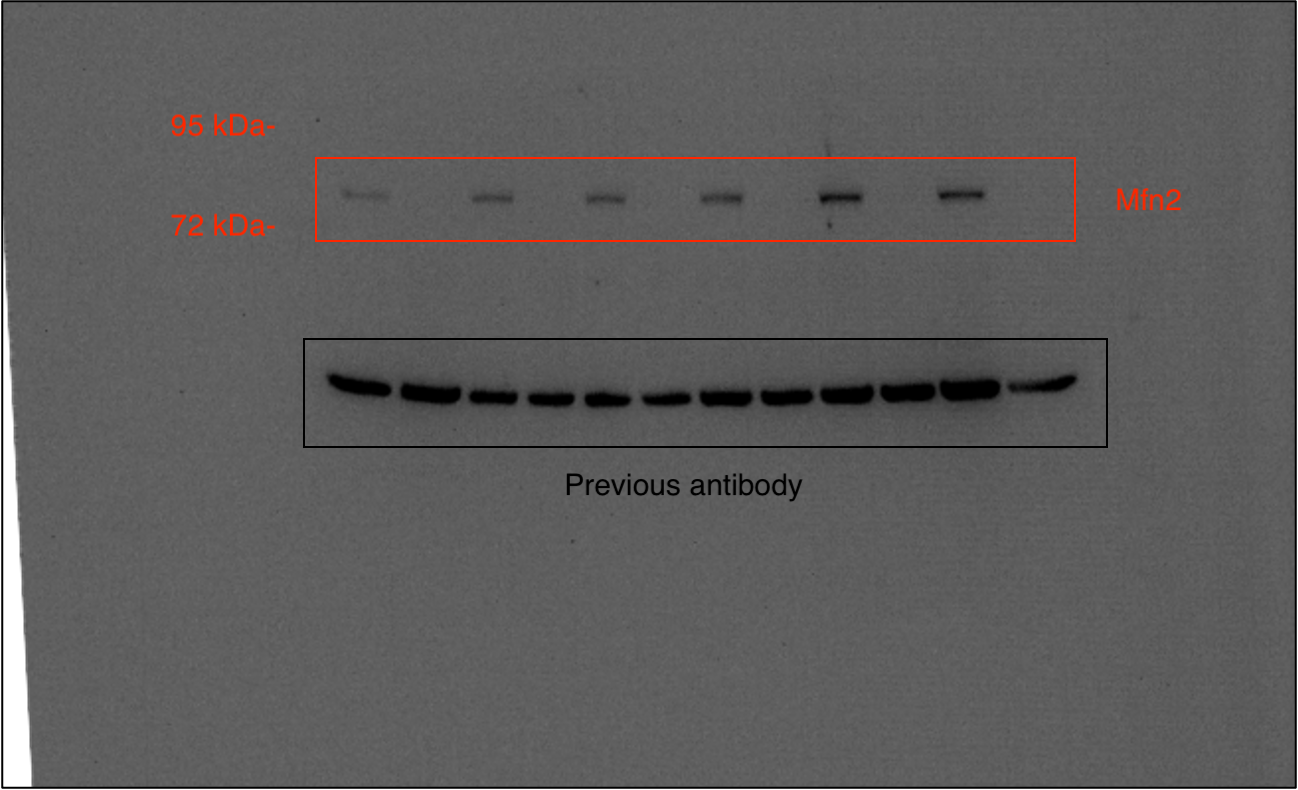

Supplement: Supplementary file 10 — Source data Fig. 6 [file 44318_2024_269_MOESM10_ESM.zip › Figure 6/6O/mfn2.png]

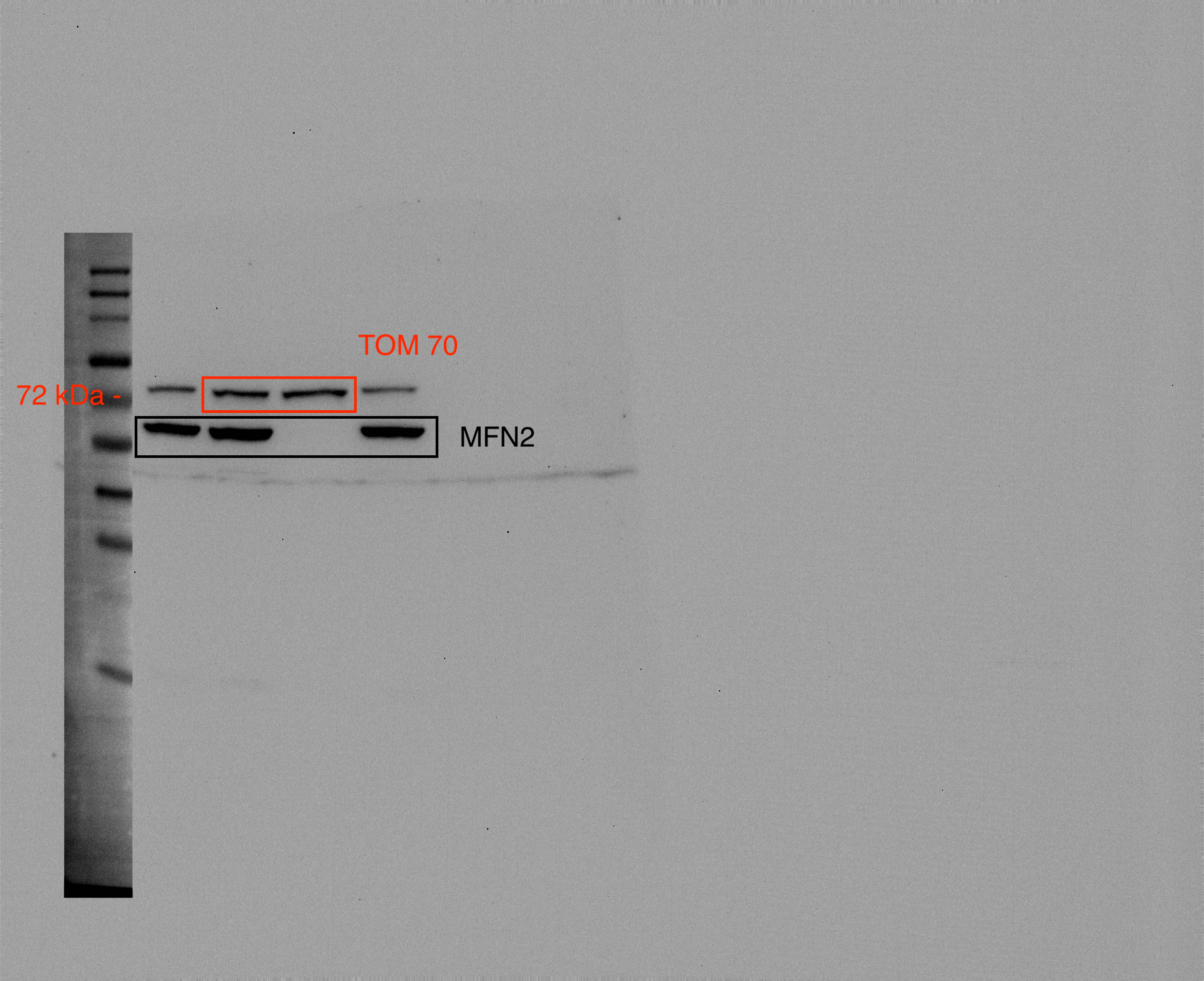

Supplement: Supplementary file 10 — Source data Fig. 6 [file 44318_2024_269_MOESM10_ESM.zip › Figure 6/6B/tom70.tiff]

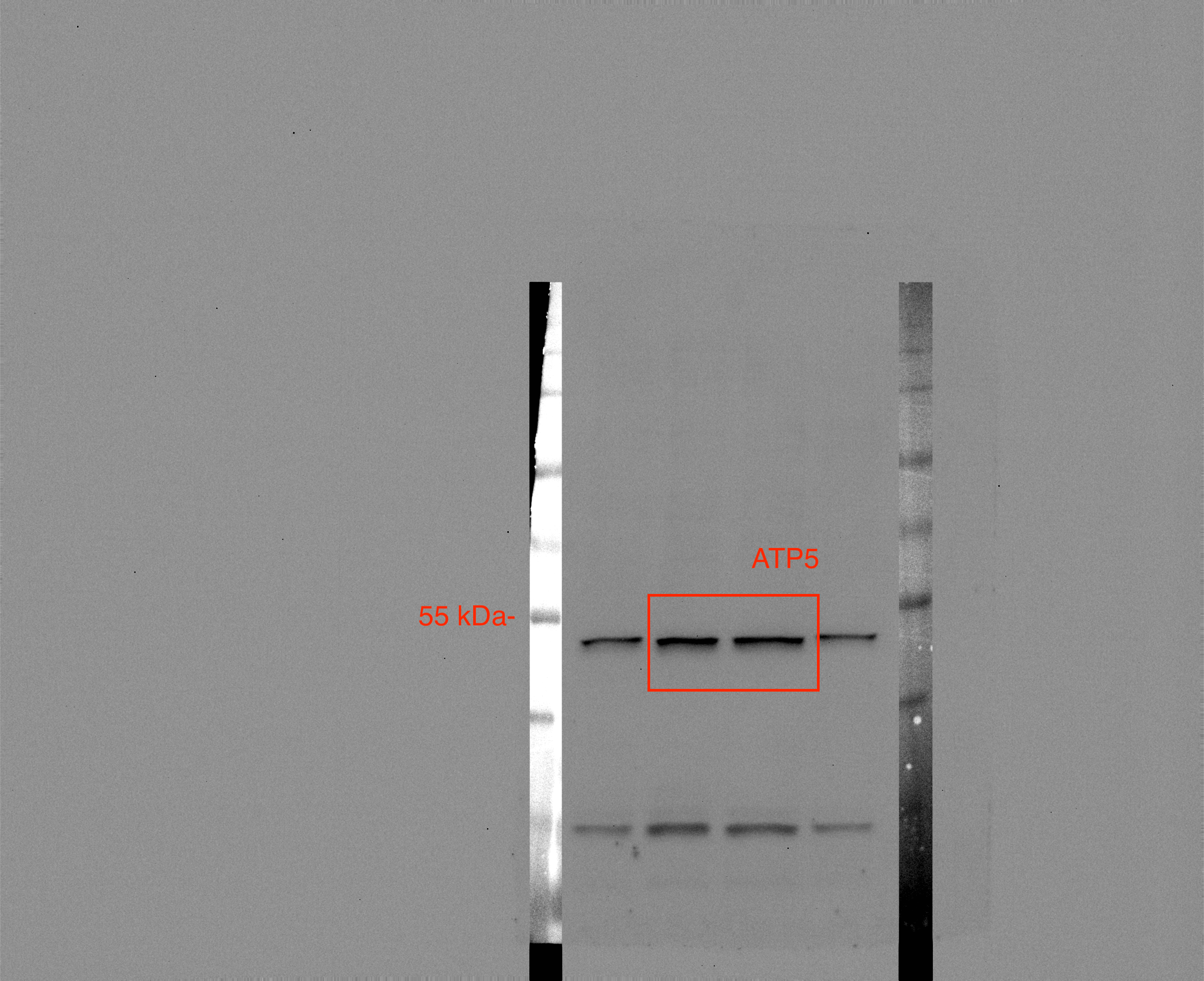

Supplement: Supplementary file 10 — Source data Fig. 6 [file 44318_2024_269_MOESM10_ESM.zip › Figure 6/6B/ATP5.tiff]

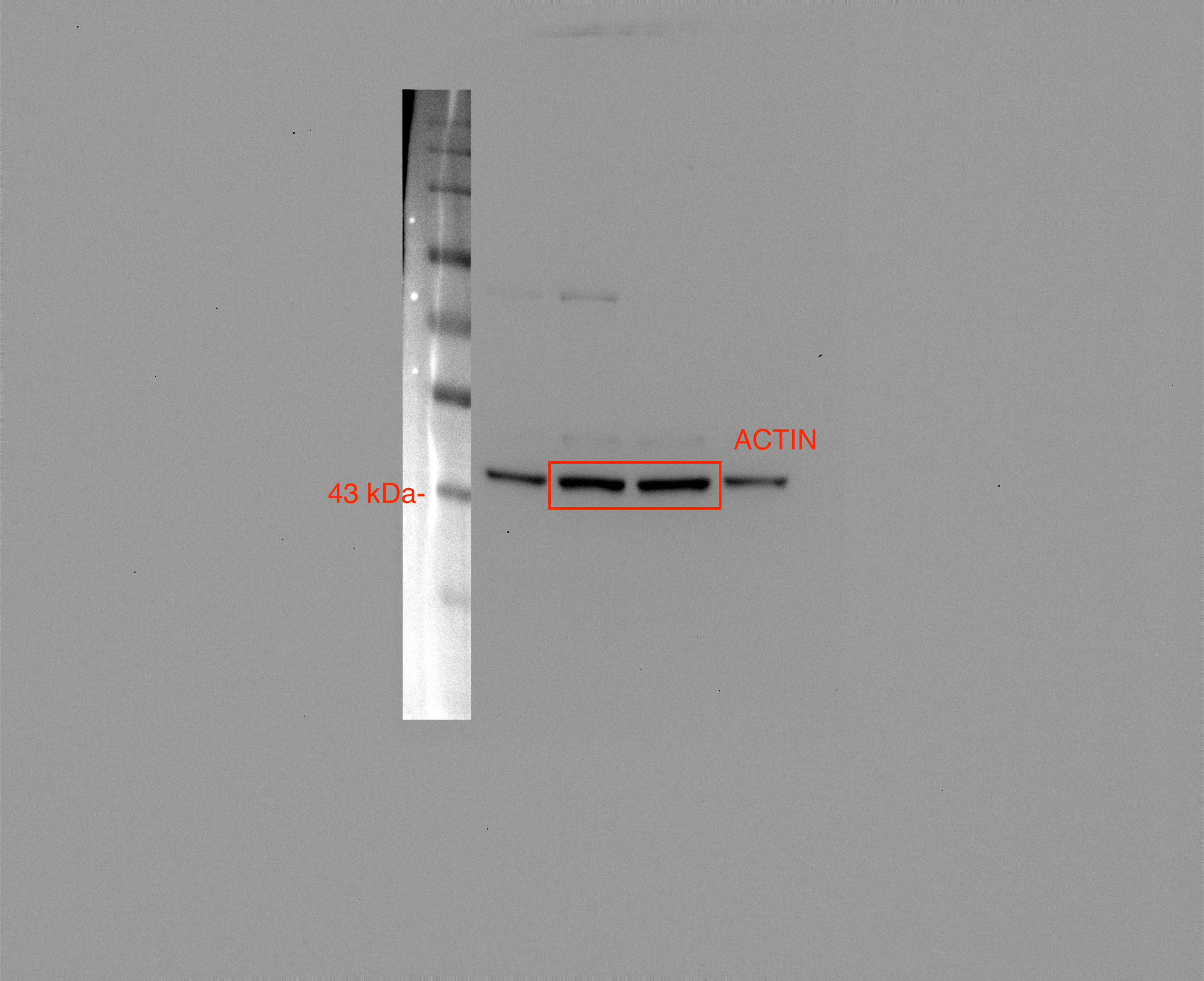

Supplement: Supplementary file 10 — Source data Fig. 6 [file 44318_2024_269_MOESM10_ESM.zip › Figure 6/6B/actin.tiff]

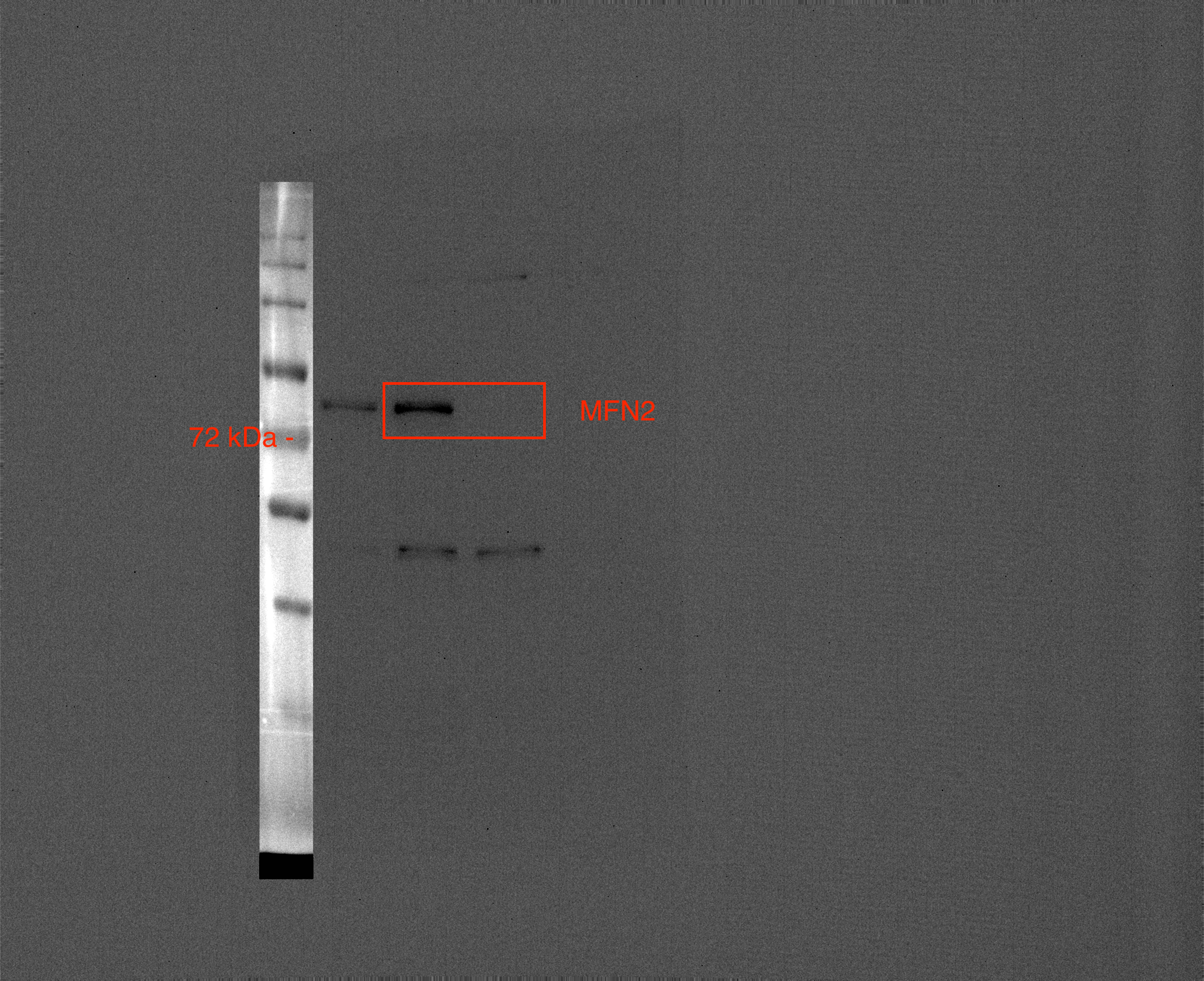

Supplement: Supplementary file 10 — Source data Fig. 6 [file 44318_2024_269_MOESM10_ESM.zip › Figure 6/6B/MFN2.tiff]

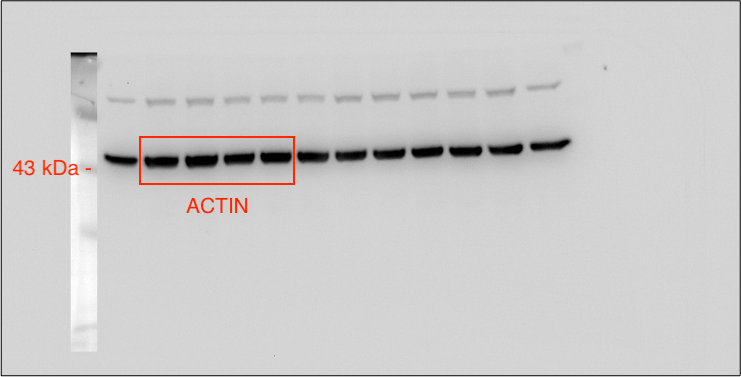

Supplement: Supplementary file 10 — Source data Fig. 6 [file 44318_2024_269_MOESM10_ESM.zip › Figure 6/6L/ACTIN.png]

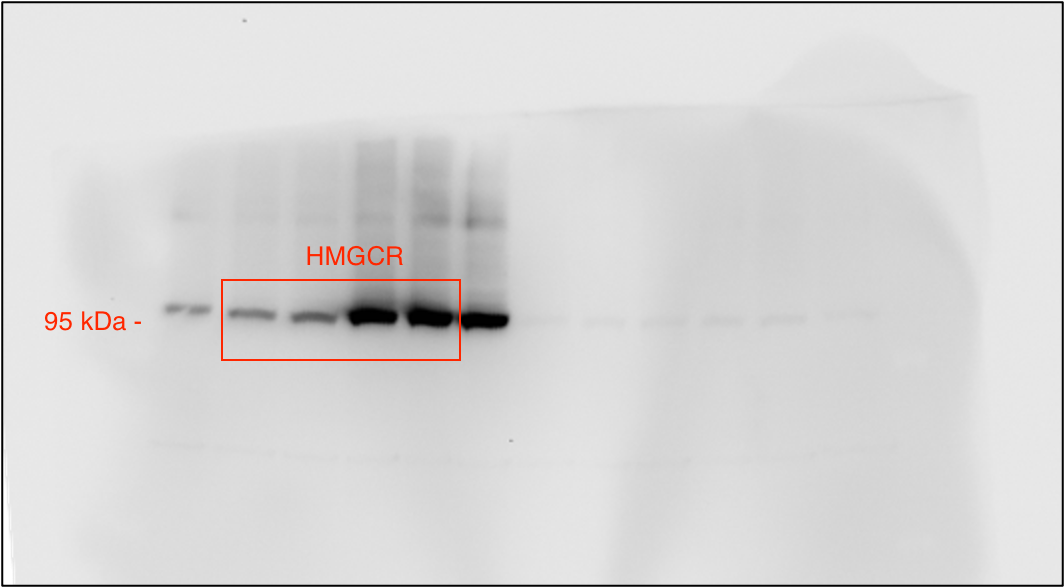

Supplement: Supplementary file 10 — Source data Fig. 6 [file 44318_2024_269_MOESM10_ESM.zip › Figure 6/6L/HMGCR.png]

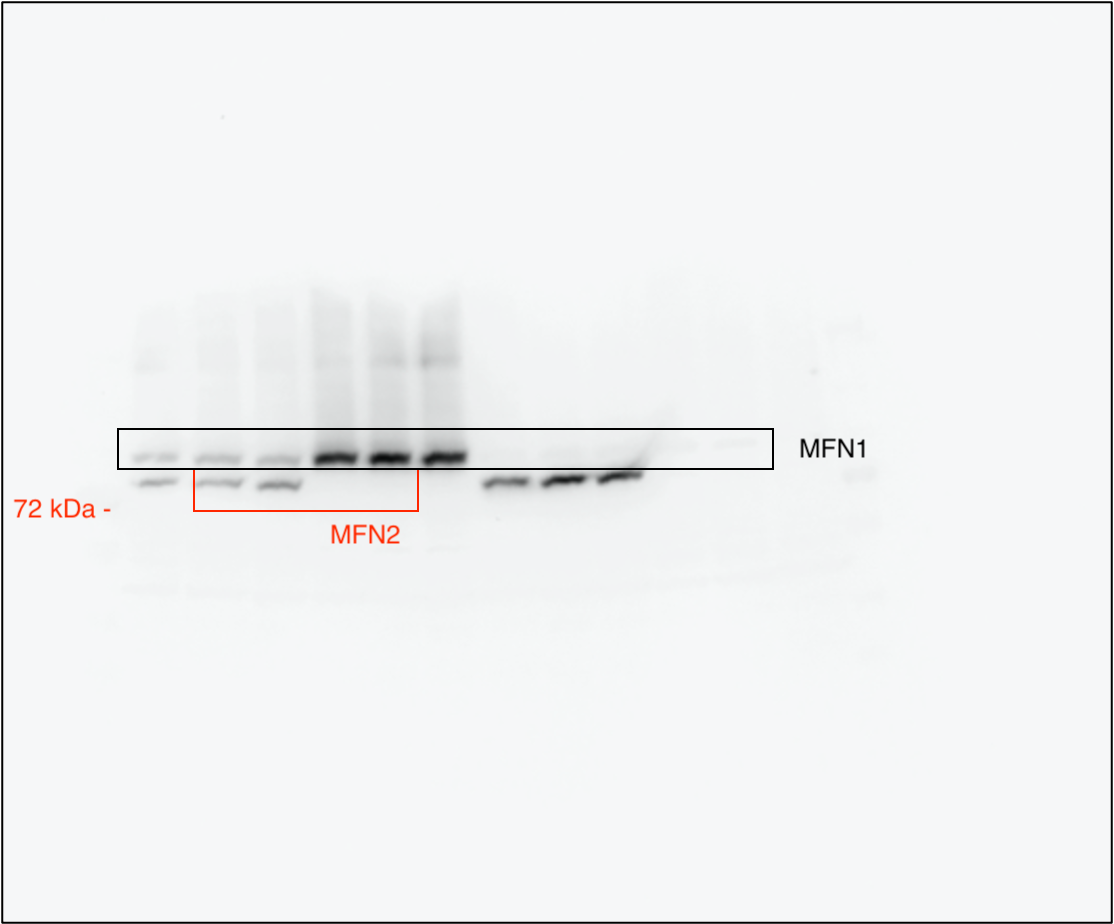

Supplement: Supplementary file 10 — Source data Fig. 6 [file 44318_2024_269_MOESM10_ESM.zip › Figure 6/6L/mfn2.png]

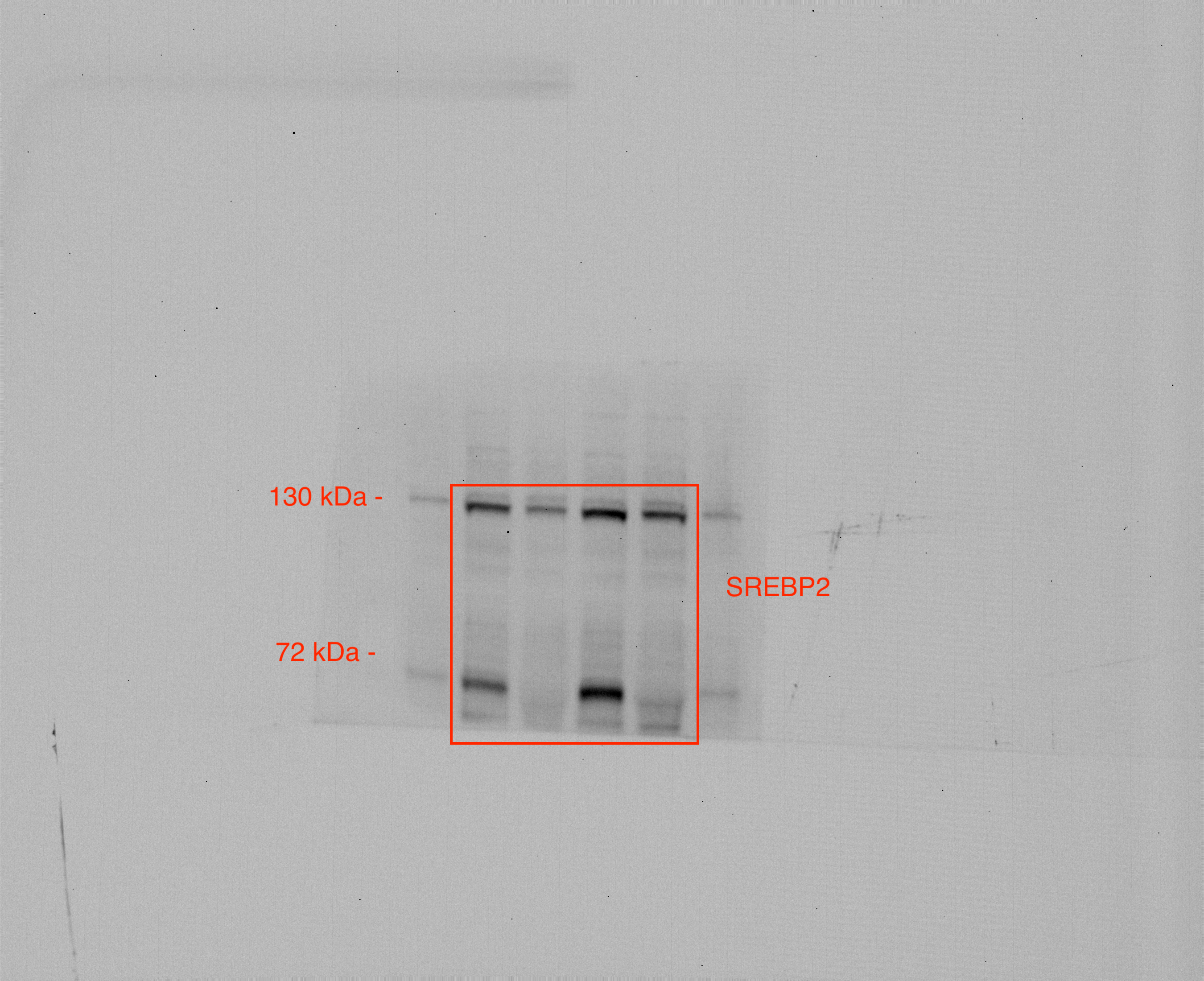

Supplement: Supplementary file 10 — Source data Fig. 6 [file 44318_2024_269_MOESM10_ESM.zip › Figure 6/6M/srebp2 .tiff]

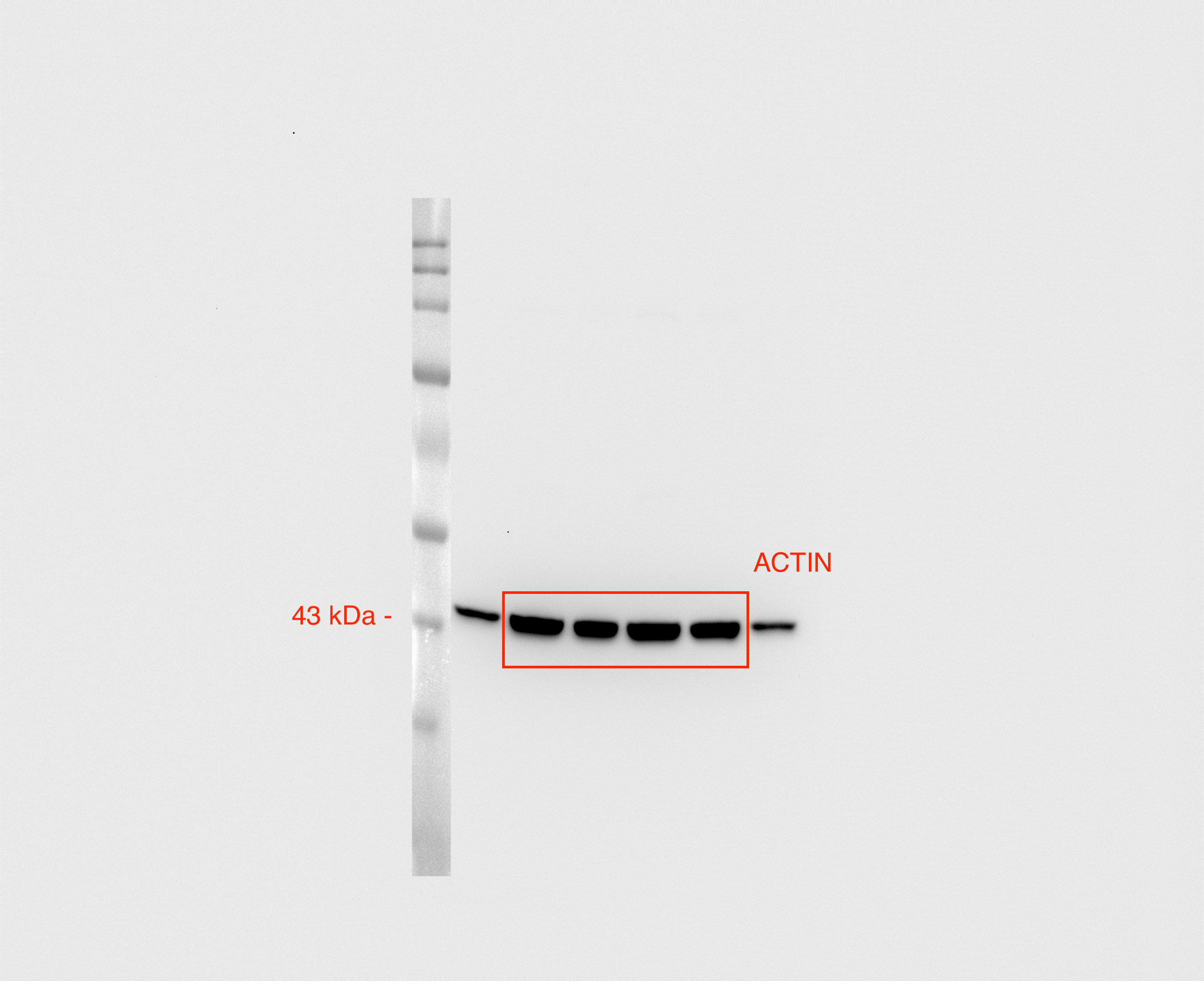

Supplement: Supplementary file 10 — Source data Fig. 6 [file 44318_2024_269_MOESM10_ESM.zip › Figure 6/6M/ACTIN.tiff]

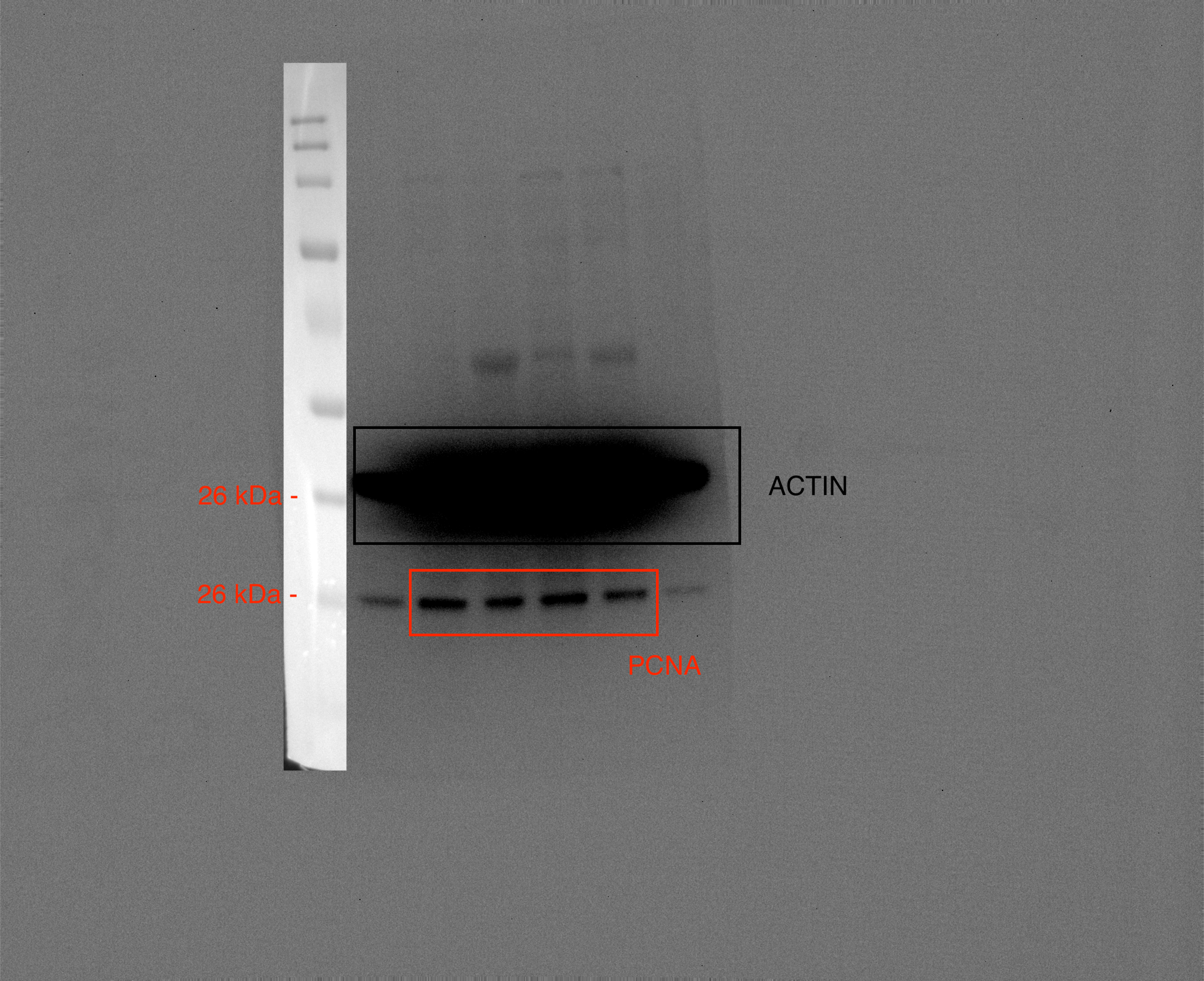

Supplement: Supplementary file 10 — Source data Fig. 6 [file 44318_2024_269_MOESM10_ESM.zip › Figure 6/6M/PCNA.tiff]

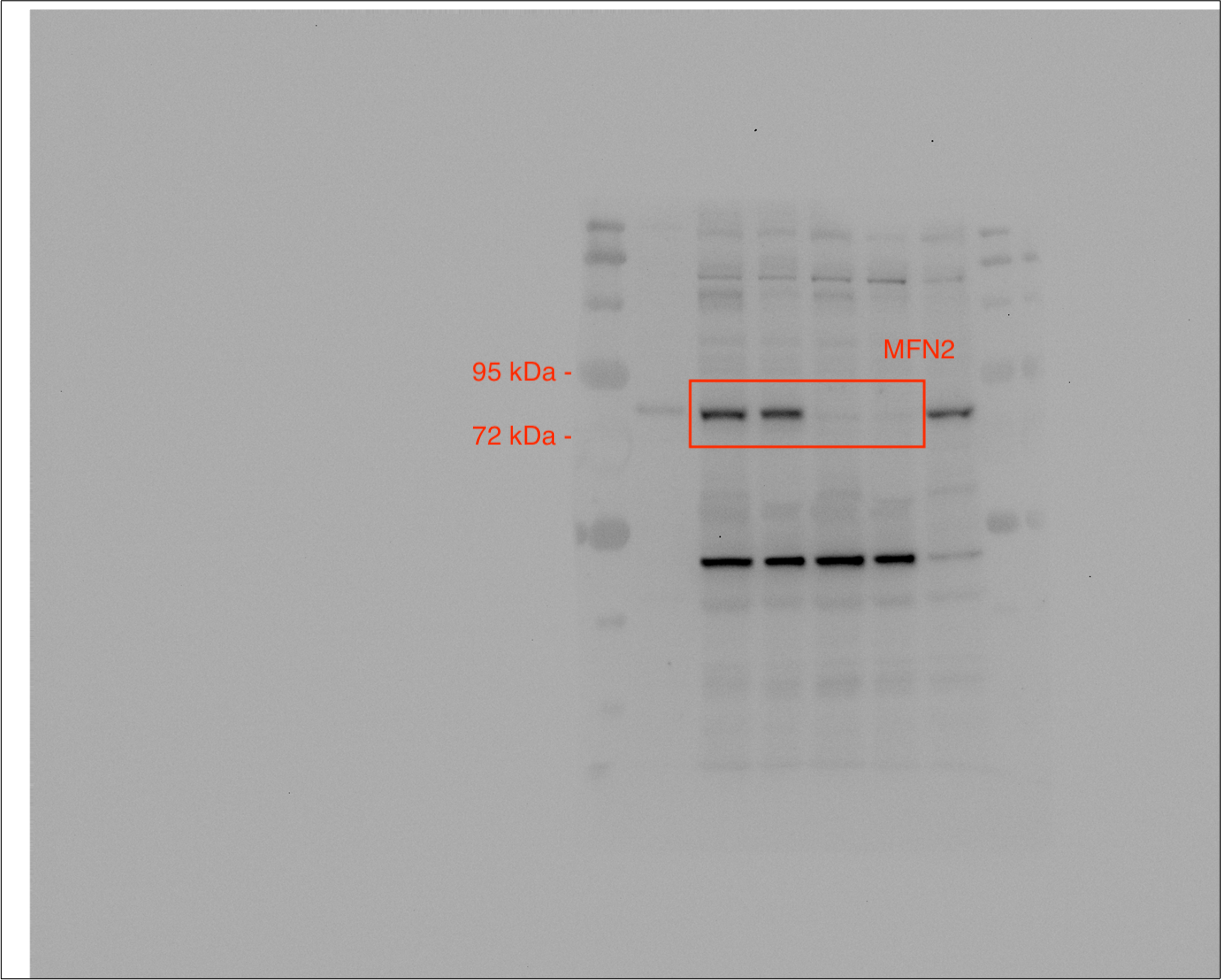

Supplement: Supplementary file 10 — Source data Fig. 6 [file 44318_2024_269_MOESM10_ESM.zip › Figure 6/6M/MFN2.png]
